# A dual Keap1 and p47^phox^ inhibitor Ginsenoside Rb1 ameliorates high glucose/ox-LDL-induced endothelial cell injury and atherosclerosis

**DOI:** 10.1038/s41419-022-05274-x

**Published:** 2022-09-26

**Authors:** Zi-Chao Wang, Kai-Ming Niu, Yu-Jie Wu, Kai-Rui Du, Lian-Wen Qi, Ye-Bo Zhou, Hai-Jian Sun

**Affiliations:** 1 State Key Laboratory of Natural Medicines, School of Traditional Chinese Pharmacy, China Pharmaceutical University, Nanjing, 210009 China; 2 School of Traditional Chinese Pharmacy, China Pharmaceutical University, No. 639 Longmian Road, Nanjing, 211198 China; 3 Clinical Metabolomics Center, China Pharmaceutical University, Nanjing, China; 4 Department of Physiology, Nanjing Medical University, Nanjing, 211166 China; 5 Department of Basic Medicine, Wuxi School of Medicine, Jiangnan University, Wuxi, 214122 China

**Keywords:** Atherosclerosis, Diabetes complications

## Abstract

Oxidative stress is a vital contributor to the development and progression of diabetes-accelerated atherosclerosis. Nuclear factor erythroid 2-related factor 2 (Nrf2) is a well-known molecule that participates in cellular defense against oxidative stress. Utilizing luciferase reporter assay from 379 natural products, we reported here that Ginsenoside Rb1 played a dual role in inhibiting Kelch-like ECH-associated protein 1 (Keap1) and p47^phox^ luciferase reporter activities. In endothelial cells (ECs), Rb1 pretreatment enhanced cell viability, reduced oxidative stress, inflammation, endothelial-mesenchymal transition (EndMT), and apoptosis, as well as ameliorated mitochondrial quality following oxidized low-density lipoprotein (ox-LDL) plus high glucose (HG) challenge. Rb1 directly bound to Keap1 and promoted its ubiquitination and proteasomal degradation dependent on lysine residues (K108, K323, and K551) by recruiting the E3 ligase synovial apoptosis inhibitor 1 (SYVN1), leading to Nrf2 dissociation from Keap1, Nrf2 nuclear translocation, Nrf2/PGC-1α complex formation. We further identified that Rb1 could bind to p47^phox^ and reduce its phosphorylation and membrane translocation, thereby disrupting the assembly of the NOX2 complex. Importantly, Rb1-mediated preservation of cytoplasmic p47^phox^ stabilized and contributed to Nrf2 activation. Additionally, we revealed that Rb1 reduced aortic atherosclerotic plaque formation along with reductions in oxidative stress and inflammatory response in streptozotocin (STZ)-induced ApoE^−/−^ mice, but not in ApoE^−/−^ mice with deficiency of Nrf2 and PGC-1α. Collectively, we demonstrated that Rb1, which directly targeted Keap1 and p47^phox^ in ECs, may be an attractive candidate for the treatment of atherosclerosis in diabetes.

## Introduction

Atherosclerosis is one of the macrovascular complications in diabetes, and diabetes is a leading risk factor for the development and progression of atherosclerotic diseases [1, 2]. Mounting evidence implies that oxidative stress and inflammatory response in endothelial cells (ECs) are central players in the pathogenesis of diabetes-related complications, including atherosclerosis [3]. Therapeutic targeting of oxidative stress in the endothelium might function as a promising strategy to lower endothelial dysfunction and hence diabetes-accelerated atherosclerosis. Nuclear factor erythroid 2-related factor 2 (Nrf2) has been well established to serve as an endogenous antioxidant with tremendous therapeutic benefits in oxidative stress-related disorders [4]. Under physiological conditions, Nrf2 is preserved in cytoplasm and persistently degraded by binding to Kelch-like ECH-associated protein 1 (Keap1). Upon exposure to oxidative and/or electrophilic stimuli, Nrf2 is activated via escaping Keap1-mediated repression and enters into the nucleus to bind to antioxidant response elements (ARE), resulting in the expressions of several antioxidant proteins, one of the most important of which is heme oxygenase 1 (HO-1) [5, 6]. During the process of oxidative stress, nicotinamide adenine dinucleotide phosphate (NADPH) oxidases (NOX) are described to be a major source of reactive oxygen species (ROS) production [7]. NOX2 is believed to be the primary source of ROS in cardiovascular cells wherein the phosphorylation and membrane import of p47^phox^ are the key steps in the assembly of NOX2 complex and following activation of this enzyme [8, 9]. Of interest, the cytosol p47^phox^ physically binds to Nrf2 and increases nuclear accumulation of Nrf2, leading to upregulations of Nrf2-dependent genes [10]. Concordantly, activation of the Nrf2 pathway by disrupting the Keap1/Nrf2 complex and enhancing the p47^phox^/Nrf2 complex might be a prospective approach for the prevention and treatment of diabetes-associated complications, such as atherosclerosis.

Burgeoning evidence suggests that natural products have been used for the treatment of various diseases due to their antioxidant and anti-inflammatory effects, including diabetic atherosclerosis [2]. In this study, we gathered 379 natural compounds to detect their effects on the luciferase reporter gene activities of Keap1 and p47^phox^, respectively, and found that Ginsenoside Rb1 possessed the strong capability to simultaneously inhibit the luciferase reporter gene activities of Keap1 and p47^phox^, suggestive of the antioxidant defense activity of Rb1. Based on this, we investigated the protective role of Rb1 in EC injury and atherosclerotic plaque formation in the context of diabetes.

## Materials and methods

### Reagents and chemicals

Ginsenoside Rb1 (HY-N0039), MG-132 (HY-13259), chloroquine (HY-17589A), cycloheximide (CHX, HY-12320) were purchased from MedChemExpress (Princeton, NJ, USA). D-glucose (NIST917C), dihydroethidium (DHE, 38483-26-0), and streptozocin (STZ, S0130) were bought from Sigma (St Louis, MO, USA). Human oxidized low-density lipoprotein (ox-LDL, 20605ES05) and Luciferase Reporter Gene Assay Kit (11401ES80) were procured from YEASEN (Shanghai, China). Malondialdehyde (MDA, A003-1-2) kit and Cell Counting Kit-8 (CCK-8, G021-1-1) were obtained from Nanjing Jiancheng Bioengineering Institue (Nanjing, China). Annexin V-PE apoptosis detection kit (C1065L), mitochondrial membrane potential detection kit (JC-1, C2006) and cell cycle detection kit (C1052) were acquired from Beyotime Biotechnology (Shanghai, China). MonolithTM RED-NHS Second Generation Protein Labeling Kit (MO-L011) was purchased from NanoTemper (Munich, Germany). PGL3-basic-H-Keap1 luciferase reporter gene plasmids, PGL3-basic-H-p47^phox^ luciferase reporter gene plasmids, GPAAV-tie-eGFP-5′mir30-Mouse-Nfe2l2(Nrf2)-shRNA3-3′mir30-tie enhancer CW3SL and GPAAV-tie-eGFP-5′mir30-Mouse-Ppargc1a(PGC1-α)-shRNA3-3′mir30-tie enhancer CW3SL were produced by Genomeditech (Shanghai, China). Fetal bovine serum (FBS, F8318), and 1% penicillin streptomycin mixture (P4333), were purchased from Sigma (St Louis, MO, USA). Control siRNA (sc-37007), Nrf2 siRNA (sc-37030), and PGC-1α siRNA (sc-38884) were purchased from Santa Cruz (Santa Cruz, CA, USA). Click-iT Plus terminal deoxynucleotidyl transferase dUTP nick end labeling (TUNEL) Alexa Fluor™ 594, Click-iT™ Plus EdU (5-ethynyl-2′-deoxyuridine) Alexa Fluor™ 488 imaging kits (C10637), MitoSOX™ Red Mitochondrial Superoxide Indicator (M36008), nonyl acridine orange (NAO, A1372) and Hoechst 33342 (C10246) were procured from Thermo Fisher Scientific (Carlsbad, CA, USA). His-Keap1 (WT) plasmid, His-Keap1 (Tyr525A mutation) plasmid, His-Keap1 (Ser508A mutation) plasmid, His-Keap1 (Arg415A mutation) plasmid, His-Keap1 (Asp573A mutation) plasmid, His-Keap1 (Val467 mutation) plasmid, His-p47^phox^ (WT) plasmid, His-p47^phox^ (Thr4A) plasmid, His-p47^phox^ (Gln33A) plasmid, His-p47^phox^ (Arg121A) plasmid were created by gene synthesis and site-directed mutagenesis from Jiman biology (Nanjing, China). NCF1 fusion protein (Ag28089) and Keap1 fusion protein (Ag16693) were obtained from Proteintech (Rosemont, IL, USA). Flag-Keap1, Flag-CBL, Flag-SYVN1, Flag-PJA2, Flag-MIB2, Flag-BRCA1, His-Keap1 (K39A), His-Keap1 (K97A), His-Keap1 (K108A), His-Keap1 (K323A), His-Keap1 (K551A) were constructed by gene synthesis and site-directed mutagenesis. HA-Ub plasmid was purchased from Kelei Technology (Shanghai, China). The information of primary and secondary antibodies was provided in Supplementary Table 1. A total of 379 natural products were purchased from TopScience (Shanghai, China), and the information of these compounds was listed in Supplementary Table 2.

### Animals

All experiments in mice were accomplished in compliance with the Care and Use of Laboratory Animals published by the United States National Institutes of Health (NIH Publication, revised 2011) and China Pharmaceutical University, and performed according to the ethical standards laid down in the 1964 Declaration of Helsinki and its later amendments. Male ApoE^−/−^ mice, on a C57BL/6 background, and the age-matched C57BL/6 mice were purchased from Hangzhou Ziyuan Laboratory Animal Science and Technology Co, Ltd. (China). All mice were raised in a temperature-controlled facility on a 12 h light-dark cycle with free access to food and water. After 1 week of acclimation, ApoE^−/−^ mice were intraperitoneally injected with STZ (50 mg/kg) dissolved in citric acid buffer (0.1 mM, pH 4.5) daily for 5 days to induce diabetes, while the control mice received the same volume of citric acid buffer. Two weeks after injection of STZ, the fasting blood glucose (FBG) in each mouse was measured by the Roche Accu-Chek Active blood glucose monitor and the levels of FBG more than 16.7 mM in mice were indicative of diabetes. Then the diabetic mice were administrated with Rb1 (50 mg/kg/day, i.p.) under a high-fat diet (HFD) (60% kcal from fat; D12492; Research Diets, New Brunswick, NJ, USA) for 4 weeks, and the control mice received a normal chow diet (10% kcal from fat; Xietong Organism, China). To determine whether endothelial Nrf2 and PGC-1α mediated the benefits of Rb1 in diabetes-accelerated atherosclerosis in mice, we specifically knockdown the protein of Nrf2 and PGC-1α in the endothelium of ApoE^−/−^ mice by using adeno-associated virus serotype 9 (AAV9) encoding Nrf2 shRNA and PGC-1α shRNA under the control of a Tie2 promoter. The targeting Nrf2 shRNA sequences were 5′-GCTGAAGGCACAATGGAATTC-3′, PGC-1α shRNA sequences were 5′-GCAATAAAGCGAAGAGCATTT-3′, and the control shRNA sequences were 5′-TTCTCCGAACGTGTCACGT-3′. Before STZ injection, the mice received a tail vein injection of AAV9 vectors carrying Nrf2 shRNA, PGC-1α shRNA or control shRNA (5 × 10^11^ viral particles per animal) under the supervision of the Tie2 promoter via tail vein before STZ injection. The body weight and fasting blood glucose were measured at the end of experiments before sacrifice, and the blood, heart, and aortas were collected for histological and biochemical analysis. The plasma levels of triglycerides (TG), total cholesterol (TC), and low-density lipoprotein cholesterol (LDL-C) were enzymatically analyzed using commercial kits (Nanjing Jiancheng Bioengineering Institue, Nanjing, China) in accordance with the manufacturer’s protocols.

### Histological examination

To evaluate the lesion formation of atherosclerosis, the en face aortas attached to the heart of mice were dissected longitudinally and stained with Oil Red O staining (D027-1-3, Nanjing Jiancheng Bioengineering Institue, Nanjing, China). Moreover, the hearts were dissected from the aorta and embedded in Tissue-Tek OCT compound (Sakura Finetek), and the frozen aortic roots were sectioned and stained with Oil Red O staining. In addition, the hearts of mice were fixed in 4% paraformaldehyde, after which paraffin sections and frozen sections of the aortic root were made separately. Paraffin sections were dewaxed, rehydrated and then stained with hematoxylin and eosin (Nanjing Jiancheng, China). Atherosclerotic plaque lesions within the sinus were photographed and quantitated using ImagePro Plus software.

### Cellular culture

Human umbilical vein endothelial EA hy926 cells (Procell Life Science & Technology, China) were cultured in Dulbecco’s modified Eagle’s medium (DMEM, KeyGEN Biotechnology, China) supplemented with 10% FBS (Gibco, USA) at 37 °C in a 5% CO_2_ incubator. Diabetes-promoted atherosclerotic ECs model was induced by the addition of D-glucose (25 mM) and ox-LDL (50 mg/L) into the culture medium in the absence or presence of different concentrations of Rb1 (0, 3, 10, 30 μM) for 24 h. EA.hy926 ECs cultured in DMEM-normal glucose medium (5.5 mM) with 10% FBS were considered as control cells. Primary mouse vascular ECs were cultured and passaged as previously described [11]. HEK293T (ATCC, VA) was maintained in DMEM containing 10% FBS (Gibco, USA), 100 units/ml penicillin, and 100 μg/ml streptomycin under a humid incubator with 5% CO_2_ at 37 °C.

### Cell viability assay

The cell viability of EA hy926 was detected by CCK-8 kit according to the manufacturer’s instructions. Briefly, EA hy926 were seeded on 96-well plates and pretreated with vehicle or different concentrations of Rb1 (0, 3, 10, 30 μM) for indicated time and treated with ox-LDL plus HG for 24 h. After that, 10 μL CCK-8 was added to each well for 2 h incubation at 37 °C and the absorbance was measured at 450 nm by a microtiter plate reader (BioTek Instruments, Inc., Winooski, VT, USA). Furthermore, EdU incorporation experiments were carried out to assess the proliferating cells by quantifying the ratio of EdU-positive cells to the Hoechst 33342-stained cells from six random fields in three or four-independent repetitions.

### Measurement of oxidative stress

Intracellular ROS production was examined using DHE fluorescent probe in sectioned aortas and fixed cells as we previously described [12, 13]. In short, the samples were probed with DHE dye (10 μM) for 1 h at 37 °C under the dark environment. The sections or cells were washed with PBS 3 times. The immunofluorescence images were then photographed. For mitochondrial ROS detection in EA hy926, cells were loaded with MitoSOX Red Mitochondrial Superoxide Indicator (5 μM) at 37 °C for 30 min and then washed with PBS three times. Images were viewed with a fluorescence microscope (Olympus, Japan).

### Measurement of MDA contents

Cell samples were lysed to collect proteins and were quantified with a BCA assay kit (Beyotime, China) for the normalization of assayed samples. Add 0.1 ml of lysate to the 96-well plate as a blank control, add 0.1 ml of standards of different concentrations in the kit for making the standard curve, add 0.1 ml of sample for the determination, then add 0.2 ml of MDA assay working solution to each well and incubate at 100 °C for 15 min, cool to room temperature and then centrifuge (1000 × *g*, 10 min), aspirate 200 µl per well into a new 96-well plate to determine the absorbance value. The final results were normalized to the protein content.

### Cell cycle and apoptosis assays

After treatment, the cells were collected and subjected to flow cytometry analysis using a flow cytometry (Cytoflex LX, Beckman, Indianapolis, IN, USA). Cell cycle detection kits and Annexin V-PE apoptosis detection kits were used to evaluate the cell cycle distribution and apoptosis, respectively, according to the manufacturer’s instructions.

### Western blot analysis

Cell and tissue samples were lysed to collect proteins. Nuclear proteins were prepared using an NE-PER Nuclear and Cytoplasmic Extraction Reagents (ThermoFisher, America) and mitochondrial proteins were obtained with a cellular mitochondrial isolation kit (C3601, Beyotime, Shanghai, China). Cell membrane proteins were obtained with a membrane protein extraction kit (P0033, Beyotime, Shanghai, China). The total protein concentration was quantified with a BCA assay kit (Beyotime, China). Protein extracts were separated on 8–12% sodium dodecyl sulfate polyacrylamide gels and transferred to polyvinylidene fluoride membranes (GVHP04700, Millipore, Darmstadt, Germany). The membranes were blocked by 5% nonfat milk at room temperature for 1 h and then incubated with the indicated primary antibody solution overnight at 4 °C followed by incubation with horseradish peroxidase (HRP)-conjugated secondary antibodies. The mitochondrial proteins were normalized to COX IV, and the membrane proteins were normalized to Na^+^/K^+^ ATPase α1 (NKAα1). The values of band intensities were quantized by Image-Pro Plus 6.0 software.

### Immunoprecipitation

For immunoprecipitation assay, the collected cell samples were lysed in 200 µL IP lysate buffer with 1% protease inhibitor cocktail. The cell lysates were centrifuged (12,000 × *g*, 20 min, 4 °C), and the supernatants were collected for the measurement of protein concentrations in each sample. Equal amount of cell lysates were incubated with IgG or the primary antibodies on a rotator for 2 h at 4 °C and the Protein G PLUS-Agarose (20 μl) was added into the corresponding tubes at 4 °C on a rocker platform. Afterward, the immune complex-containing beads were washed with the PBS for 3 times followed by the addition of 1 × loading buffer and western blotting analysis.

### Quantitative real-time PCR

Total RNA was isolated using Trizol reagent (Yeasen, China) following the manufacturer’s instructions. RNA samples were reverse transcribed to cDNA by using Hifair^®^ III 1st Strand cDNA Synthesis SuperMix for qPCR (Yeasen, China). The relative gene expression was relatively quantified by Hieff^®^ qPCR SYBR Green Master Mix (Low Rox Plus, Yeasen, China). The 2^−ΔΔCt^ method was used for relative quantification of the target gene. The sequences of primers used in this study were listed in Supplementary Table 3.

### Mitochondrial membrane potential and mass assay

Mitochondrial membrane potential in collected EA.hy926 cells was examined by fluorescent dye JC-1. JC-1 is a fluorescent lipophilic carbocyanine dye used to measure mitochondrial membrane potential. When the mitochondrial membrane potential is high, JC-1 aggregates in the matrix to form J-aggregates, which can produce red fluorescence. When the mitochondrial membrane potential is low, JC-1 cannot aggregate in the mitochondrial matrix, the presence of monomers produces green fluorescence. In short, the cells were stained with JC-1 for 30 min at 37 °C, and the green JC monomers (488 nm) and red JC aggregates (570 nm) were visualized by a fluorescence microscope (Olympus, Japan). To assess the mitochondrial mass in ECs, the collected EA.hy926 cells were incubated with NAO (0.1 μM) for 30 min at 37 °C under a dark environment, and the cellular fluorescence was imaged using a fluorescence microscope (Olympus, Japan).

### Immunofluorescence

EA.hy926 cells were seeded in 6-well plates and fixed with 4% paraformaldehyde solution (Biosharp, China) for 20 min, followed by permeabilization with 0.5% Triton X-100 (Beyotime, China). Cells were then incubated with the primary antibodies for overnight at 4 °C. After incubation with secondary antibodies for 60 min and DAPI for 15 min, the cells were analyzed using a fluorescence microscope (Olympus, Japan).

### Cell transfection

EA.hy926 cells were transfected with control siRNA (100 nM), Nrf2 siRNA (100 nM), PGC1-α siRNA (100 nM), three SYVN1 siRNAs (100 nM), or tag plasmid (0.5 μg) or mutant plasmid (0.5 μg) for 24 h using Lipofectamine 2000 (11668019, Invitrogen, Carlsbad, CA, USA) according to the manufacturer’s instructions. The cells were then subjected to the corresponding treatments and following biochemical molecular experiments. The scrambled siRNA sequences were as follows: sense, 5′-UUCUCCGAACGUGUCACGUTT-3′; antisense, 5′-ACGUGACACGUUCGGAGAATT-3′. SYVN1 siRNA1: 5′-GCUUCUCAAGUGAGACUGACC-3′ (sense); 5′-UCAGUCUCACUUGAGAAGCUG-3′ (antisense). SYVN1 siRNA2: 5′-GUUUCAGAUGAUUAUUUAAUU-3′ (sense); 5′-UUAAAUAAUCAUCUGAAACUG-3′ (antisense).

SYVN1 siRNA3: 5′-AGGCUGUGUACAUGCUCUACA-3′ (sense); 5′-UAGAGCAUGUACACAGCCUUG-3′ (antisense).

### Luciferase reporter gene assay

EA.hy926 cells were transfected with the PGL3-basic-H-Keap1 luciferase reporter gene plasmids and PGL3-basic-H-p47^phox^ luciferase reporter gene plasmids for 24 h using Lipofectamine 2000 (11668019, Invitrogen, Carlsbad, CA, USA) according to the manufacturer’s instructions. The cells were then incubated with various compounds (5 μM) for an additional 24 h. Finally, the cells were lysed and the activities of firefly luciferase and renilla luciferase were determined using the Luciferase Reporter Gene Assay Kit (Yeasen, China) according to the manufacturer’s suggestions.

### Molecular docking

The structure of ginsenoside Rb1 (CID: 9898279) was downloaded from Pubchem and then subjected to basic processing using mgtools. The crystal structure of Keap1 (ID: 7k2h) and p47^phox^ (ID: 1kq6) were downloaded from the Protein Data Bank (https://www.rcsb.org/). The docking conformation of ginsenoside Rb1 and Keap1 or p47^phox^ was simulated using AutoDock (version 4.2.6). The grid box was set to the dimensions of 80 × 80 × 80 with grid spacing of 0.375 Å. 1000 individual genetic algorithm were executed to obtain multiple docking conformations to select the optimal conformation.

### SPR analysis

The binding affinity of ginsenoside Rb1 to Keap1 and p47^phox^ proteins were measured using a Biacore T200 (GE Healthcare, America). Human recombinant Keap1 and p47^phox^ proteins were captured on a CM5 chip by a standard amine coupling procedure. Binding sensorgrams were recorded by injecting various concentrations of ginsenoside Rb1 over the immobilized proteins surface. The data were fitted and analyzed to obtain the equilibrium dissociation constant (KD) value.

### MST analysis

Solution MST binding studies were performed using standard protocols on a Monolith NT.115 (NanoTemper, Germany). Briefly, recombinant Keap1 and p47^phox^ proteins were labeled using the RED-NHS (Amine Reactive) Protein Labelling Kit. After that, different concentrations of ginsenoside Rb1 were mixed with the labeled protein and fed into the sample by capillary tube. All experiments were performed with a minimum of 3 replicates.

### Statistical analysis

All results were collected from at least four individual biological replicates and were expressed as the mean ± SE. Statistical significance analyses were performed using GraphPad Prism version 8.0 (GraphPad Software, Inc., La Jolla, CA, USA). Comparisons between two groups were analyzed by using the unpaired two-tailed Student’s *t* test, and those among three or more group comparisons were conducted using analysis of variance (ANOVA). The threshold for statistical significance difference was recognized as *P* values of less than 0.05.

## Results

### Rb1 is identified as an inhibitor for both Keap1 and p47^phox^ in ECs

In response to oxidants or electrophiles, the interaction between Keap1 and Nrf2 is disrupted, leading to the dissociation of Keap1/Nrf2 complex, Nrf2 nuclear translocation, and upregulations of Nrf2-targeted antioxidant genes [14]. NADPH oxidase p47^phox^ in cytosol physically binds to Nrf2 and facilitates the nuclear translocation of Nrf2, thus inducing the expressions of Nrf2-dependent anti-oxidative genes [10]. It is likely that inhibition of Keap1 and/or conservation of p47^phox^ in cytosol may be an alternative strategy to activate the Nrf2 pathway. For this reason, we screened a small molecule pool containing 379 natural products using a luciferase reporter gene assay based on the Keap1 and p47^phox^ promoter activities. Results showed that 36 compounds inhibited the luciferase report gene activities of Keap1 by more than 50%, and 38 compounds suppressed p47^phox^ luciferase report gene activities by more than 50% in ECs (Fig. S1A–D). The Venn diagrams of the top 10 compounds that inhibited both reporter genes, respectively, showed that only Rb1 had a strong ability to simultaneously repress the luciferase activities of Keap1 and p47^phox^ (Fig. S1E).

### Rb1 attenuates EC superoxide production

**Fig. 1:**
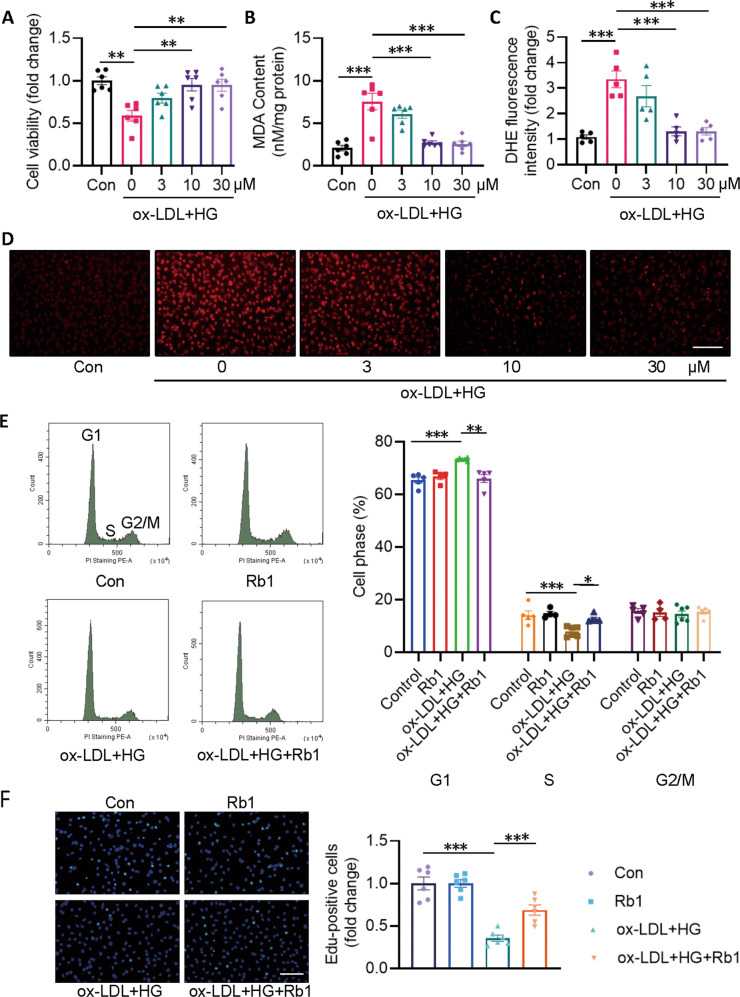
Rb1 protects ECs from ox-LDL/HG-induced cell viability decline and oxidative stress. EA.hy926 ECs were pretreated with Rb1 (3, 10, and 30 μM) for 30 min before ox-LDL/HG for 24 h. **A** Cell viability measured by CCK-8 assay. **B** MDA contents. **C** Quantitative analysis of DHE staining. **D** Representative images of DHE staining. Scale bar = 200 μm. **E** Cell cycle was determined by flow cytometer and the cell cycle distribution was calculated. **F** EdU incorporation experiments and EdU-positive cells were quantified. Scale bar = 200 μm. Data are presented as mean ± SEM. *n* = 4–6. Statistical analysis was performed with the randomized block ANOVA. *P < 0.05; **P < 0.01; ***P < 0.001; *vs*. indicated group.

In light of the strong capabilities of Rb1 in suppressing Keap1 and p47^phox^, it is reasonable to assume that Rb1 is a potential antioxidant with benefits in diabetic atherosclerosis. Results showed that exposure of ECs to Rb1 dose-dependently restored the declined cell viability in ox-LDL/HG-challenged ECs (Fig. 1A). Exposure to ox-LDL/HG promoted the generation of MDA, a marker of oxidative stress, while this was concentration-dependently reversed by Rb1 pretreatment (Fig. 1B). Similar results were observed when ECs were visualized by DHE staining (Fig. 1C, D). Accordingly, we selected 10 μM of Rb1 as the working concentration in the following experiments. Cell cycle distribution displayed that ox-LDL/HG increased G1 phase proportion, but decreased S phase proportion in ECs, an effect that was reversed by Rb1 treatment (Fig. 1E). The ability of Rb1 to promote cell proliferation was further confirmed by EdU staining as Rb1 increased the number of EdU-positive cells after ox-LDL/HG treatment (Fig. 1F). We further examined the cell viability and oxidative stress after ox-LDL/HG insult under the conditions of post-treatment of Rb1. Similar to the results of pretreatment with Rb1 for 30 min (Fig. S2A), treatment of cells with Rb1 3 h after exposure to ox-LDL/HG also prevented the decreased cell viability in ECs (Fig. S2B). Nevertheless, this reversal of cell viability changes by Rb1 disappeared when Rb1 was administered after 12 h following exposure to ox-LDL/HG (Fig. S2C), indicating that Rb1 may be used to prevent or treat early diabetic atherosclerosis, but not late-stage diabetic atherosclerosis. Likewise, similar results were also demonstrated by DHE staining (Fig. S2D–F).

### Rb1 attenuates EC apoptosis, EndMT, and inflammation

**Fig. 2:**
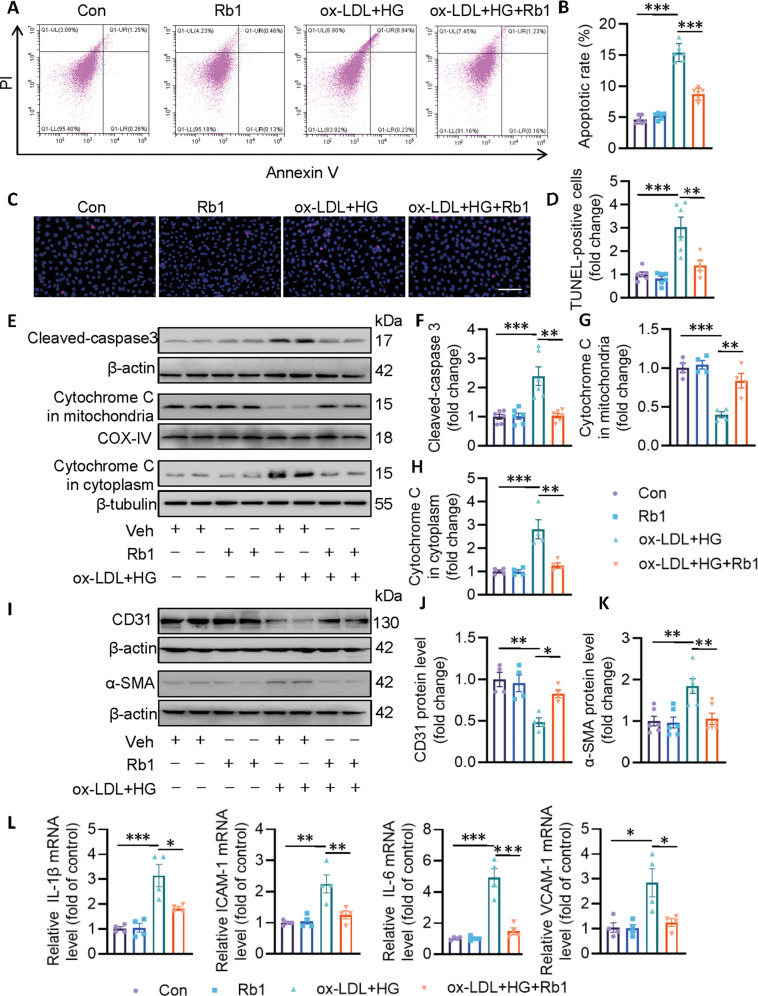
Rb1 protects ECs from ox-LDL/HG-induced cell apoptosis, EndMT, and inflammation. EA.hy926 ECs were pretreated with Rb1 (10 μM) for 30 min before ox-LDL/HG for 24 h. **A** Cell apoptosis measured by flow cytometer. **B** Apoptosis ratio. **C** Cell apoptosis measured by TUNEL assay. Scale bar = 200 μm. **D** Relative analysis of TUNEL-positive cells. **E**–**H** Representative blots and quantitative analysis of cleaved-caspase3, mitochondrial cytochrome c, cytoplasmic cytochrome c. **I**–**K** Representative blots and quantitative analysis of CD31 and α-SMA. **L** Relative mRNA levels of *IL-1β*, *ICAM-1*, *IL-6*, and *VCAM-1*. Data are presented as mean ± SEM. *n* = 4–6. Statistical analysis was performed with the randomized block ANOVA. *P < 0.05; **P < 0.01; ***P < 0.001; *vs*. indicated group.

Next, we examined the effects of Rb1 on EC apoptosis, EndMT, and inflammation after exposure to ox-LDL/HG. Flow cytometry results showed greater staining in the presence of ox-LDL/HG, indicative of more EC apoptosis, which effect was prevented in ECs pretreated with Rb1 (Fig. 2A, B). TUNEL staining showed that ox-LDL/HG triggered EC apoptosis, as evidenced by increased TUNEL-positive cells, while this apoptotic effect was evidently reduced by Rb1 pre-administration (Fig. 2C, D). Likewise, immunoblotting results showed that ox-LDL/HG promoted the protein expression of cleaved-caspase-3, and facilitated the mitochondrial leakage of cytochrome C, leading to cell apoptosis (Fig. 2E–H). More importantly, such effects were largely attenuated by pretreatment with Rb1 (Fig. 2E–H). In the presence of ox-LDL/HG, the endothelial marker CD31 protein was downregulated and the mesenchymal marker SM22α protein was upregulated, indicating the process of EndMT, which was significantly curbed by Rb1 (Fig. 2I–K). Incubation of ECs with ox-LDL/HG elevated the mRNA levels of pro-inflammatory factors, including *IL-1β*, *ICAM-1*, *IL-6*, and *VCAM-1*, while Rb1 pretreatment protected ECs against the pro-inflammatory effects of ox-LDL/HG (Fig. 2L).

### Rb1 protects against ox-LDL/HG-induced mitochondrial damage

**Fig. 3:**
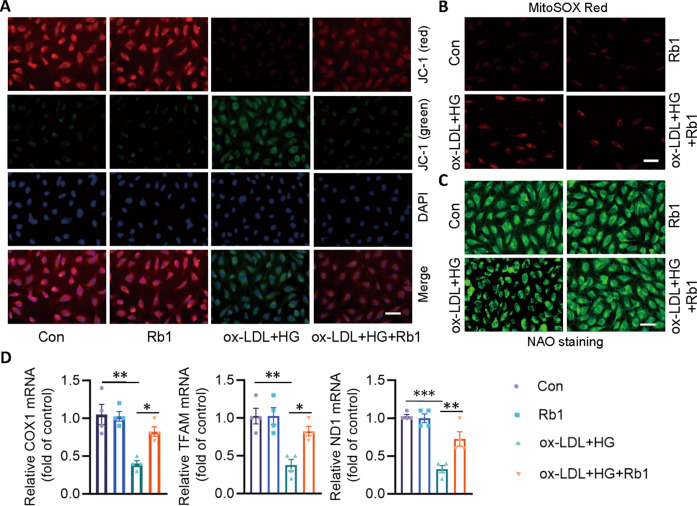
Rb1 protects against ox-LDL/HG-induced mitochondrial damage. EA.hy926 ECs were pretreated with Rb1 (10 μM) for 30 min before ox-LDL/HG for 24 h. **A** Cell membrane potential determined by JC-1 staining. Scale bar = 50 μm. **B** Mitochondrial ROS determined by MitoSOX Red. Scale bar = 50 μm. **C** Mitochondrial mass determined by NAO staining. Scale bar = 50 μm. **D** Relative mRNA levels of *COX1*, *Tfam*, and *ND1*. Data are presented as mean ± SEM. *n* = 4–6. Statistical analysis was performed with the randomized block ANOVA. *P < 0.05; **P < 0.01; ***P < 0.001; *vs*. indicated group.

Treatment with ox-LDL/HG resulted in the decrease of red fluorescence intensity, but the increase of green fluorescence signals, suggesting that the mitochondrial membrane potential of ECs were markedly decreased. Conversely, Rb1 pretreatment attenuated ox-LDL/HG-induced collapse of mitochondrial membrane potential in ECs (Fig. 3A). In addition, administration of Rb1 decreased mitochondrial ROS formation in ECs response to ox-LDL/HG (Fig. 3B). Recent studies have consistently demonstrated that mitochondrial biogenesis impairment plays a pathological role in the occurrence of atherosclerosis [15]. Incubation of ox-LDL/HG disrupted mitochondrial mass with morphological derangements, as indicated by nonyl acridine orange (NAO) staining, while this was normalized by Rb1 (Fig. 3C). In keeping with this, the mRNA levels of mitochondrial DNA-coded genes, such as *ND1*, *COX1*, and mitochondrial transcription factor A (*Tfam*) were diminished in ECs exposed to ox-LDL/HG, but Rb1 pretreatment abrogated these aberrant changes (Fig. 3D), indicative of the ability of Rb1 to regulate mitochondrial biogenesis.

### Rb1 binds directly to Keap1 and promotes its degradation

Molecular docking analysis illustrated the interaction between Rb1 and Keap1 with binding energy of −4.58 KJ/mol and −4.48 KJ/mol at Keap1 site 1 and site 2, respectively. More specifically, five hydrogen bonds are formed between Rb1 and the Tyr525, Ser508, Arg415, Asp573, Val467 in the ligand-binding domain of Keap1 (Fig. S3A). In support, surface plasmon resonance (SPR) and microscale thermophoresis (MST) also revealed the potential interaction of Rb1 with Keap1 (Fig. S3B, C). Of note, the KD values of MST and SPR are inconsistent due to the different sensitivities of these two assays. Although the calculated KD values may differ from our in vitro concentrations of Rb1, this may be caused by the variable sensitivity of MST or SPR. Of importance, the anti-oxidative and mitochondrial protective effects of Rb1 were substantially attenuated in ox-LDL/HG-incubated cells after the residues Tyr525, Ser508, Arg415, Asp573, and Val467 of Keap1 were mutated to alanine, as evidenced by DHE and MitoSOX Red staining (Fig. S4A, B). To determine which sites are more important for Rb1 and Keap1 binding, we transfected ECs with several single-site mutant plasmids, respectively. Results showed that mutations of Tyr525, Arg415, and Val467, rather than Ser508 and Asp573, abolished the pro-proliferation effects of Rb1 on ECs (Fig. S4C), suggesting that Tyr525, Arg415, and Val467 might mediate the potential binding Rb1 to Keap1.

**Fig. 4:**
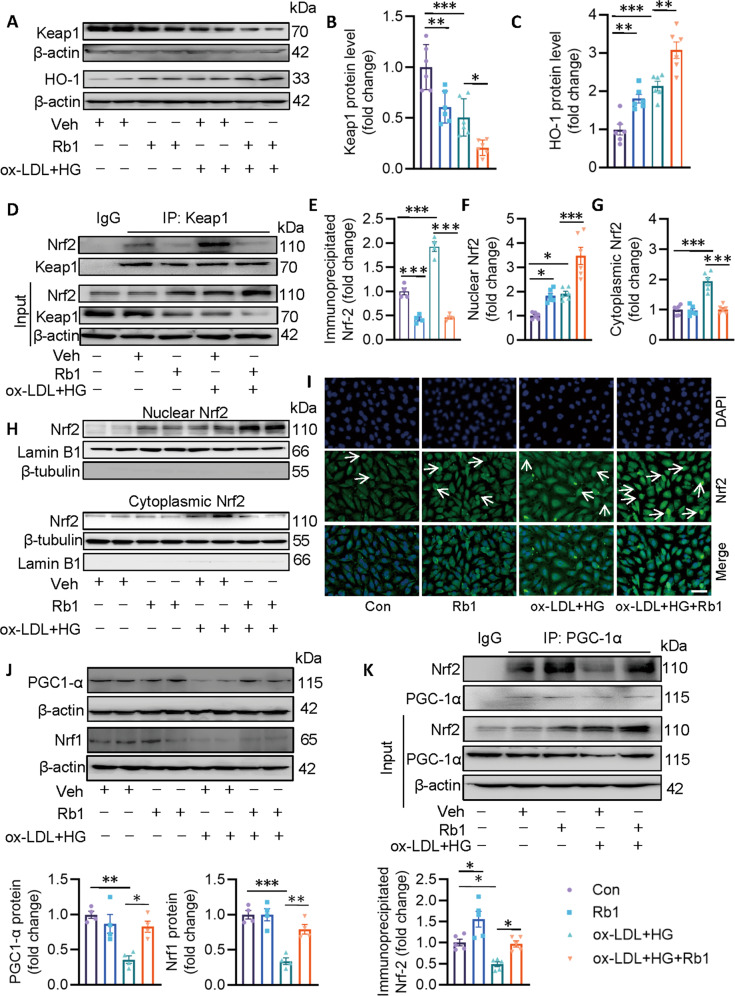
Rb1 promotes the dissociation of Nrf2 from Keap1 and induces the interaction of Nrf2 with PGC-1α. **A**–**C** Representative blots and quantitative analysis of Keap1 and Nrf2. **D**, **E** Cell lysates were immunoprecipitated with an anti-IgG or an anti-Keap1 antibody, then blotted with an anti-Nrf2 antibody. **F**–**H** Representative blots and quantitative analysis of cytoplasmic and nuclear Nrf2. **I** Immunofluorescence staining showing the cellular distribution of Nrf2. Scale bar = 50 μm. **J** Representative blots and quantitative analysis of PGC-1α and Nrf1. **K** Cell lysates were immunoprecipitated with an anti-IgG or an anti-PGC-1α antibody, then blotted with an anti-Nrf2 antibody. Data are presented as mean ± SEM. *n* = 4–6. Statistical analysis was performed with the randomized block ANOVA. *P < 0.05; **P < 0.01; ***P < 0.001; *vs*. indicated group.

We further examined the effect of Rb1 on Keap1 protein expression in ECs with or without ox-LDL/HG. In the presence of ox-LDL/HG, the Keap1 protein was decreased, but the HO-1 protein, a downstream target gene of Nrf2 was increased, possibly an adaptive response to oxidative stress, which was further potentiated by Rb1 (Fig. 4A–C). Interestingly, Rb1 itself promoted the degradation of Keap1 and upregulated the protein expression of HO-1 (Fig. 4A–C), suggesting that Rb1 may be a potential activator of Nrf2 through inhibiting Keap1. We also found that Rb1 decreased the Nrf2/Keap1 interaction in ox-LDL/HG-exposed ECs (Fig. 4D, E). The protein analysis of cytoplasmic and nuclear protein extracts revealed that Rb1 promoted the nuclear accumulation of Nrf2 in ECs and further strengthened the effects of ox-LDL/HG, indicating that Nrf2 is activated upon exposure of ox-LDL/HG (Fig. 4F–H). In line with this, immunofluorescence staining indicated more nuclear staining of Nrf2 in ECs treated with Rb1 when compared with control cells, in the absence or presence of ox-LDL/HG (Fig. 4I).

A number of transcription factors and transcriptional co-activators are involved in mitochondrial biogenesis, including Nrf1, Nrf2, and PGC-1α. In ECs, ox-LDL/HG diminished the protein expressions of PGC-1α and Nrf1, whereas these were rescued by Rb1 pretreatment (Fig. 4J). Importantly, co-immunoprecipitation results showed that ox-LDL/HG impaired the formation of PGC-1α/Nrf2 in ECs, while Rb1 augmented the binding of PGC-1α to Nrf2, hinting that upregulation of Nrf1 and PGC-1α, as well as increased interaction of PGC-1α with Nrf2 are required for Rb1 induction of mitochondrial biogenesis (Fig. 4K). Overall, facilitation of mitochondrial biogenesis via Nrf2 activation functioned as an alternative way for Rb1 to protect mitochondrial integrity from ox-LDL/HG insult.

To further ascertain whether Rb1-induced benefits in ECs were dependent on activation of the Nrf2/PGC-1α pathway, ECs were transfected with Nrf2 siRNA or PGC-1α siRNA. As expected, deficiency of Nrf2 abrogated Rb1-mediated suppression of ROS production, mitochondrial oxidative stress, mitochondrial permeability transition, and mitochondrial mass damage induced by ox-LDL/HG in ECs (Fig. S5A–E). In line with these findings, gene deletion of PGC-1α also abolished the protective effects of Rb1 in ECs (Fig. S6).

### SYVN1 is identified as the E3 ligase of Keap1

**Fig. 5:**
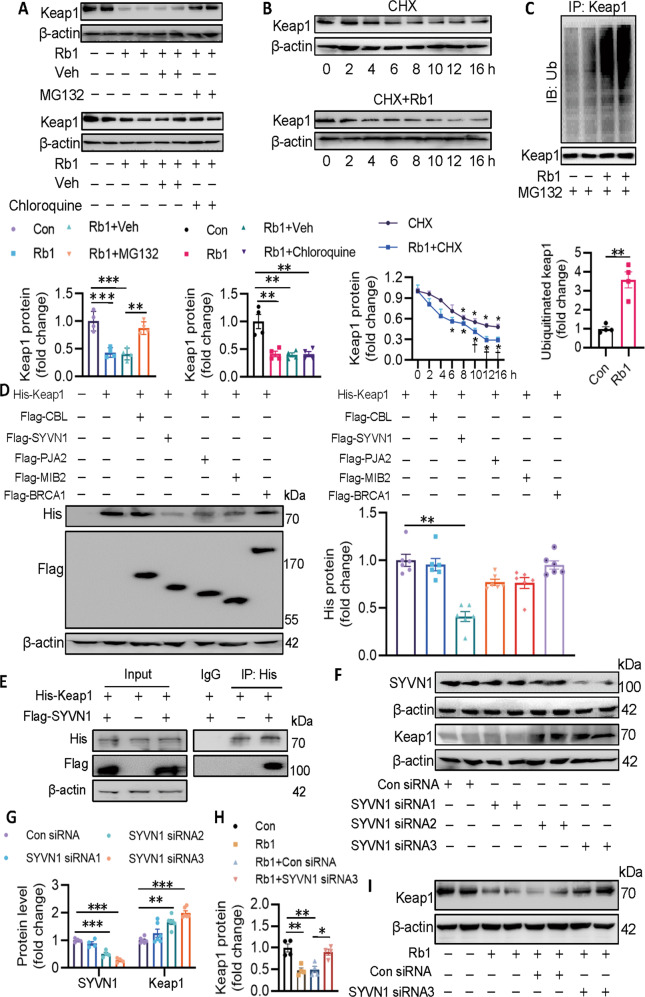
SYVN1 is identified as the E3 ligase of Keap1 and mediates its degradation induced by Rb1. **A** MG132, but not chloroquine, reversed the effects of Rb1 on the protein level of Keap1. **B** Rb1 accelerated the degradation of Keap1 in the presence of CHX. **C** Rb1 accelerated the ubiquitination of Keap1. **D** HEK293T cells were transfected with required plasmids for 48, and cell lysates were immunoblotted to quantify His-Keap1 protein levels. **E** The interaction of Keap1 with SYVN1 was detected by immunoprecipitation. **F**, **G** EA.hy926 ECs were transfected with different siRNAs against SYVN1 for 72 h. Cell lysates were immunoblotted to quantify SYVN1 and Keap1 protein levels. **H**, **I** SYVN1 siRNA3 blocked the suppressive effects of Rb1 on Keap1 protein levels in ECs. Data are presented as mean ± SEM. *n* = 4–6. Statistical analysis was performed with the randomized block ANOVA. *P < 0.05; **P < 0.01; ***P < 0.001; *vs*. indicated group.

We further examined whether Rb1 regulated the protein expression of Keap1 at post-translational modification levels. The degradation of Keap1 by Rb1 was alleviated by the proteasome inhibitor MG-132, but not the autophagy-lysosome inhibitor chloroquine, implying that Rb1 destroyed the stability of Keap1 through proteasomal degradation (Fig. 5A). Half-life experiments showed that Rb1 induced a continuous and rapid degradation from 4 to 16 h in ECs when the protein synthesis was inhibited by cycloheximide (Fig. 5B). Likewise, incubation of Rb1 noticeably promoted the ubiquitination of Keap1 (Fig. 5C).

To explore the potential E3 ligases that lead to the proteasomal degradation of Keap1, we used UbiBrowser, an integrated bioinformatics platform for predicting proteome-wide E3 substrate networks (http://ubibrowser.ncpsb.org). We proposed Keap1 as a substrate, and 20 predicted E3 ligases were identified in this platform (Fig. S7A). These E3 ligases were divided into different families, as shown in the E3 family hierarchical tree (Fig. S7B). Of the 20 predicted E3 ligases, five E3 ligases with the highest confidence scores were filtered and listed (Fig. S7C). To further confirm the E3 ligases of Keap1, five E3 ligases (CBL, SYVN1, PJA2, MIB2, and BRCA1) were overexpressed and the protein expression of Keap1 was correspondingly assessed. Overexpression of SYVN1 resulted in a significant reduction in Keap1 protein, although PJA2 and MIB2 overexpression also reduced Keap1 protein to some extent (Fig. 5D). Of note, the interaction between SYVN1 and Keap1 was validated by co-immunoprecipitation assay (Fig. 5E). Not surprisingly, the SYVN1 siRNA3 with the highest interference efficiency significantly raised the Keap1 protein level (Fig. 5F, G). Consistently, knockdown of SYVN1 eliminated Rb1-induced Keap1 protein downregulation (Fig. 5H, I) and ubiquitination (Fig. S8) in ECs. To sum up, these results provide ample evidence that SYVN1 served as a bona fide E3 ligase of Keap1.

### Rb1 promotes SYVN1-mediated Keap1 ubiquitination at K108, K323, and K551

**Fig. 6:**
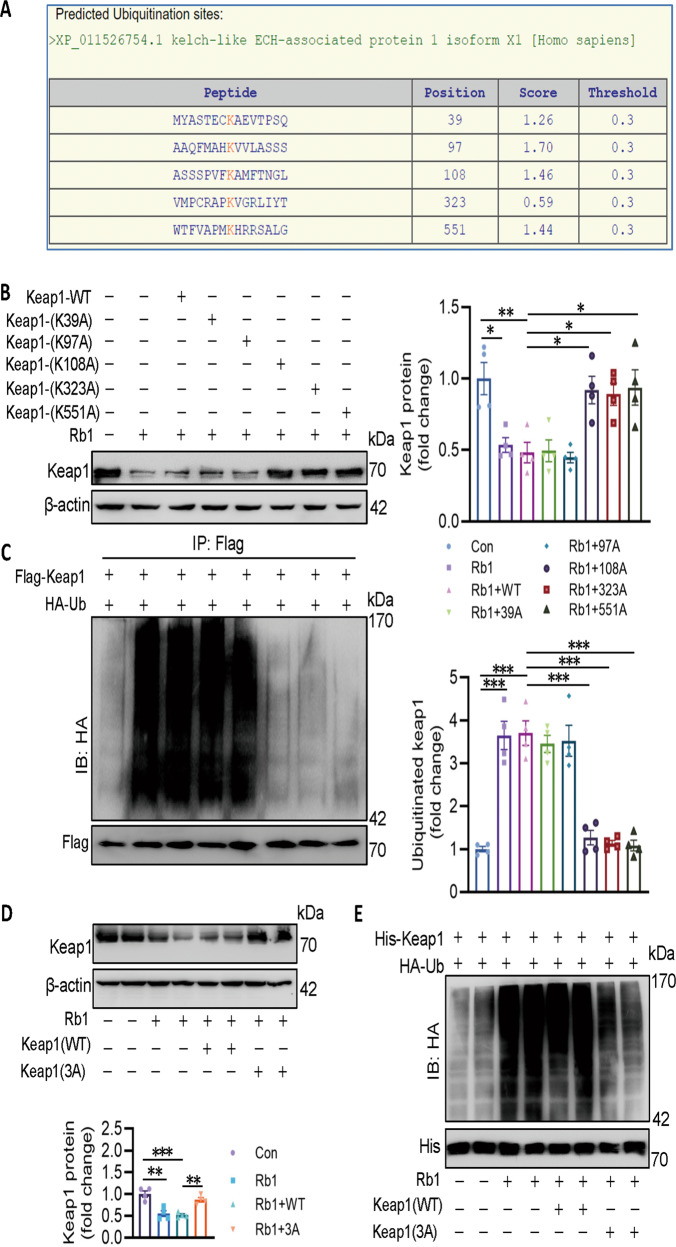
Rb1 promotes SYVN1-mediated keap1 ubiquitination at K108, K323, and K551. **A** The ubiquitination sites of Keap1 predicted by the BDM-PUB database. **B** HEK293T cells were transfected with indicated Keap1 mutation plasmids for 48 h, the cells then treated with Rb1 for 24 h, cell lysates were then immunoblotted to quantify Keap1 protein levels. **C** Effects of different Keap1 mutation plasmids on the ubiquitination levels of Keap1 induced by Rb1 in HEK293T cells. **D** HEK293T cells were transfected with Keap1-(K108A), Keap1-(K323A), and Keap1-(K551A) plasmids for 48 h, the cells then treated with Rb1 for 24 h, cell lysates were then immunoblotted to quantify Keap1 protein levels. **E** Effects of Keap1-(K108A), Keap1-(K323A), and Keap1-(K551A) plasmids on the ubiquitination levels of Keap1 induced by Rb1 in HEK293T cells. Data are presented as mean ± SEM. *n* = 4. Statistical analysis was performed with the randomized block ANOVA. *P < 0.05; **P < 0.01; ***P < 0.001; *vs*. indicated group.

Next, we used the BDM-PUB database (http://bdmpub.biocuckoo.org/prediction.php) to identify the possible ubiquitination sites of Keap1. As seen in Fig. 6A, five lysine residues were predicted to be the ubiquitination sites of Keap1. It turned out that the degradation and ubiquitination of Keap1 by Rb1 was attenuated when K108, K323, and K551 were mutated to alanines (Fig. 6B, C). Moreover, K108, K323, and K551 mutations were resistant to Rb1-induced proteasomal degradation of Keap1 (Fig. 6D, E). Next, we transfected the wild-type or mutant (K3A) plasmids in ECs exposed to ox-LDL/HG. Results showed that the suppressive effects of Rb1 on oxidative stress and mitochondrial ROS overproduction were dramatically reversed by mutant Keap1 (K3A) (Fig. S9A, B). Similarly, mutation of K108, K323, and K551 to alanine alone could also inhibit the protective effects of Rb1 on EC viability in the context of ox-LDL/HG (Fig. S9C). As a consequence, K108, K323, and K551 were identified to be the ubiquitination sites of Keap1 in response to Rb1.

### Rb1 binds directly to p47^phox^ and potentiates its dephosphorylation

Given the inhibitory effect of Rb1 on the p47^phox^ luciferase reporter gene, it is worth investigating whether there is a direct interaction between Rb1 and p47^phox^. The binding of Rb1 to p47^phox^ was calculated by molecular docking analysis, and their binding energy was −8.6 KJ/mol (Fig. S10A). Besides, the direct binding between Rb1 and p47^phox^ was ascertained by the SPR and MST assay (Fig. S10B, C). Collectively, these results suggest that Rb1 directly interacted with p47^phox^, and inhibited its promoter luciferase reporter gene activity. Importantly, Thr4, Gln33, and Arg121 mutations got resistance against Rb1-mediated suppression of EC oxidative stress and mitochondrial function dysfunction in the context of ox-LDL/HG (Fig. S11A, B). Interestingly, mutation of Thr4 and Gln33, but not Arg121, prevented the protective effects of Rb1 against ox-LDL/HG-induced injury in ECs, as evidenced by CCK-8 assay (Fig. S11C), indicating that Thr4 and Gln33 are required for the binding of Rb1 to p47^phox^.

**Fig. 7:**
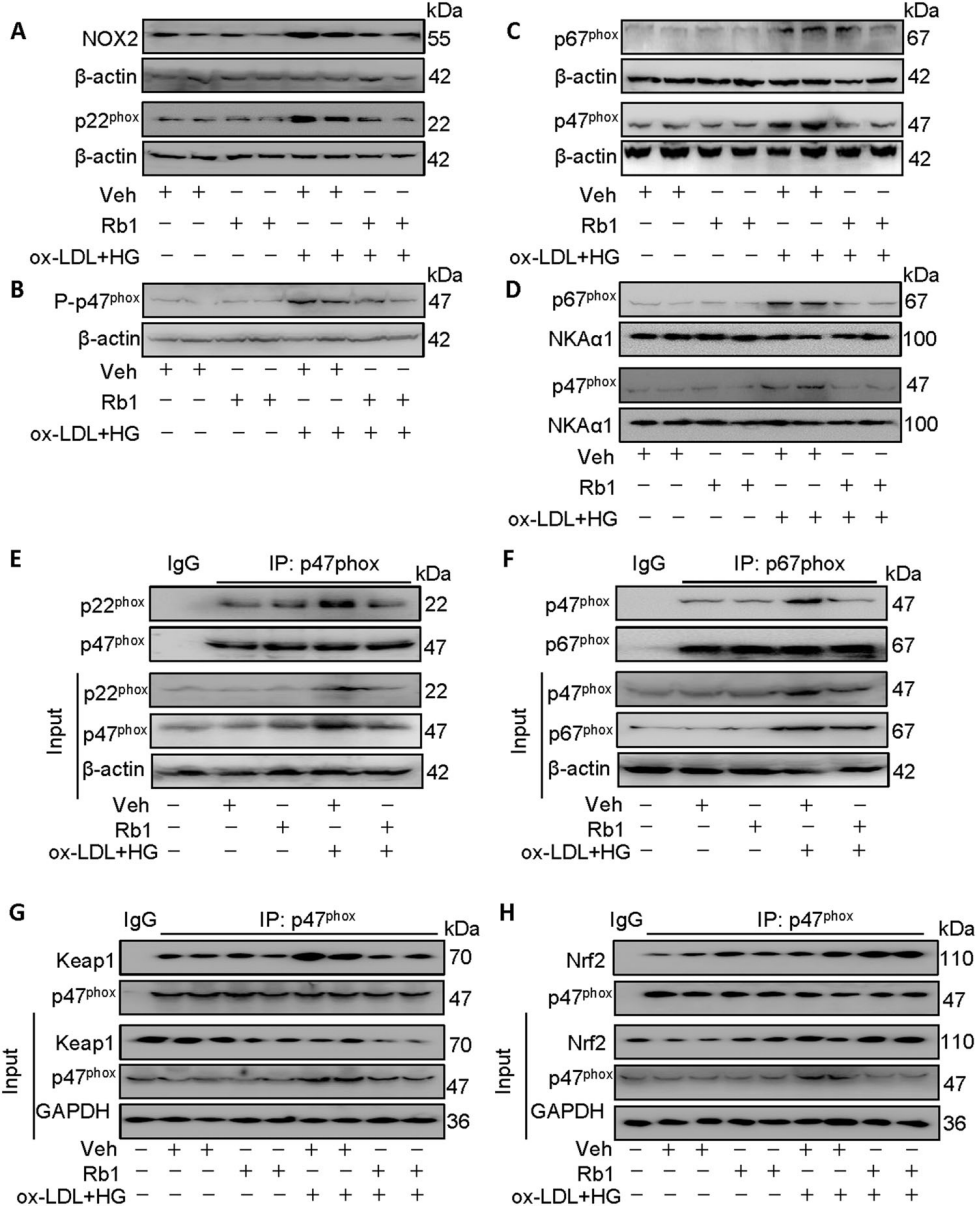
Rb1 potentiates the dephosphorylation and cytoplasmic abundance of p47^phox^, and facilitates the interaction of p47^phox^ and Nrf2. **A** Representative blots of NOX2 and p22^phox^. **B** Representative blots of phosphorylated p47^phox^. **C** Representative blots of total p47^phox^ and p67^phox^. **D** Representative blots of membrane p47^phox^ and p67^phox^. **E** Cell lysates were immunoprecipitated with an anti-IgG or an anti-p47^phox^ antibody, then blotted with an anti-p22^phox^ antibody. **F** Cell lysates were immunoprecipitated with an anti-IgG or an anti-p67^phox^ antibody, then blotted with an anti-p47^phox^ antibody. **G** Cell lysates were immunoprecipitated with an anti-IgG or an anti-p47^phox^ antibody, then blotted with an anti-Keap1 antibody. **H** Cell lysates were immunoprecipitated with an anti-IgG or an anti-p47^phox^ antibody, then blotted with an anti-Nrf2 antibody. Data are presented as mean ± SEM. *n* = 4–6. Statistical analysis was performed with the randomized block ANOVA. *P < 0.05; **P < 0.01; ***P < 0.001; *vs*. indicated group.

The protein expression levels of NOX2 and p22^phox^ were increased in ECs upon exposure of ox-LDL/HG and were reversed by treatment with Rb1 (Fig. 7A and Fig. S12A). Pretreatment with Rb1 reduced the phosphorylation levels of p47^phox^, total p47^phox^ in ox-LDL/HG-stimulated cells, but not total p67^phox^ (Fig. 7B, C and Fig. S12B, C). Of interest, the membrane abundance of p47^phox^ and p67^phox^ was restored to the normal levels in ox-LDL/HG-treated cells after treatment with Rb1 (Fig. 7D and Fig. S12D). In accordance with this, Rb1 pretreatment prevented ox-LDL/HG-induced the formation of both p47^phox^/p22^phox^ complex and p47^phox^/p67^phox^ complex, indicating that Rb1 may be a potential inhibitor of NOX2 (Fig. 7E, F and Fig. S12E, F). Of note, preconditioning of Rb1 disrupted the association of Keap1 with p47^phox^ induced by ox-LDL/HG in ECs, but led to an increase in the interaction of p47^phox^ with Nrf2 in an ox-LDL/HG environment (Fig. 7G, H and Fig. S12G, H), suggesting that the dissociated p47^phox^ from the Keap1/p47^phox^ complex might bind to more Nrf2, resulting in the stability and activation of Nrf2 under oxidative stress state.

### Rb1 protects against atherosclerotic lesions in diabetic ApoE^−/−^ mice

**Fig. 8:**
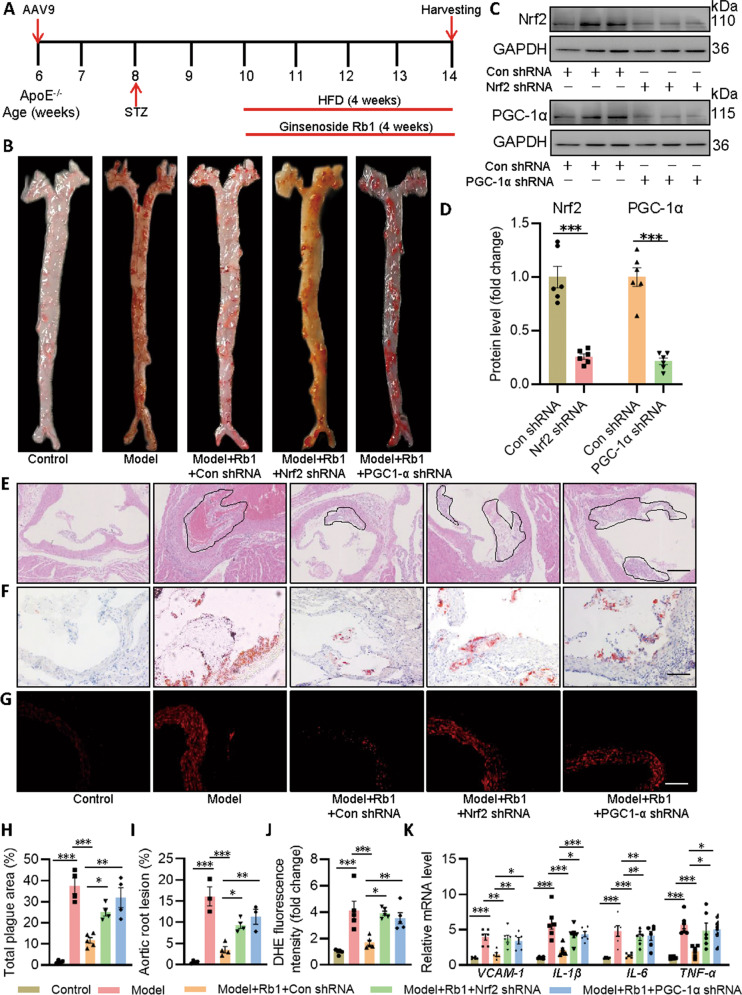
Nrf2 and PGC-1α are required for Rb1 to ameliorate diabetes-accelerated atherosclerosis in mice. **A** Schema showing the experimental procedure in mice. **B**, **H** Lesion areas indicated by Oil O Red staining of the thoracoabdominal aorta. **C**, **D** Representative blots and quantitative analysis of Nrf2 and PGC-1α in primary mouse ECs. **E**, **I** H&E staining and quantification of aortic root sections. Scale bar = 100 μm. **F** Oil O Red staining of aortic root sections. Scale bar = 100 μm. **G**, **J** DHE staining of thoracic aorta. Scale bar = 50 μm. **K** Relative mRNA levels of *VCAM-1, IL-1β*, *IL-6*, and *TNF-α*. Data are presented as mean ± SEM. *n* = 4–6. Statistical analysis was performed with the randomized block ANOVA. *P < 0.05; **P < 0.01; ***P < 0.001; *vs*. indicated group.

To determine the beneficial effects of Rb1 on the development of atherosclerotic lesions in STZ-diabetic ApoE^−/−^ mice, those rodents were treated with Rb1 for 4 weeks together with a HFD was applied to accelerate the process of atherogenesis. Importantly, to further ascertain the involvement of endothelial Nrf2 and/or PGC-1α in mediating the potential benefits of Rb1 in atherosclerosis, these mice were transduced with the recombinant adeno-associated virus 9 (AAV9) carrying Nrf2 shRNA or PGC-1α under the control of Tie1 promoter through tail vein injection (5 × 10^11^ viral particles per animal) (Fig. 8A). As anticipated, diabetic ApoE^−/−^ mice under a HFD diet had lower body weight, higher fasting blood glucose, total cholesterol, triacylglycerol, and LDL when compared with those mice fed by a standard chow (Table S4). Interestingly, these effects were not affected by treatment with Rb1 or specific endothelial knockdown of Nrf2 and PGC-1α (Table S4). We initially examined the total aortic lesion area between the proximal ascending aorta and the bifurcation of the iliac artery by en face Oil O Red staining of aortas. Results showed that the atherosclerotic lesion in the aorta was largely inhibited in Rb1-treated mice in comparison with the diabetic ApoE^−/−^ mice (Fig. 8B). However, Rb1 failed to attenuate the formation of atherosclerotic plaques when the Nrf2 or PGC-1α was absent in the endothelium (Fig. 8B–D). Similar results were detected by H&E staining and Oil O Red staining in aortic root (Fig. 8E, F, I). DHE staining of aortic sections revealed that the production of superoxide anions was increased in mice with diabetic atherosclerosis, and this effect was prevented by Rb1 treatment dependent on the presence of Nrf2 and PGC-1α in the endothelium (Fig. 8G, J). Likewise, the contents of pro-inflammatory factors, such as *VCAM-1, IL-1β*, *IL-6*, and *TNF-α*, were obviously elevated in the endothelium from diabetic ApoE^−/−^ mice, which were erased after Rb1 treatment (Fig. 8K). Specific deficiency of Nrf2 or PGC-1α in the endothelium abolished the anti-inflammatory effects of Rb1 in the aortas from model mice (Fig. 8K). These results indicate that exogenous Rb1 attenuates diabetes-aggravated atherosclerosis, likely by maintaining redox balance and mitochondrial homeostasis via the Nrf2/PGC-1α pathway.

## Discussion

It is well accepted that modifications of the Keap1 cysteines lead directly to the dissociation of the Keap1/Nrf2 complex, subsequent Nrf2 nuclear accumulation and activation [16]. Recently, p47^phox^ has been found to bind to Nrf2 in cytoplasm, which is necessary for Nrf2 deubiquitination and nuclear translocation [10]. To this end, we selected the potential compounds that can simultaneously regulate Keap1 and p47^phox^ to activate Nrf2, thereby achieving the goal (Nrf2) of killing two birds (Keap1 and p47^phox^) with one stone (Rb1). In this study, we revealed that Rb1 promoted the interaction of Keap1 with an E3 ligase SYVN1 at specific lysine residues (K108, K323, and K551), leading to Keap1 degradation through the ubiquitination proteasome-dependent pathway, Nrf2 nuclear accumulation and activation. Of interest, mutations of lysines 108, 323, and 551 were sufficient to prevent the effects of Rb1 on the degradation and ubiquitination of Keap1. In particular, mutation of one of these sites alone, or mutation of all three sites simultaneously, seemed to completely abolish the effect of Rb1 on Keap1 degradation and ubiquitination. These results show that the three lysines (108, 323, and 551) within Keap1 protein may be necessary for Keap1 to form a polyubiquitination site. In the process of Keap1 ubiquitination, it is unknown whether these three amino acids are close enough to each other in space to form a special spatial structure favorable for Keap1 ubiquitination, and this awaits further confirmation by appropriate techniques, such as co-crystal culture of proteins and small molecules. By binding to p47^phox^, Rb1 induced p47^phox^ dephosphorylation and cytoplasmic retention, resulting in the complex formation of p47^phox^/Nrf2 which promoted Nrf2 nuclear accumulation and activation. On the one hand, the nuclear translocation of Nrf2 caused the upregulation of HO-1, a classical anti-oxidative enzyme to combat oxidative stress. On the other hand, the complex formation of Nrf2/PGC-1α in the nucleus provoked the process of mitochondrial biogenesis. This study demonstrated that synergistic regulation of Keap1 and p47^phox^ by Rb1 contributes to Nrf2 activation in ECs, thus protecting against endothelial dysfunction and diabetes-accelerated atherosclerosis (Fig. S13).

Studies have demonstrated that increased advanced glycation end product generation, protein kinase C activation, increased polyol pathway flux, inflammation, endoplasmic reticulum stress, mitochondrial dysfunction, cell apoptosis, and endothelial-mesenchymal transition are critical contributors to diabetic vascular complications, including atherosclerosis. These pathological mechanisms are associated with hyperglycemia-triggered oxidative stress and ROS formation [2, 17]. In this study, we found that STZ-induced ApoE^−/−^ mice fed a HFD exhibited obvious atherosclerotic plaques compared with control mice, along with increases in superoxide production and pro-inflammatory factors in aorta. In keeping with this, the decreased cell viability, increased cell apoptosis, oxidative burst, endothelial-mesenchymal transition, and inflammation were observed in ox-LDL/HG-induced ECs. These changes were effectively ameliorated by Rb1 treatment, indicating that Rb1 showed powerful antioxidant and anti-inflammatory properties, as supported by a wide range of studies [18–21]. In light of the strong antioxidant ability of Rb1, it is not unexpected to see that Rb1 treatment decreased ROS generation in ECs cultured with ox-LDL/HG. Overall, we demonstrated that blockade of ROS overproduction by Rb1 could prevent diabetes-accelerated atherosclerosis.

Recently, mitochondria dysfunction is emerging as a common pathology of atherosclerosis, involving mitochondria ROS formation, mitochondrial membrane potential collapse, and impaired mitochondria mass [22–24]. In this study, we found that ox-LDL/HG promoted mitochondrial membrane potential turnover, enhanced ROS overproduction in mitochondria, decreased mitochondrial biogenesis and mass, such observations that were largely reversed by Rb1 treatment. Similar to this, Rb1 promoted mitochondrial biogenesis-related gene levels, increased Nrf1 and PGC-1α protein levels, in conjunction with a more interaction of Nrf2 with PGC-1α. Notably, deficiency of either Nrf2 or PGC-1α abolished the effects of Rb1 on oxidative stress and mitochondrial function in ox-LDL-HG-incubated ECs. In line with this, specific deletion of endothelial Nrf2 and PGC-1α attenuated the protective effects of Rb1 on the development of arteriosclerotic plaque and aortic oxidative stress/inflammation in mice with diabetes-accelerated atherosclerosis. Thereby, our results sustain the benefits of Nrf2/PGC-1α-mediated redox homeostasis and mitochondrial biogenesis in the context of diabetic atherosclerosis. Of note, Rb1 required both Nrf2 and PGC-1α to ameliorate diabetes-accelerated atherosclerosis in rodents without affecting metabolic parameters, suggesting that the benefit of Rb1 in diabetic atherosclerosis was independent of alterations in glucose and lipid metabolism. One of the limitations of this study is that in vivo studies are limited to histological and mRNA levels. Thereby, to better understand the correlation of Rb1, Nrf2, and PGC-1α in this experimental model, it is important to show whether different experimental groups significantly altered other cardiovascular parameters in vivo, such as cardiac function, foaming of vascular smooth muscle cells, and recruitment of macrophages, which merits further studies.

In summary, our findings enrich the pharmacological indications of Rb1 and provide definitive evidence for the diverse functions of Rb1 as a potential food additive. Future studies are highly required to develop Rb1 as an attractive compound for pharmacological intervention of diabetes-accelerated atherosclerosis.

## Supplementary information

aj-checklist

Agreement from all authors including additions

Supplementary information

Supplementary Figure 1

Supplementary Figure 2

Supplementary Figure 3

Supplementary Figure 4

Supplementary Figure 5

Supplementary Figure 6

Supplementary Figure 7

Supplementary Figure 8

Supplementary Figure 9

Supplementary Figure 10

Supplementary Figure 11

Supplementary Figure 12

Supplementary Figure 13

Supplementary Table 1

Supplementary Table 2

Supplementary Table 3

Supplementary Table 4

Supplementary file of western blot.

## Data Availability

All data generated or analyzed during this study are included in this article.
