# Supplementary material for: A dual Keap1 and p47phox inhibitor Ginsenoside Rb1 ameliorates high glucose/ox-LDL-induced endothelial cell injury and atherosclerosis
Source: Cell Death Dis. 2022 Sep 26;13(9):824. doi: 10.1038/s41419-022-05274-x (PMC9512801; doi:10.1038/s41419-022-05274-x)
Supplement: Supplementary file 3 — Supplementary information [file 41419_2022_5274_MOESM3_ESM.docx]

**Supplementary Figures**

**Supplementary Fig. S1. Effects of different compounds on Keap1 and p47^phox^ luciferase reporter activity.** (**A**) Construction of Keap1 luciferase reporter gene plasmid. (**B**) Construction of p47^phox^ luciferase reporter gene plasmid. (**C**) EA.hy926 ECs were transfected with Keap1 luciferase reporter gene plasmid (0.5 μg) for 24 h, and then treated with 379 compounds (10 μM) for 24 h. Cell lysates were subjected to luciferase reporter assay. Heatmap showing the effects of various compounds on the Keap1 luciferase reporter gene activity (upper), and top ten compounds that inhibited Keap1 luciferase reporter gene activity were shown in lower panel. (**D**) EA.hy926 ECs were transfected with p47^phox^ luciferase reporter gene plasmid (0.5 μg) for 24 h, and then treated with 379 compounds (10 μM) for 24 h. Cell lysates were subjected to luciferase reporter assay. Heatmap showing the effects of various compounds on the p47^phox^ luciferase reporter gene activity (upper), and top ten compounds that inhibited p47^phox^ luciferase reporter gene activity were shown in lower panel. (**E**) Venn diagram showed that Rb1 was selected as a dual Keap1 and p47^phox^ inhibitor.

**Supplementary Fig. S2. Effects of different Rb1 treatment conditions on ox-LDL/HG-induced EC damage.** (**A,D**) EA.hy926 ECs were pretreated with Rb1 (10 μM) for 30 min before ox-LDL/HG for 24 h. Cell viability measured by CCK-8 assay, and oxidative state in ECs was monitored by DHE staining. (**B,E**) EA.hy926 ECs were challenged by ox-LDL/HG for 3 h, and then treated with Rb1 (10 μM) for 21 h. Cell viability measured by CCK-8 assay, and oxidative state in ECs was monitored by DHE staining. (**C,F**) EA.hy926 ECs were challenged by ox-LDL/HG for 12 h, and then treated with Rb1 (10 μM) for 12 h. Cell viability measured by CCK-8 assay, and oxidative state in ECs was monitored by DHE staining. Scale bar = 200 μm. Data are presented as mean ± SEM. n = 4-6. Statistical analysis was performed with the randomized block ANOVA. **P*<0.05; ***P*<0.01; ****P*<0.001; *vs*. indicated group.

**Supplementary Fig. S3. Rb1 binds directly to Keap1**. (**A**) Molecular docking of Rb1 to Keap1. (**B**) MST showing the interaction of Rb1 with Keap1. (**C**) SPR showing the interaction of Rb1 with Keap1.

**Supplementary Fig. S4. Keap1 binding site mutant plasmids abolished the benefits of Rb1 in EC protection**. EA.hy926 ECs were transfected with five Keap1 mutant plasmids (Tyr525, Ser508, Arg415, Asp573 and Val467 were mutated to Alanine, respectively) for 24 h, and then treated with Rb1 (10 μM) for 30 min before exposure of ox-LDL/HG for additional 24 h. (**A**) Representative images of DHE staining. Scale bar = 200 μm. (**B**) Mitochondrial ROS determined by MitoSOX Red. (**C**) Effects of Keap1 mutant plasmids (Tyr525, Ser508, Arg415, Asp573 and Val467) alone on the EC viability in Rb1-treated cells in the presence of ox-LDL/HG. Scale bar = 50 μm. n = 4-6. **P*<0.05; ***P*<0.01; ****P*<0.001; *vs*. indicated group.

**Supplementary Fig. S5. Deletion of Nrf2 weakened the protective effects of Rb1 on ox-LDL/HG-induced EC injury**. EA.hy926 ECs were transfected with con siRNA and Nrf2 siRNA for 24 h, and then treated with Rb1 (10 μM) for 30 min before exposure of ox-LDL/HG for additional 24 h. (**A**) Nrf2 interference efficiency detection by immunoblot. (**B**) Representative images of DHE staining. Scale bar = 200 μm. (**C**) Mitochondrial ROS determined by MitoSOX Red. Scale bar = 50 μm. (**D**) Cell membrane potential determined by JC-1 staining. Scale bar = 50 μm. (**E**) Mitochondrial mass determined by NAO staining. Scale bar = 50 μm. Data are presented as mean ± SEM. n = 4. Statistical analysis was performed with the randomized block ANOVA. **P*<0.05; ***P*<0.01; ****P*<0.001; *vs*. indicated group.

**Supplementary Fig. S6. Ablation of PGC-1α erased the protective effects of Rb1 on ox-LDL/HG-induced EC injury**. EA.hy926 ECs were transfected with con siRNA and PGC-1α siRNA for 24 h, and and then treated with Rb1 (10 μM) for 30 min before exposure of ox-LDL/HG for additional 24 h. (**A**) PGC-1α interference efficiency detection by immunoblot. (**B**) Representative images of DHE staining. Scale bar = 200 μm. (**C**) Mitochondrial ROS determined by MitoSOX Red. Scale bar = 50 μm. (**D**) Cell membrane potential determined by JC-1 staining. Scale bar = 50 μm. (**E**) Mitochondrial mass determined by NAO staining. Scale bar = 50 μm. Data are presented as mean ± SEM. n = 4. Statistical analysis was performed with the randomized block ANOVA. **P*<0.05; ***P*<0.01; ****P*<0.001; *vs*. indicated group.

**Supplementary Fig. S7. Predicted E3 ligases that regulate Keap1 ubiquitination**. (**A**) A network view of E3-Keap1 interactions. (**B**) The E3 hierarchical tree for Keap1. (**C**) The five predicted E3 ligases with high confidence.

**Supplementary Fig. S8. Deficiency of SYVN1 prevented the effects of Rb1 on** **Keap1 ubiquitination.** EA.hy926 ECs were transfected with control siRNA or SYVN1 siRNA for 6 h, and co-transfected with His-Keap1 plasmid and HA-Ub plasmid for 24 h, and treated with Rb1 for 24 h. (**A, B**) Representative blots and quantitative analysis of ubiquitinated Keap1. Data are presented as mean ± SEM. n = 4. Statistical analysis was performed with the randomized block ANOVA. **P*<0.05; ***P*<0.01; ****P*<0.001; *vs*. indicated group.

**Supplementary Fig. S9. Keap1 ubiquitination site mutant plasmids abolished the roles of Rb1 in EC protection**. EA.hy926 ECs were transfected with three Keap1 mutant plasmids (K108, K323 and K551 were mutated to Alanine, respectively) for 24 h, and then treated with Rb1 (10 μM) for 30 min before exposure of ox-LDL/HG for additional 24 h. (**A**) Representative images of DHE staining. Scale bar = 200 μm. (**B**) Mitochondrial ROS determined by MitoSOX Red. (**C**) Effects of Keap1 mutant plasmids (K108, K323 and K551) alone on the EC viability in Rb1-treated cells in the presence of ox-LDL/HG. Scale bar = 50 μm. n = 4-6. **P*<0.05; ***P*<0.01; ****P*<0.001; *vs*. indicated group.

**Supplementary Fig. S10. Rb1 binds directly to p47^phox^**. (**A**) Molecular docking of Rb1 to p47^phox^. (**B**) MST showing the interaction of Rb1 with p47^phox^. (**C**) SPR showing the interaction of Rb1 with p47^phox^.

**Supplementary Fig. S11.** **p47^phox^ binding site mutant plasmids abolished the benefits of Rb1 in EC protection**. EA.hy926 ECs were transfected with three p47^phox^ mutant plasmids (Thr4, Gln33 and Arg121 were mutated to Alanine, respectively) for 24 h, and then treated with Rb1 (10 μM) for 30 min before exposure of ox-LDL/HG for additional 24 h. (**A**) Representative images of DHE staining. Scale bar = 200 μm. (**B**) Mitochondrial ROS determined by MitoSOX Red. (**C**) Effects of p47^phox^ mutant plasmids (Thr4, Gln33 and Arg121) alone on the EC viability in Rb1-treated cells in the presence of ox-LDL/HG. Scale bar = 50 μm. n = 4-6. **P*<0.05; ***P*<0.01; ****P*<0.001; *vs*. indicated group.

**Supplementary Fig. S12. Quantitative analysis of original blots from figure 7**. (**A**) Quantitative analysis of NOX2 and p22^phox^. (**B**) Quantitative analysis of phosphorylated p47^phox^. (**C**) Quantitative analysis of total p47^phox^ and p67^phox^. (**D**) Quantitative analysis of membrane p47^phox^ and p67^phox^. (**E**) Quantitative analysis of immunoprecipitated p22^phox^. (**F**) Quantitative analysis of immunoprecipitated p47^phox^. (**G**) Quantitative analysis of immunoprecipitated Keap1. (**H**) Quantitative analysis of immunoprecipitated Nrf2. Data are presented as mean ±SEM. n = 4-6. Statistical analysis was performed with the randomized block ANOVA. **P*<0.05; ***P*<0.01; ****P*<0.001; *vs*. indicated group.

**Supplementary Fig. S13. Sketch diagram showing the proposed mechanisms of Rb1 in attenuating diabetes-accelerated atherosclerosis.** On the one hand, Rb1 promoted the interaction of Keap1 with an E3 ligase SYVN1 at specific lysine residues (K108, K323 and K551), leading to Keap1 degradation through the ubiquitination proteasome-dependent pathway, Nrf2 nuclear accumulation and activation. On the other hand, Rb1 induced p47^phox^ dephosphorylation and cytoplasmic retention, resulting in the complex formation of p47^phox^/Nrf2, Nrf2 nuclear accumulation and activation. The nuclear translocation of Nrf2 caused the upregulations of HO-1, a classical anti-oxidative enzyme to combat oxidative stress, and the complex formation of Nrf2/PGC-1α in nucleus provoked the process of mitochondrial biogenesis, thereby protecting against endothelial dysfunction and diabetes-accelerated atherosclerosis.

**Supplementary Tables**

**Supplementary Table 1. The information of the primary and secondary antibodies used in this study.**

**Supplementary Table 2. Compound information and serial numbers in this study** **and their inhibition rate (%) of Keap1 and p47^phox^ luciferase reporter gene activities.**

**Supplementary Table 3. Primers sequences for real time PCR.**

**Supplementary Table 4. Metabolic parameters of nondiabetic and diabetic ApoE^−/−^ mice.**
