# Supplementary figures and images for: A dual Keap1 and p47phox inhibitor Ginsenoside Rb1 ameliorates high glucose/ox-LDL-induced endothelial cell injury and atherosclerosis
Source: Cell Death Dis. 2022 Sep 26;13(9):824. doi: 10.1038/s41419-022-05274-x (PMC9512801; doi:10.1038/s41419-022-05274-x)

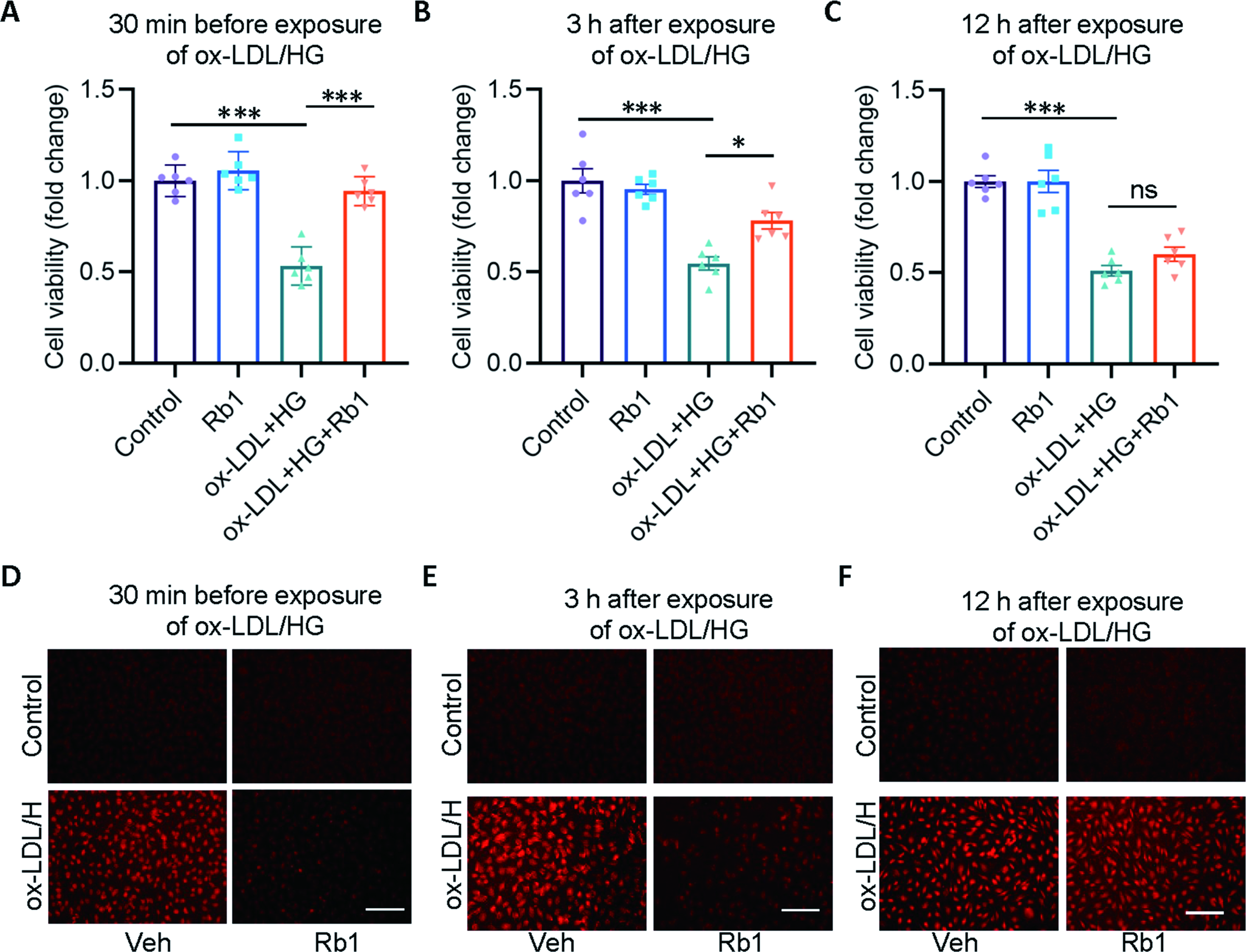

Supplement: Supplementary file 5 — Supplementary Figure 2 [file 41419_2022_5274_MOESM5_ESM.tif]

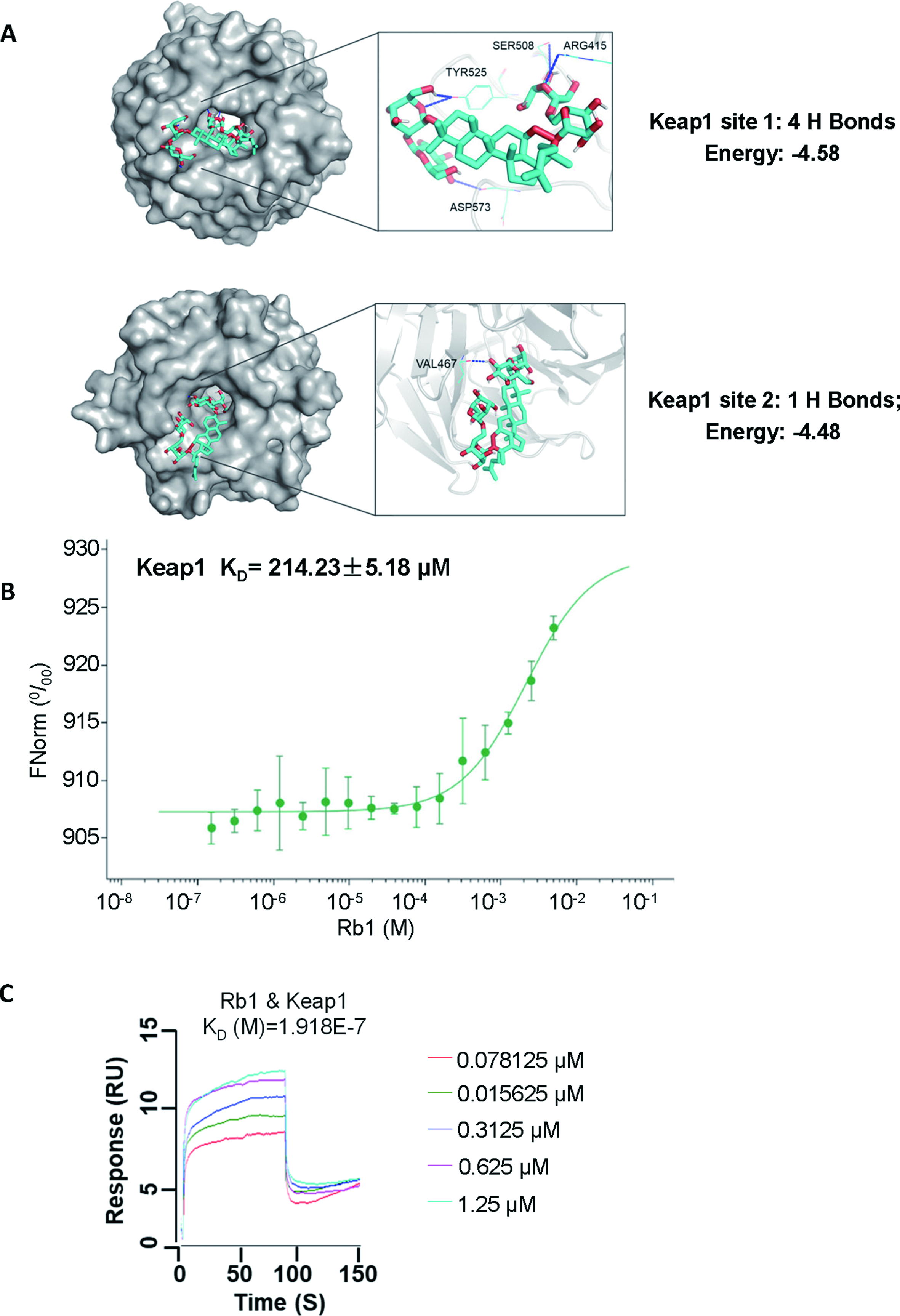

Supplement: Supplementary file 6 — Supplementary Figure 3 [file 41419_2022_5274_MOESM6_ESM.tif]

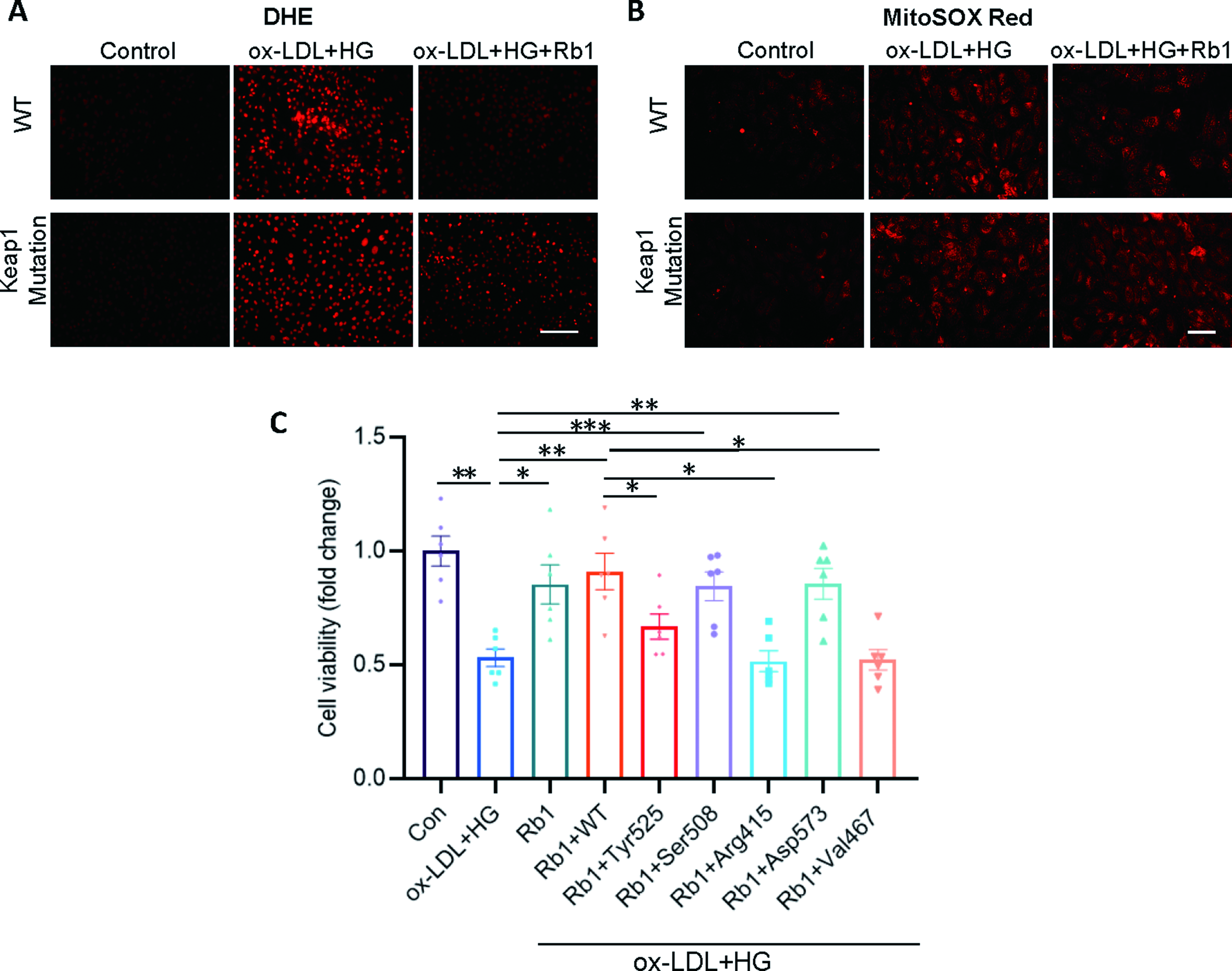

Supplement: Supplementary file 7 — Supplementary Figure 4 [file 41419_2022_5274_MOESM7_ESM.tif]

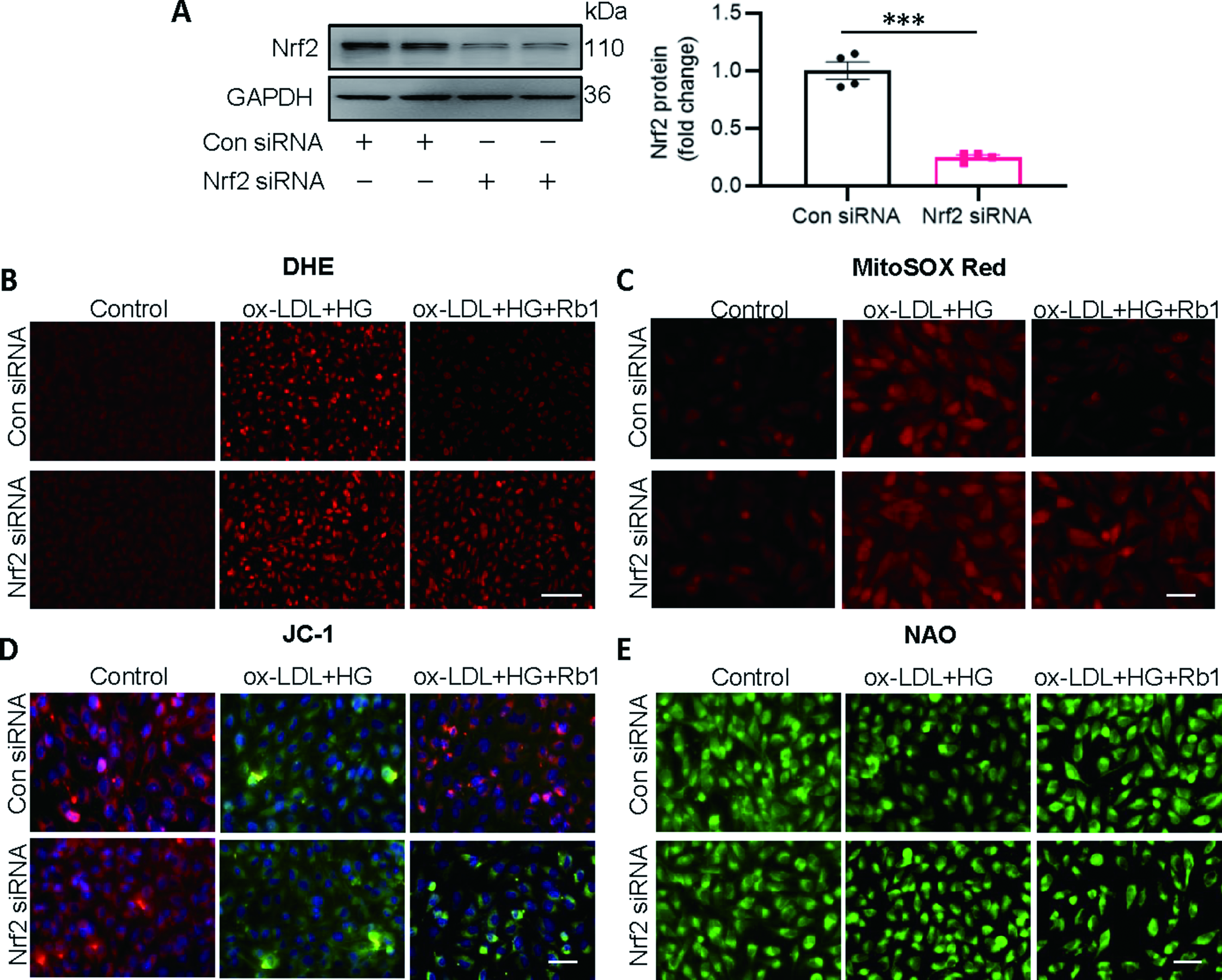

Supplement: Supplementary file 8 — Supplementary Figure 5 [file 41419_2022_5274_MOESM8_ESM.tif]

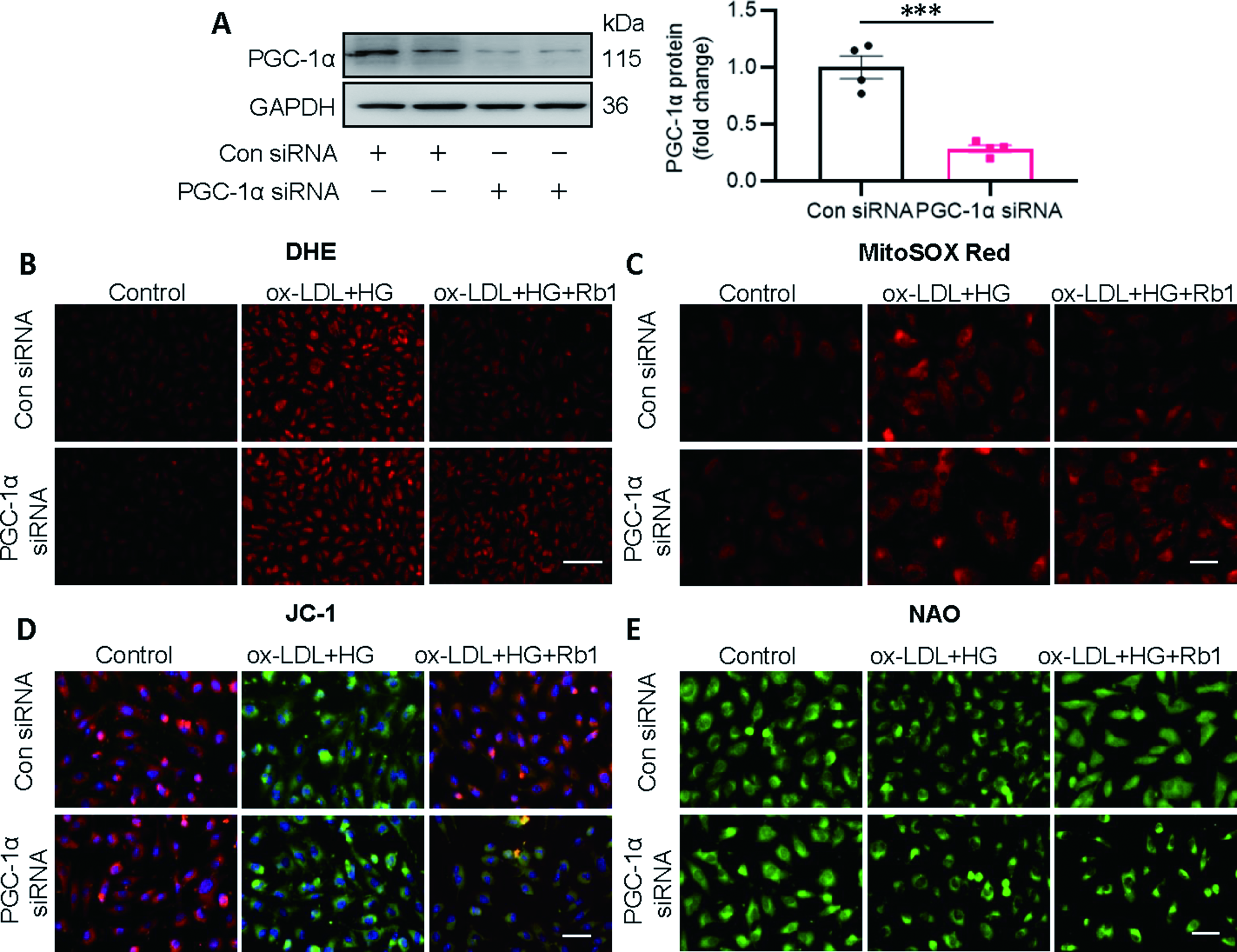

Supplement: Supplementary file 9 — Supplementary Figure 6 [file 41419_2022_5274_MOESM9_ESM.tif]

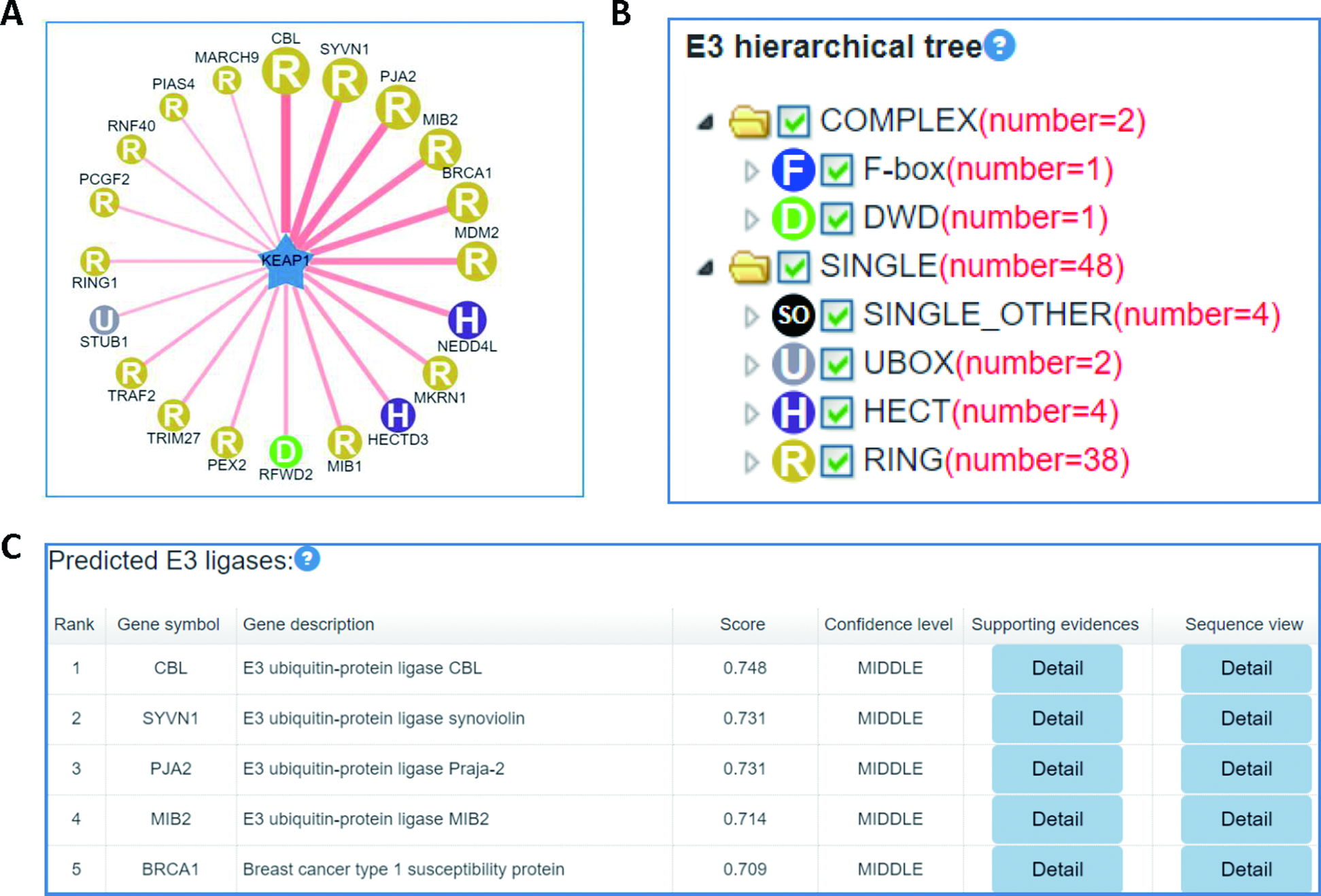

Supplement: Supplementary file 10 — Supplementary Figure 7 [file 41419_2022_5274_MOESM10_ESM.tif]

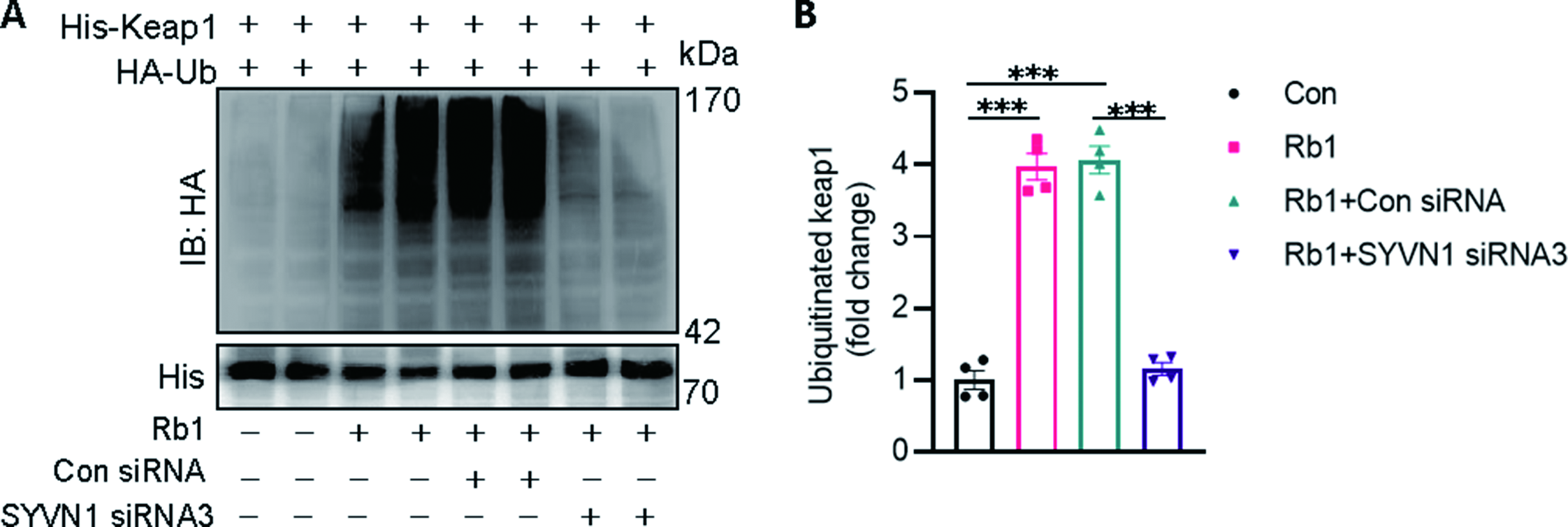

Supplement: Supplementary file 11 — Supplementary Figure 8 [file 41419_2022_5274_MOESM11_ESM.tif]

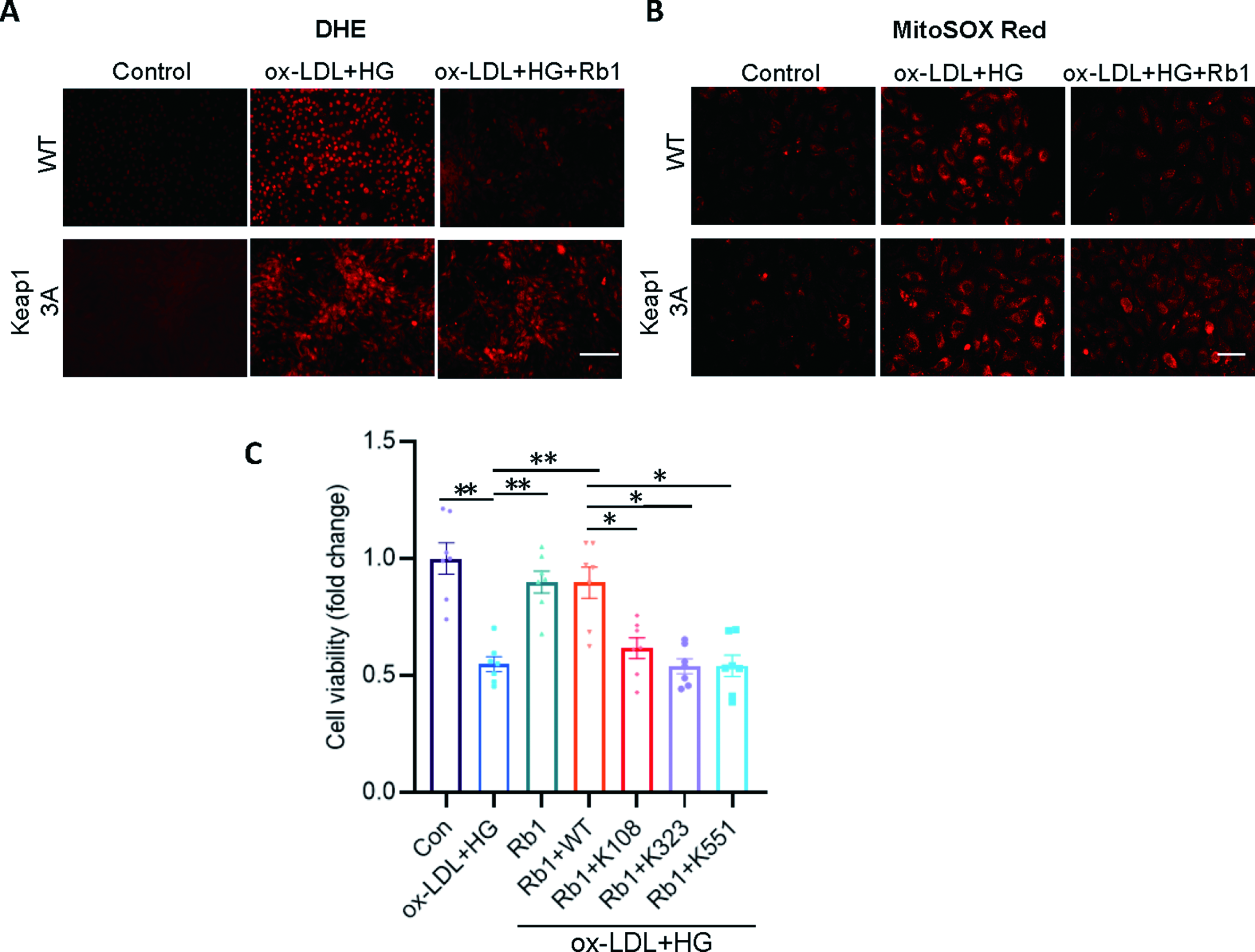

Supplement: Supplementary file 12 — Supplementary Figure 9 [file 41419_2022_5274_MOESM12_ESM.tif]

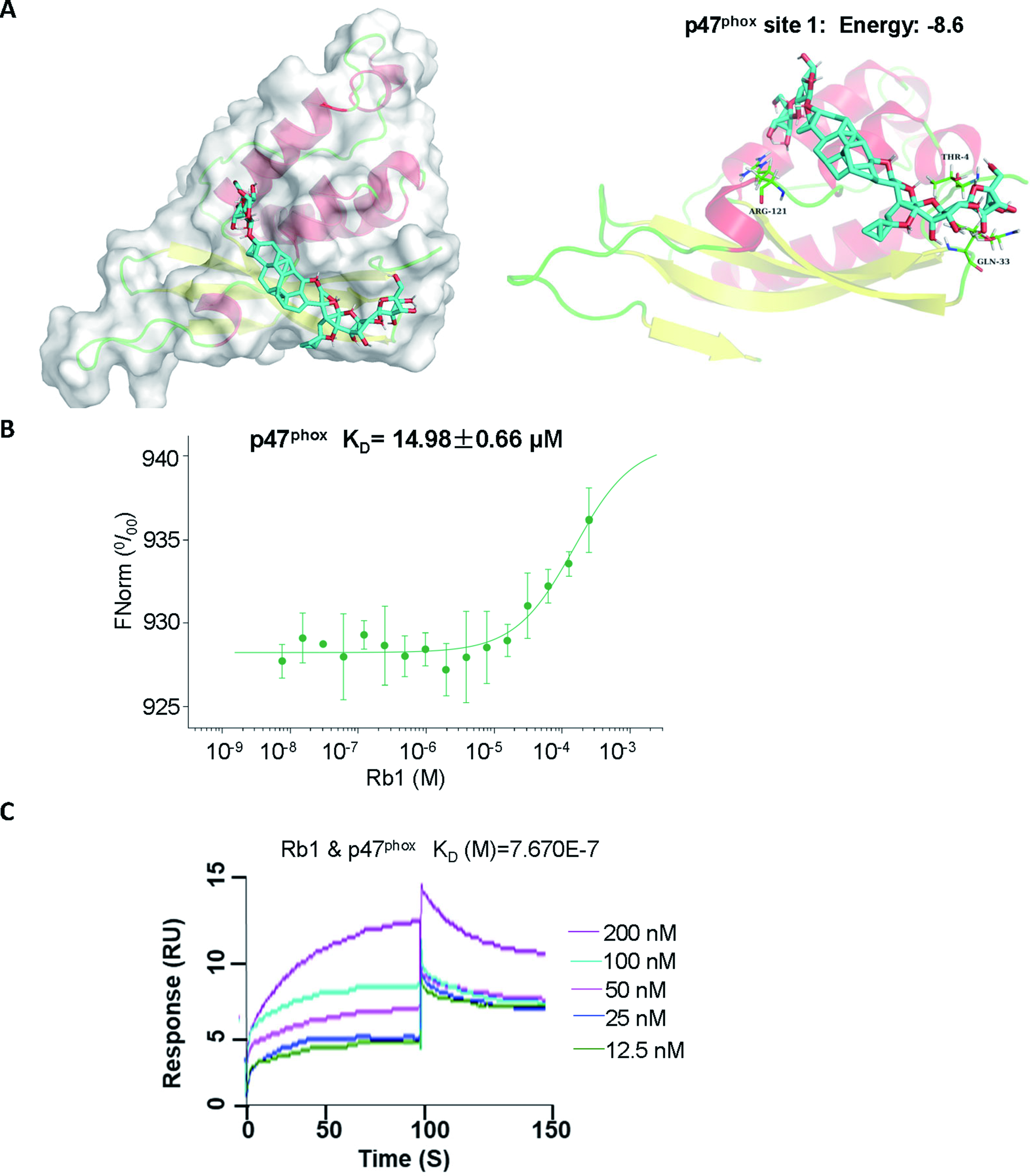

Supplement: Supplementary file 13 — Supplementary Figure 10 [file 41419_2022_5274_MOESM13_ESM.tif]

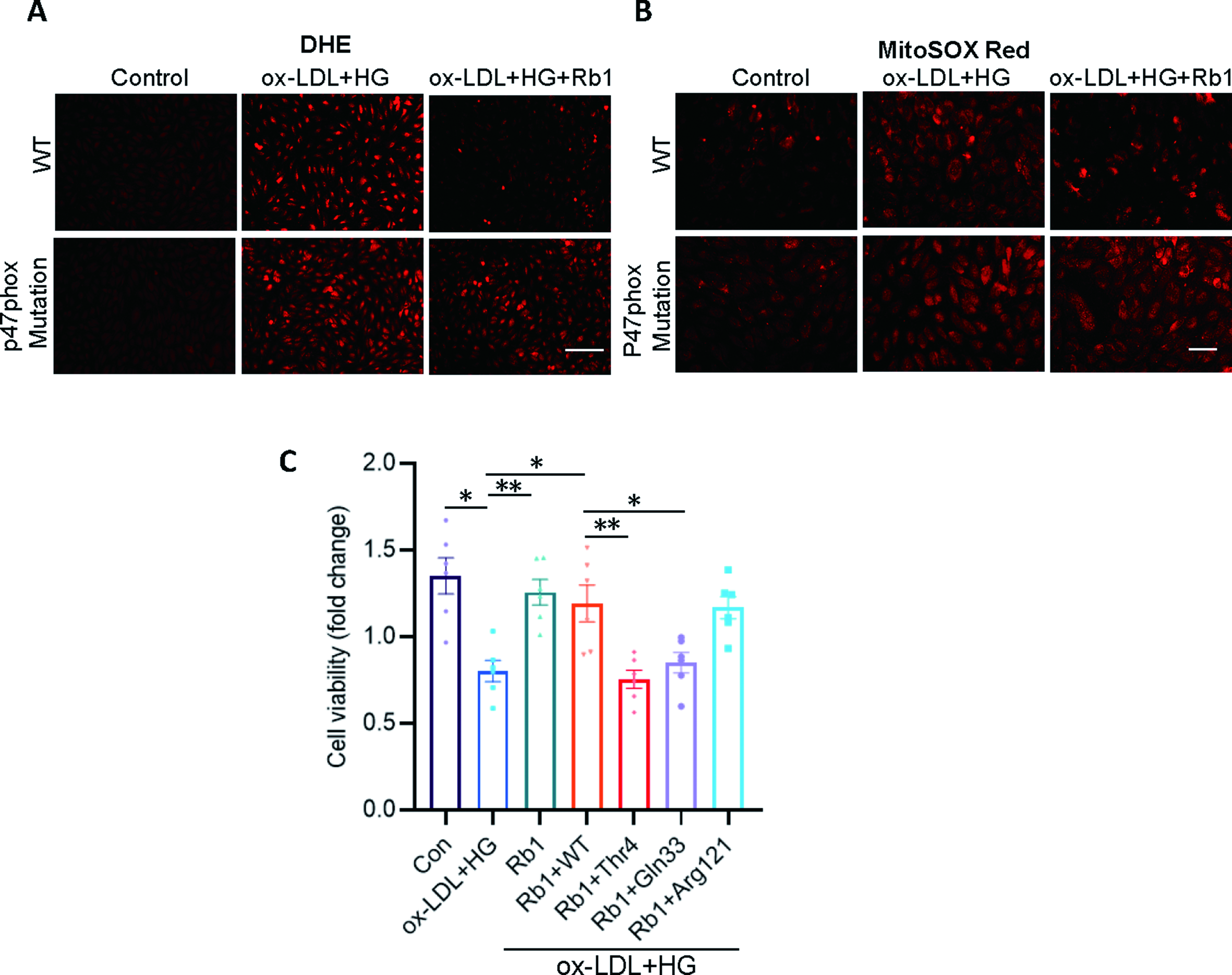

Supplement: Supplementary file 14 — Supplementary Figure 11 [file 41419_2022_5274_MOESM14_ESM.tif]

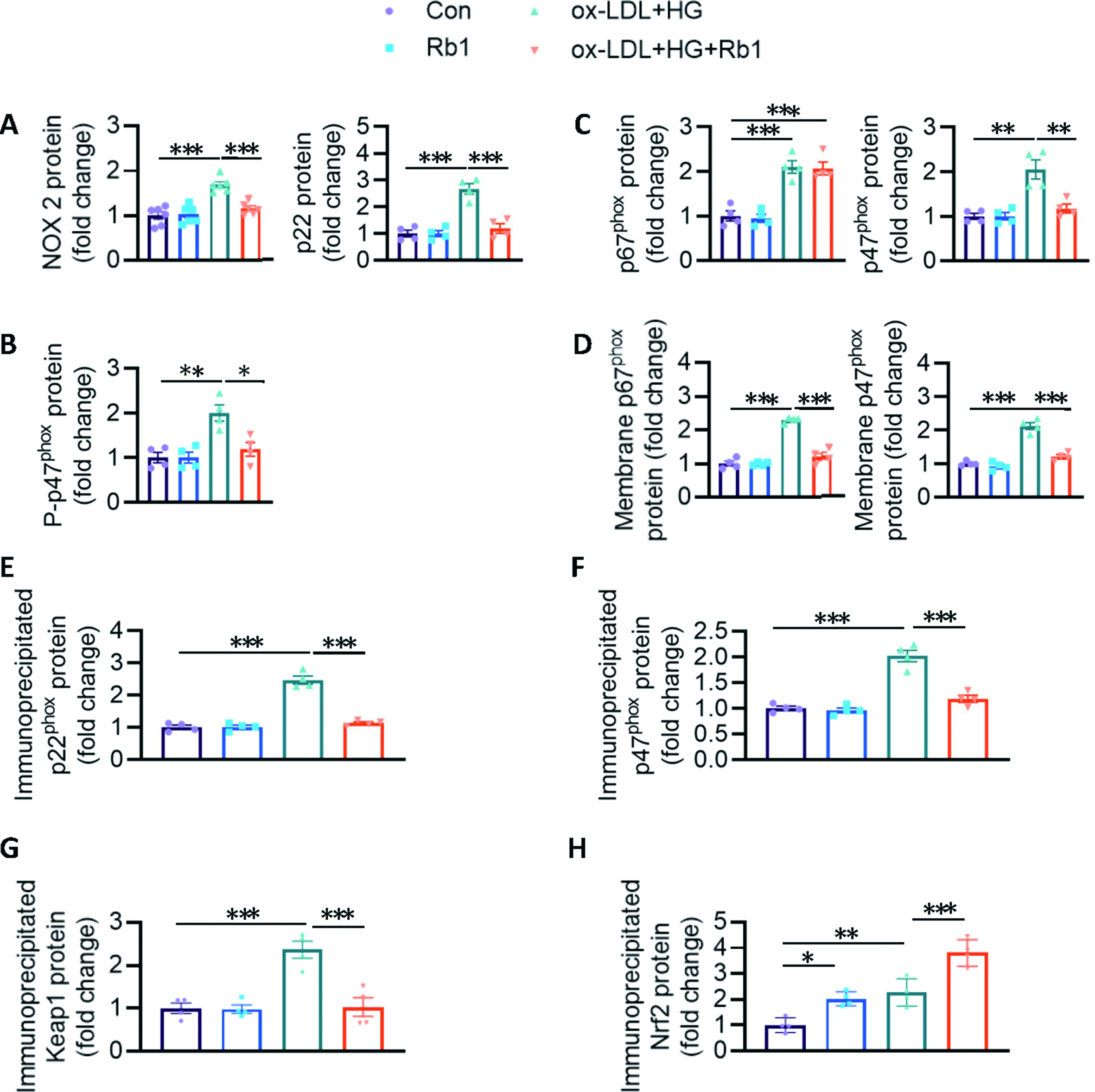

Supplement: Supplementary file 15 — Supplementary Figure 12 [file 41419_2022_5274_MOESM15_ESM.tif]

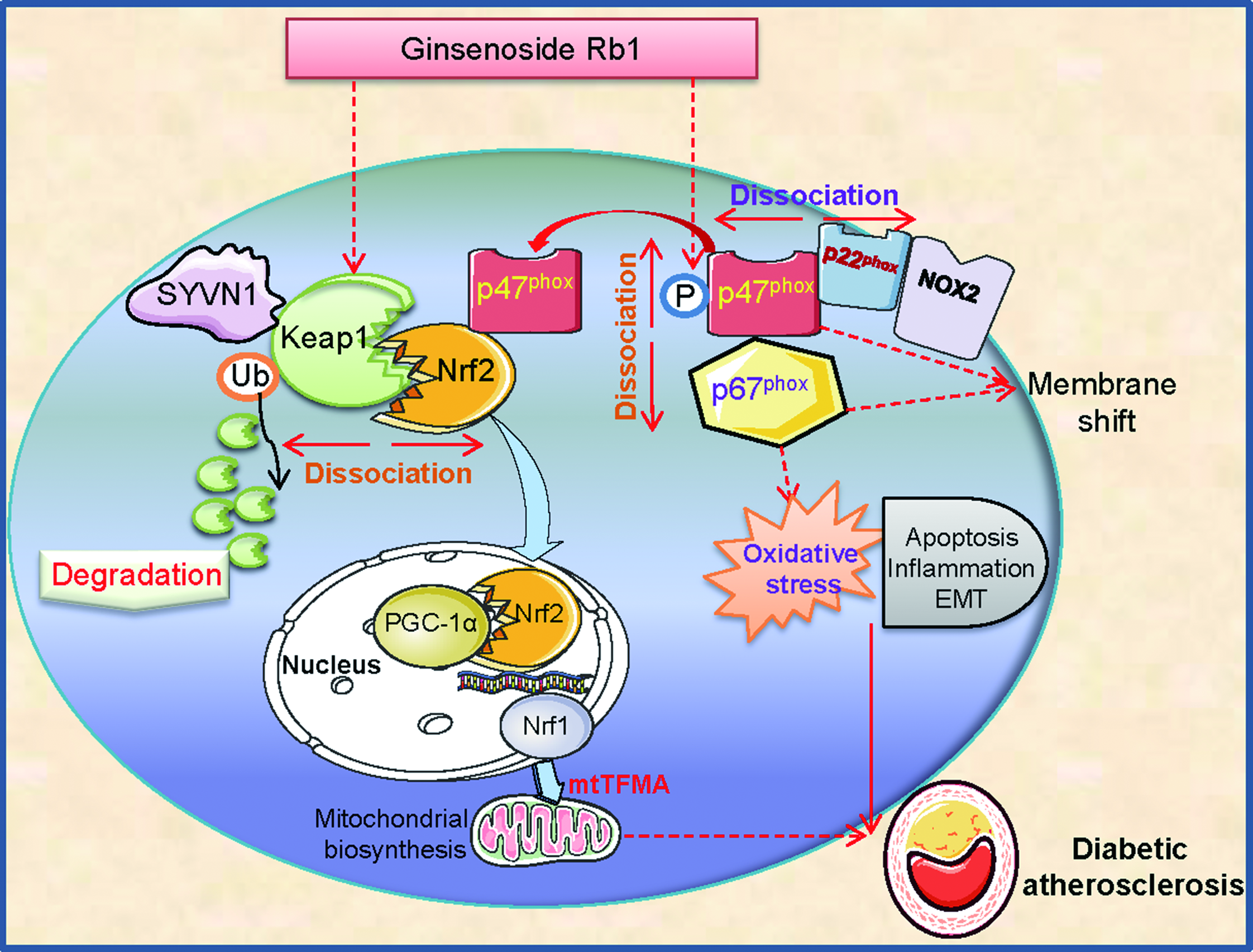

Supplement: Supplementary file 16 — Supplementary Figure 13 [file 41419_2022_5274_MOESM16_ESM.tif]
