# Supplementary material for: A dual Keap1 and p47phox inhibitor Ginsenoside Rb1 ameliorates high glucose/ox-LDL-induced endothelial cell injury and atherosclerosis
Source: Cell Death Dis. 2022 Sep 26;13(9):824. doi: 10.1038/s41419-022-05274-x (PMC9512801; doi:10.1038/s41419-022-05274-x)
Supplement: Supplementary file 17 — Supplementary Table 1 [file 41419_2022_5274_MOESM17_ESM.docx]

**Supplementary Table 1: The information of the primary and secondary antibodies used in this study.**

| **Antibody** | **Source** | **Host/MW** | **Dilution** |
| --- | --- | --- | --- |
| Cleaved-caspase3 | CST: #9661 | Rabbit, 17 kDa | 1:1000 for WB |
| Cytochrome C | Proteintech: 66264-1-Ig | Mouse, 15 kDa | 1:1000 for WB |
| COX-IV | Proteintech: 66110-1-Ig | Mouse, 17 kDa | 1:1000 for WB |
| CD31 | Proteintech: 11265-1-AP | Rabbit, 120 kDa | 1:1000 for WB |
| α-SMA | Proteintech: 14395-1-AP | Rabbit, 42 kDa | 1:1000 for WB |
| Keap1 | Proteintech: 10503-2-AP | Mouse, 70 kDa | 1:1000 for WB |
| HO-1 | Proteintech: 66743-1-Ig | Mouse, 33 kDa | 1:1000 for WB |
| Nrf2 | Proteintech: 66504-1-Ig | Mouse, 110 kDa | 1:1000 for WB |
| PGC1-α | Santa Cruz: sc-518025 | Mouse, 115 kDa | 1:200 for WB |
| Nrf1 | Proteintech: 12936-1-AP | Rabbit, 65 kDa | 1:1000 for WB |
| Ub | Santa Cruz: sc-8017 | Mouse, NA | 1:200 for WB |
| His-Tag Monoclonal antibody | Proteintech: 66005-1-Ig | Mouse, N/A | 1:1000 for WB |
| Flag-Tag Monoclonal antibody | Proteintech: 66008-4-Ig | Mouse, N/A | 1:1000 for WB |
| SYVN1 | Santa Cruz: sc-293484 | Mouse, 85 kDa | 1:200 for WB |
| HA Tag Monoclonal antibody | Proteintech: 66006-2-Ig | Mouse, N/A | 1:1000 for WB |
| NOX2 | Proteintech: 19013-1-AP | Rabbit, 55 kDa | 1:1000 for WB |
| p22^phox^ | Santa Cruz: sc-271968 | Mouse, 22 kDa | 1:200 for WB |
| p47^phox^ | Santa Cruz: sc-17845 | Mouse, 47 kDa | 1:200 for WB |
| p67^phox^ | Santa Cruz: sc-374510 | Mouse, 67 kDa | 1:200 for WB |
| P-p47^phox^ | Abcam: ab74095 | Rabbit, 47 kDa | 1:1000 for WB |
| NKAα1 | Santa Cruz: sc-514614 | Mouse, 100 kDa | 1:200 for WB |
| Goat Anti-Rabbit IgG H&L (Alexa Fluor® 488) | Abcam: ab150077 | Goat, NA | 1:500 for IF |
| [Goat Anti-Mouse IgG H&L (Alexa Fluor® 488)](https://www.abcam.cn/goat-mouse-igg-hl-alexa-fluor-488-ab150113.html) | Abcam: ab150113 | Goat, NA | 1:500 for IF |
| HRP-conjugated Affinipure Goat Anti-Rabbit IgG(H+L) | Proteintech: SA00001-2 | Goat, NA | 1:5000 for WB |
| HRP-conjugated Affinipure Goat Anti-Mouse IgG(H+L) | Proteintech: SA00001-1 | Goat, NA | 1:5000 for WB |
| β-tubulin | Proteintech: 66240-1-Ig | Mouse, 55 kDa | 1:1000 for WB |
| β-actin | Bisoss: bs-0061R | Rabbit, 42 kDa | 1:5000 for WB |
| GAPDH | Bisoss: bs-2188R | Rabbit, 36 kDa | 1:5000 for WB |
| Lamin B1 | Proteintech: 12987-1-AP | Rabbit, 66 kDa | 1:1000 for WB |
