# Supplementary material for: A dual Keap1 and p47phox inhibitor Ginsenoside Rb1 ameliorates high glucose/ox-LDL-induced endothelial cell injury and atherosclerosis
Source: Cell Death Dis. 2022 Sep 26;13(9):824. doi: 10.1038/s41419-022-05274-x (PMC9512801; doi:10.1038/s41419-022-05274-x)
Supplement: Supplementary file 18 — Supplementary Table 2 [file 41419_2022_5274_MOESM18_ESM.docx]

**Supplementary Table 2**. **Compound information and serial numbers in this study** **and their inhibition rate (%) of Keap1 and p47^phox^ luciferase reporter gene activities.**

| **Category** | **No.** | **Compounds** | **Keap1** | **p47^phox^** |
| --- | --- | --- | --- | --- |
| Benzimidazoles | 1 | 5,6-Dimethylbenzimidazole | 18.5 | 25.7 |
| Carboxylic acids | 2 | 1-Naphthaleneacetic acid | 11.1 | -15.8 |
|  | 3 | 10-Hydroxydecanoic Acid | 33.6 | 29.4 |
|  | 4 | D-(+)-Galacturonic acid | 30.1 | 19.2 |
|  | 5 | Mandelic acid | 41.0 | 18.7 |
|  | 6 | Indole-3-acetic acid | 10.5 | 30.9 |
|  | 7 | Oxalic acid | 20.9 | 31.1 |
|  | 8 | 4-Methoxysalicylic acid | 40.1 | 9.6 |
| Curcuminoid | 9 | Bisdemethoxycurcumin | 37.9 | -52.7 |
| Flavonoids | 10 | Oroxin A | 29.8 | -4.1 |
|  | 11 | Procyanidin B1 | 22.8 | 35.9 |
|  | 12 | Hispidulin | 18.6 | -111.1 |
|  | 13 | 4',6,7-Trimethoxyisoflavone | 28.8 | -261.4 |
|  | 14 | Rhamnocitrin | 29.4 | -195.2 |
|  | 15 | Hamaudol | 17.4 | -58.7 |
|  | 16 | Gossypin | 20.4 | 20.0 |
|  | 17 | Dihydrodaidzein | 24.0 | 8.8 |
|  | 18 | Aromadendrin | 22.4 | 13.0 |
|  | 19 | 6,7,4'-Trihydroxyisoflavone | 50.5 | 19.0 |
|  | 20 | 6-Methoxyluteolin | 35.3 | 17.7 |
|  | 21 | 8-Prenylnaringenin | 21.9 | -0.5 |
|  | 22 | Sakuranetin | 14.9 | -78.0 |
|  | 23 | Skullcapflavone II | 15.4 | -69.9 |
|  | 24 | 4',5,7-Trimethoxyflavone | 18.0 | 23.0 |
|  | 25 | (±)-Catechin hydrate | 7.4 | -210.5 |
|  | 26 | 4'-METHOXYFLAVONE | 19.6 | -28.2 |
|  | 27 | 3,4-Dihydroxyflavone | 19.0 | 10.9 |
|  | 28 | 5,7,3',4'-Tetramethoxyflavone | 29.3 | 26.1 |
|  | 29 | Norwogonin | -6.2 | 14.8 |
|  | 30 | Kaempferol 3-gentiobioside | 29.1 | 26.0 |
|  | 31 | Visnagin | 14.9 | 19.5 |
|  | 32 | 5-Hydroxyflavone | 26.7 | 26.3 |
|  | 33 | Aloeresin D | 14.4 | 14.5 |
|  | 34 | EGCG Octaacetate | 22.2 | 11.1 |
|  | 35 | Tilianin | 41.6 | 25.4 |
|  | 36 | 6-Demethoxytangeretin | 23.9 | 15.0 |
|  | 37 | Quercetagetin | 22.3 | 31.1 |
|  | 38 | 4-METHOXYCHALCONE | -3.8 | 36.0 |
|  | 39 | 7-Hydroxyflavanone | 21.4 | 24.7 |
|  | 40 | 3-Methoxyflavone | 45.5 | 15.6 |
|  | 41 | 4-Hydroxyflavanone | 14.8 | -14.4 |
|  | 42 | 2-Hydroxyflavanone | 16.5 | -107.0 |
|  | 43 | 3,6-Dihydroxyflavone | 33.1 | 14.7 |
|  | 44 | 2-Hydroxychalcone | 8.7 | 19.8 |
|  | 45 | 4'-Hydroxychalcone | 20.1 | 28.6 |
|  | 46 | 7-Methoxyflavone | -1.2 | 16.9 |
|  | 47 | Reynoutrin | 28.4 | 26.1 |
|  | 48 | Theaflavin | 29.7 | 26.8 |
|  | 49 | 7-Hydroxyflavone | 18.2 | 20.7 |
|  | 50 | Procyanidin B2 | 22.6 | 23.0 |
|  | 51 | Apigenin-7-glucuronide | 25.8 | 24.7 |
|  | 52 | Sophoraflavanone G | 30.4 | 27.2 |
|  | 53 | Kurarinone | 25.4 | 24.5 |
|  | 54 | Kaempferol 3-glucorhamnoside | 32.9 | 28.5 |
|  | 55 | MOSLOFLAVONE | 29.4 | 26.7 |
|  | 56 | 5,7-DIMETHOXYFLAVONE | 30.5 | 27.2 |
|  | 57 | 5-hydroxy-7,8-dimethoxyflavone | 21.7 | 21.1 |
|  | 58 | Vitexia-glucoside | 25.0 | 28.2 |
|  | 59 | 5-DEMETHYLNOBILETIN | 55.6 | 40.7 |
|  | 60 | 2''-O-β-L-Galorientin | 39.3 | 31.9 |
|  | 61 | GALANGIN-3-METHYLETHER | 12.0 | 17.4 |
|  | 62 | 4',7-DIMETHOXY-5-HYDROXYFLAVONE | 17.2 | 20.1 |
|  | 63 | Maltol | 33.1 | 7.9 |
|  | 64 | Cyanidin Chloride | 36.4 | -19.4 |
|  | 65 | 3’- Methoxy Puerarin | 19.1 | 21.1 |
|  | 66 | Trifolirhizin | 32.3 | 28.2 |
|  | 67 | Quercetin-3-O-β-D-glucose-7-O-β-D-gentiobiosiden | 37.4 | 31.0 |
|  | 68 | HOMOPLANTAGININ | 9.4 | 15.9 |
|  | 69 | Isoliquiritin apioside | 9.4 | 15.9 |
|  | 70 | 7,2'-dihydroxy-3',4'-dimethoxyisoflavane-7-O-glucoside | 30.8 | 27.4 |
|  | 71 | Karanjin | 10.2 | 16.4 |
|  | 72 | 4-Hydroxycoumarin | 26.9 | 14.3 |
|  | 73 | Quercetagitrin | 25.3 | 14.3 |
|  | 74 | Procyanidin C1 | 48.4 | 13.1 |
|  | 75 | Hesperetin 7-O-glucoside | 40.6 | -28.0 |
|  | 76 | Iristectorigenin A | 34.0 | 25.2 |
|  | 77 | Isorhamnetin-3-O-glucoside | 32.5 | 17.3 |
|  | 78 | Quercimeritrin | 44.4 | 21.6 |
|  | 79 | Luteolin-3-O-beta-D-glucuronide | 38.0 | 5.8 |
|  | 80 | Neoeriocitrin | 10.9 | 8.0 |
|  | 81 | Taxifolin 7-O-rhamnoside | 20.7 | 17.5 |
|  | 82 | Vicenin 3 | 30.0 | 34.9 |
| Ginsenosides | 83 | Ginsenoside Rg2 | 11.4 | 29.8 |
|  | 84 | Ginsenoside RD | -1.0 | 37.1 |
|  | 85 | Pseudo-ginsenoside F11 | 15.8 | 33.6 |
|  | 86 | Panax notoginseng Fa | 11.0 | 19.2 |
|  | 87 | Esculin XVII | 13.0 | 27.1 |
|  | 88 | Ginsenoside F1 | 43.7 | 16.9 |
|  | 89 | Notoginsenoside Ft1 | -331.3 | 27.8 |
|  | 90 | Notoginsenoside Fc | 22.8 | 22.2 |
|  | 91 | Notoginsenoside Fe | 17.8 | 53.2 |
|  | 92 | Ginsenoside Rk3 | 23.8 | 52.8 |
|  | 93 | Ginsenoside Rg5 | 20.3 | 45.5 |
|  | 94 | Ginsenoside Rk1 | 32.3 | 38.1 |
|  | 95 | Notoginsenoside R1 | 39.9 | 55.5 |
|  | 96 | (R)-Ginsenoside Rg2 | 46.3 | -46.8 |
|  | 97 | Arialoside A | 38.1 | 45.4 |
|  | 98 | (S)-Panax notoginseng saponins R2 | 45.5 | -64.0 |
|  | 99 | Ginsenoside Rc | 31.7 | 55.8 |
|  | 100 | Ginsenoside Rb3 | 54.9 | 30.5 |
|  | 101 | Ginger-like Panax notoginseng saponins R1 | 27.3 | 40.2 |
|  | 102 | Ginsenoside Rg1 | 57.9 | 28.3 |
|  | 103 | Ginsenoside Re | 20.0 | 29.2 |
|  | 104 | Ginsenoside Rf | 36.8 | -43.9 |
|  | 105 | Ginsenoside Rb2 | 68.4 | 32.3 |
|  | 106 | Ginsenoside Rb1 | 59.3 | 78.3 |
|  | 107 | 20(R)-Ginsenoside Rh2 | 47.9 | 33.5 |
|  | 108 | (S)-Ginsenoside Rh2 | 54.2 | -210.4 |
|  | 109 | Ginsenoside Ro | 29.9 | 43.6 |
|  | 110 | Ginsenoside F2 | 68.7 | -59.2 |
|  | 111 | Ginsenoside Rh1 | 48.3 | 44.2 |
|  | 112 | 20(S)-Ginsenoside Rg3 | 39.9 | 20.3 |
|  | 113 | 20(R)-Ginsenoside Rg3 | 43.7 | 38.9 |
| Glycerolipids | 114 | Glycerol Tri-n-octanoate | 53.5 | 41.9 |
|  | 115 | Glycerol Trieicosanoate | 54.9 | 42.6 |
|  | 116 | Glycerol Tridecanoate | 28.8 | 30.0 |
|  | 117 | Glycerol trilinoleate | 61.9 | 39.3 |
|  | 118 | 1-Oleoyl-rac-glycerol | 60.6 | 27.0 |
| Phenols | 119 | Cannabidiol | 59.7 | 39.5 |
|  | 120 | Hexahydrocurcumin | 23.8 | -45.9 |
|  | 121 | Dimethylcurcumin | 31.2 | 53.0 |
|  | 122 | Chebulinic acid | 38.0 | 33.0 |
|  | 123 | Chebulagic acid | 1.7 | 21.6 |
|  | 124 | 2',3'-Dihydroxy-4'-methoxyacetophenone | 8.4 | 16.5 |
|  | 125 | Homovanillyl alcohol | 2.9 | 9.1 |
|  | 126 | Glucosyringic acid | -5.1 | 32.2 |
|  | 127 | Eugenin | 9.4 | 26.3 |
|  | 128 | Atranorin | 9.5 | 30.3 |
|  | 129 | Isopsoralenoside | 49.0 | 27.3 |
|  | 130 | Psoralenoside | 13.9 | 31.9 |
|  | 131 | Pyromeconic acid | 67.5 | 27.1 |
|  | 132 | Gigantol | 9.2 | 36.5 |
|  | 133 | Chicoric Acid | 66.7 | 40.6 |
|  | 134 | Nordihydroguaiaretic acid | 25.3 | 32.0 |
|  | 135 | Deoxyrhapontin | 7.0 | 24.3 |
|  | 136 | 2-5-dihydroxyacetophenone | 27.1 | 27.6 |
|  | 137 | Homogentisic acid | 25.7 | 36.0 |
|  | 138 | Phenylacetaldehyde | 15.1 | 13.8 |
|  | 139 | 2'-Hydroxyacetophenone | 12.1 | -61.7 |
|  | 140 | BENZYLACETONE | -11.6 | -196.2 |
|  | 141 | trans-Benzylideneacetone | 33.6 | 12.3 |
|  | 142 | Atraric acid | 37.2 | -46.4 |
|  | 143 | 4-Methoxybenzoic acid | 43.6 | 32.5 |
|  | 144 | Forsythoside I | 22.9 | -72.6 |
|  | 145 | Raspberry ketone glucoside | 11.0 | 17.9 |
|  | 146 | 2-HYDROXY-3,4-DIMETHOXYBENZOIC ACID | 20.5 | 32.1 |
|  | 147 | androsin | 45.5 | 35.3 |
|  | 148 | Hydroxytyrosol Acetate | 49.8 | 37.6 |
|  | 149 | Cannabidivarin | 16.2 | 42.1 |
|  | 150 | Gallic aldehyde | 19.7 | 10.4 |
|  | 151 | Anisic aldehyde | 35.7 | 28.7 |
|  | 152 | 2-methoxycinnamaldehyde | 17.4 | -82.2 |
|  | 153 | 3,5-Dimethoxyphenol | 28.1 | 25.7 |
|  | 154 | 6-paradol | 44.4 | 27.6 |
|  | 155 | Gnetol | 23.3 | 8.5 |
|  | 156 | Ginkgolic acid C15:1 | 25.6 | 67.2 |
|  | 157 | Ginkgolic acid C13:0 | -7.4 | 35.1 |
|  | 158 | Ginkgolic acid C17:1 | -5.6 | 57.5 |
|  | 159 | Geraniin | -4.4 | 26.3 |
|  | 160 | Curculigoside | -5.0 | 27.9 |
|  | 161 | 6-Shogaol | 19.1 | 37.4 |
|  | 162 | 8-Gingerol | 2.6 | 32.6 |
|  | 163 | Paeonolide | 21.5 | 41.5 |
|  | 164 | Mulberroside A | 13.9 | 58.3 |
|  | 165 | Vanillin | 7.7 | 37.2 |
|  | 166 | Gossypol | 6.2 | 39.2 |
|  | 167 | Acetovanillone | -0.5 | 39.1 |
|  | 168 | D-DELTA-TOCOPHEROL | 53.6 | 36.4 |
|  | 169 | Orcinol gentiobioside | 15.9 | 44.2 |
|  | 170 | Apiopaeonoside | 18.9 | 33.1 |
|  | 171 | Desmethoxy yangonin | 27.1 | -83.4 |
|  | 172 | Rhaponiticin | 15.0 | 27.4 |
|  | 173 | 4-Ethylphenol | 40.9 | -99.7 |
|  | 174 | Isovanillic acid | 45.0 | 52.4 |
|  | 175 | 4'-Methoxyresveratrol | 39.0 | 42.7 |
|  | 176 | Methylnissolin-3-O-glucoside | 12.8 | -142.5 |
|  | 177 | Acetyl-trans-resveratrol | 57.3 | -131.6 |
|  | 178 | 3,4-Dimethoxybenzaldehyde | 52.0 | 53.1 |
|  | 179 | Zearalenone | 32.1 | 21.6 |
|  | 180 | 4-Hydroxymandelic acid | 27.7 | 42.1 |
|  | 181 | DL -3,4-Dihydroxymandelic acid | 32.1 | 21.6 |
|  | 182 | Agrimol B | 48.4 | -121.9 |
|  | 183 | Dryocrassin ABBA | 22.2 | 35.3 |
|  | 184 | Alnustone | 36.5 | 25.6 |
|  | 185 | DL-Normetanephrine hydrochloride | 18.7 | 38.3 |
|  | 186 | alpha-Arbutin | 46.6 | -344.0 |
|  | 187 | 3,4-Dihydroxyphenylacetic acid | 34.1 | -206.8 |
|  | 188 | 3-Hydroxyphenylacetic acid | 54.7 | 44.3 |
|  | 189 | 4-Methylcatechol | 23.4 | 28.6 |
|  | 190 | 3-Methoxytyramine hydrochloride | 3.5 | 38.3 |
|  | 191 | L-KAWAIN | -11.2 | 49.5 |
|  | 192 | Kakuol | -24.7 | 52.1 |
|  | 193 | Dendrophenol | 7.8 | 37.8 |
|  | 194 | Oxyresveratrol | 8.5 | 27.4 |
|  | 195 | yangonin | 10.5 | -141.3 |
|  | 196 | Thymol | -8.6 | 29.4 |
|  | 197 | Homovanillic acid | 0.8 | 23.6 |
|  | 198 | Punicalagin | -13.9 | -137.5 |
|  | 199 | 10-Gingerol | -15.9 | -47.7 |
|  | 200 | Erianin | -33.1 | 25.6 |
|  | 201 | 7,2'-Dihydroxy-3',4'-dimethoxyisoflavan | -8.7 | 43.1 |
|  | 202 | Pinosylvin | 3.3 | 35.9 |
|  | 203 | Dihydroresveratrol | 26.3 | -94.4 |
|  | 204 | Isorhapontigenin | -9.6 | -236.1 |
|  | 205 | Corilagin | -2.2 | -42.6 |
|  | 206 | 1,2,3,4,6-O-Pentagalloylglucose | -4.0 | 49.0 |
|  | 207 | Rhapontigenin | 8.5 | 38.5 |
|  | 208 | 2'-Hydroxy-5'-methoxyacetophenone | 1.1 | 21.0 |
|  | 209 | 2,6-Dimethoxybenzoic acid | 11.1 | -239.1 |
|  | 210 | Pinostilbene | 35.3 | 27.3 |
|  | 211 | 4-Hydroxybenzyl alcohol | -0.6 | 29.4 |
|  | 212 | Veratric acid | 6.4 | 21.4 |
|  | 213 | Olivetol | 46.9 | 32.8 |
|  | 214 | Tetrahydro Curcumin | -3.2 | 32.3 |
|  | 215 | Methyl gallate | -0.7 | 24.3 |
|  | 216 | Ethyl gallate | -4.5 | 44.3 |
|  | 217 | Methylparaben | -9.0 | 29.7 |
|  | 218 | Methyl syringate | -2.4 | 28.3 |
|  | 219 | β-thujaplicin | -18.9 | -72.8 |
|  | 220 | Sesamol | -1.8 | -389.4 |
|  | 221 | Helicid | -11.7 | 46.7 |
|  | 222 | 3,4-Dihydroxyphenylethanol | 7.0 | 24.4 |
|  | 223 | 6-Gingerol | -3.6 | 22.7 |
|  | 224 | Bakuchiol | -21.4 | 46.4 |
|  | 225 | Protocatechualdehyde | -0.5 | 21.7 |
|  | 226 | Honokiol | 8.6 | -48.1 |
|  | 227 | p-Hydroxybenzaldehyde | -6.0 | 30.0 |
|  | 228 | 5-Hydroxy-1,7-diphenyl-6-hepten-3-one | 44.7 | -114.5 |
|  | 229 | Isoeugenol | 1.1 | 33.8 |
|  | 230 | Chrysophanic Acid | 21.9 | 22.6 |
|  | 231 | Cardamonin | -8.3 | 38.0 |
|  | 232 | 4-Hydroxybenzoic acid | 30.2 | 56.1 |
|  | 233 | (-)-Epigallocatechin Gallate | 19.0 | 43.8 |
|  | 234 | Epigallocatechin | 23.9 | 44.6 |
|  | 235 | Xanthoxyline | 30.8 | -85.2 |
|  | 236 | Sodium Danshensu | 22.1 | 58.9 |
|  | 237 | 3,4,5-Trimethoxyphenol | 28.5 | 47.9 |
|  | 238 | Ethyl Vanillate | 44.8 | 41.6 |
|  | 239 | Paeonol | 38.1 | 51.9 |
|  | 240 | Pterostilbene | 28.2 | 56.7 |
|  | 241 | Phloretic acid | 3.9 | -46.6 |
|  | 242 | Tyrosol | 19.6 | 30.5 |
|  | 243 | Gentisic acid | -11.5 | 36.6 |
|  | 244 | Phloracetophenone | 15.1 | 61.8 |
|  | 245 | Orsellinic acid | 23.9 | 49.9 |
|  | 246 | Morin | 49.7 | 48.2 |
|  | 247 | Ethyl ferulate | 12.9 | 39.3 |
|  | 248 | Caffeic Acid | 17.7 | 58.8 |
|  | 249 | 7-Methoxy-4-methylcoumarin | 7.2 | 11.9 |
|  | 250 | Orsellinic acid ethyl ester | 38.0 | 29.6 |
|  | 251 | (+)-Catechin Hydrate | 15.5 | 27.2 |
|  | 252 | Orcinol glucoside | 37.7 | 44.9 |
|  | 253 | Rosmarinic acid | 8.6 | 36.0 |
|  | 254 | Gossypol acetic acid | 32.9 | 43.5 |
|  | 255 | Salvianolic acid B | 23.0 | 42.6 |
|  | 256 | Methylarbutin | 44.4 | -207.2 |
|  | 257 | Hematoxylin | 40.2 | 33.0 |
|  | 258 | Vitamin E | 12.9 | 50.1 |
|  | 259 | Resveratrol | -47.9 | 43.6 |
|  | 260 | Methyl protocatechuate | 22.5 | 64.3 |
|  | 261 | Terphenyllin | 37.1 | 23.3 |
|  | 262 | Guaiacol | 13.6 | 34.9 |
|  | 263 | Eugenol | 32.3 | 35.4 |
|  | 264 | Vitamin E Acetate | 12.7 | 43.8 |
|  | 265 | 3-Hydroxy-4-methoxyacetophenone | 43.9 | -53.4 |
|  | 266 | Gallic acid | 15.4 | 55.0 |
|  | 267 | Ethylparaben | 45.7 | -68.1 |
|  | 268 | Benzoic acid | -12.6 | 63.9 |
|  | 269 | Cianidanol | 9.4 | 45.5 |
|  | 270 | Tannic acid | -18.0 | -10.7 |
|  | 271 | 3,4-Dimethoxybenzyl alcohol | 32.5 | 51.5 |
|  | 272 | Gallic acid trimethyl ether | -12.7 | 63.7 |
|  | 273 | Methyl EudesMate | 18.3 | 51.7 |
|  | 274 | Protocatechuic acid | -325.6 | 47.8 |
|  | 275 | Ellagic acid | 32.9 | 49.9 |
|  | 276 | Phenylephrine hydrochloride | 0.6 | 30.1 |
|  | 277 | Methyl salicylate | 29.8 | 32.5 |
|  | 278 | Salicylamide | 22.8 | 49.8 |
|  | 279 | Methylsyringol | 3.0 | -133.2 |
|  | 280 | 4-Hydroxyphenylacetonitrile | 42.5 | 65.6 |
|  | 281 | 2'-Hydroxy-4'-methylacetophenone | -8.8 | 49.0 |
|  | 282 | Ethyl 4-hydroxyphenylacetate | 18.7 | 51.1 |
|  | 283 | 4-(4-Methoxyphenyl)-2-butanone | 9.4 | 35.9 |
|  | 284 | Methyl 4-hydroxycinnamate | 8.5 | 18.5 |
|  | 285 | Rubrofusarin-6-O-beta-D-gentiobioside | 32.1 | 31.4 |
|  | 286 | Ethyl salicylate | 35.1 | 26.5 |
| Polyphenols | 287 | kaempferide | 28.5 | 25.1 |
|  | 288 | Cimifugin beta-D-glucopyranoside | -6.4 | 20.4 |
|  | 289 | 5-O-Methylvisammioside | 26.9 | 38.3 |
| Quinones | 290 | Protohypericin | 55.4 | 27.9 |
|  | 291 | alpha-Tocopherolquinone | 20.5 | 32.4 |
|  | 292 | Embelin | 36.7 | 44.6 |
|  | 293 | Acetoxyisovalerylalkannin | 47.4 | 55.3 |
|  | 294 | Acetyl shikonin | 64.5 | 57.1 |
|  | 295 | β,β-Dimethylacrylalkannin | 44.9 | 46.7 |
|  | 296 | Alkannin | 47.8 | 42.1 |
|  | 297 | beta, beta-dimethylacrylshikonin | 46.6 | -64.7 |
|  | 298 | Lapachol | 15.4 | 47.5 |
|  | 299 | Juglone | 71.0 | 50.6 |
|  | 300 | Dimethylacrylshikonin | 42.1 | 31.3 |
|  | 301 | 2-Methoxynaphthoquinone | 46.0 | 12.5 |
|  | 302 | Rheic Acid | 17.6 | 32.4 |
|  | 303 | Lawsone | 50.0 | 16.6 |
|  | 304 | Alizarin | 21.1 | 21.3 |
|  | 305 | Aloe-emodin | 35.7 | 27.6 |
|  | 306 | Plumbagin | 41.2 | 36.8 |
|  | 307 | Ketoisophorone | 63.4 | 39.8 |
|  | 308 | Vitamin K1 | 45.8 | 52.3 |
|  | 309 | Antrapurol | 50.0 | 37.7 |
|  | 310 | Menadione | 31.5 | 31.1 |
| Steroids | 311 | Desacetylcinobufagin | 45.7 | 28.4 |
|  | 312 | Sitostenone | 46.1 | 21.7 |
|  | 313 | Taurodeoxycholate sodium salt | 67.9 | 36.3 |
|  | 314 | Sodium taurochenodeoxycholate | 44.0 | 36.6 |
|  | 315 | Fucosterol | -43.0 | 39.1 |
|  | 316 | Pennogenin 3-O-beta-chacotrioside | 30.5 | 26.2 |
|  | 317 | Qingyangshengenin | 20.0 | 44.9 |
|  | 318 | Polyphyllin VI | 41.8 | 44.7 |
|  | 319 | Tenacissoside H | 64.4 | -156.7 |
|  | 320 | Tenacissoside I | 54.6 | 55.9 |
|  | 321 | Tenacissoside G | 59.2 | -35.3 |
|  | 322 | Ruscogenin | 52.2 | 35.9 |
|  | 323 | Ophiopogonin D | 62.8 | 24.2 |
|  | 324 | Dioscin | 46.0 | -243.7 |
|  | 325 | Periplocin | -116.2 | -83.5 |
|  | 326 | Prosapogenin A | 51.0 | 58.6 |
|  | 327 | Pseudoprotodioscin | -147.0 | -250.6 |
|  | 328 | Bufotaline | 41.1 | 40.1 |
|  | 329 | Arenobufagin | 34.7 | 36.8 |
|  | 330 | Tigogenin | 56.2 | 49.9 |
|  | 331 | (25RS)-Ruscogenin | 9.0 | 41.2 |
|  | 332 | Guggulsterone E&Z | 55.1 | -187.4 |
|  | 333 | GLYCODEOXYCHOLIC ACID | 31.7 | 32.7 |
|  | 334 | Periplogenin | 45.3 | 39.7 |
|  | 335 | Hecogenin | -80.2 | 55.7 |
|  | 336 | TOMATIDINE HYDROCHLORIDE | -253.0 | 39.7 |
|  | 337 | Officinalisinin I | 13.5 | -238.9 |
|  | 338 | Liriopesides B | -129.4 | 45.3 |
|  | 339 | Polyphyllin I | 38.0 | -63.8 |
|  | 340 | Diosgenin glucoside | 51.6 | 39.6 |
|  | 341 | Sitogluside | 15.0 | 18.9 |
|  | 342 | Cyasterone | 50.2 | 37.8 |
|  | 343 | Liriope muscari baily saponins C | 22.5 | 50.1 |
|  | 344 | Gracillin | 46.6 | 38.4 |
|  | 345 | Methyl protodioscin | 26.5 | 30.9 |
|  | 346 | Cinobufagin | 16.9 | 35.6 |
|  | 347 | Timosaponin AIII | -1.0 | 25.1 |
|  | 348 | Timosaponin BII | 23.1 | 26.5 |
|  | 349 | Euphorbiasteroid | 10.2 | 7.7 |
|  | 350 | Brassinolide | 25.0 | 12.2 |
|  | 351 | Caudatin | 2.9 | 37.7 |
|  | 352 | Hyodeoxycholic acid | 13.1 | 28.6 |
|  | 353 | Beta-Sitosterol | 66.3 | 49.0 |
|  | 354 | Deoxycholic acid | 5.8 | 51.0 |
|  | 355 | Cholic Acid | -13.5 | -174.6 |
|  | 356 | Cholesteryl Acetate | 2.3 | 61.4 |
|  | 357 | 5alpha-Cholestan-3-one | 49.6 | -219.7 |
|  | 358 | Cortisone | -18.3 | -341.0 |
|  | 359 | Hydroxyecdysone | 12.4 | 33.5 |
|  | 360 | Glycocholic acid | 21.9 | -68.3 |
|  | 361 | Asiatic acid | -96.1 | -277.6 |
|  | 362 | Madecassic acid | -6.9 | 32.4 |
|  | 363 | Cortodoxone | -54.2 | 34.6 |
|  | 364 | Protodioscin | 9.5 | 12.4 |
|  | 365 | Adrenosterone | -15.5 | 43.9 |
|  | 366 | Lithocholic acid | -2.6 | 32.2 |
|  | 367 | Epiandrosterone | -19.5 | 15.2 |
|  | 368 | Bufalin | -24.2 | 24.4 |
|  | 369 | 5Beta-Pregnane-3Alpha,20alpha-Diol | -12.1 | 27.3 |
|  | 370 | Hydrocortisone | -3.5 | 36.4 |
|  | 371 | Estriol | -25.1 | 9.0 |
|  | 372 | Estradiol | -5.3 | 20.3 |
|  | 373 | Aquacrine | -104.5 | 11.7 |
|  | 374 | Dehydroepiandrosterone | -9.2 | 20.5 |
|  | 375 | Pregnenolone | -15.6 | 32.6 |
|  | 376 | Chenodeoxycholic acid | -8.2 | 41.0 |
|  | 377 | Lactulose | -18.5 | 39.2 |
|  | 378 | Progesterone | 2.8 | 46.4 |
|  | 379 | 7-Ketocholesterol | -14.8 | -228.1 |
