# Supplementary material for: A dual Keap1 and p47phox inhibitor Ginsenoside Rb1 ameliorates high glucose/ox-LDL-induced endothelial cell injury and atherosclerosis
Source: Cell Death Dis. 2022 Sep 26;13(9):824. doi: 10.1038/s41419-022-05274-x (PMC9512801; doi:10.1038/s41419-022-05274-x)
Supplement: Supplementary file 19 — Supplementary Table 3 [file 41419_2022_5274_MOESM19_ESM.docx]

**Supplementary Table 3. Primers sequences for real time PCR.**

| Primers | Sequences (5’-3’) |
| --- | --- |
| Human-GAPDH (Forward) | CCACATCGCTCAGACACCAT |
| Human-GAPDH (Reverse) | CCAGGCGCCCAATACG |
| Human-ICAM1 (Forward) | TCTTCCTCGGCCTTCCCATA |
| Human- ICAM1 (Reverse) | AGGTACCATGGCCCCAAATG |
| Human-IL-1β (Forward) | TCCAGGGACAGGATATGGAG |
| Human-IL-1β (Reverse) | TCTTTCAACACGCAGGACAG |
| Human-IL-6 (Forward) | CCACCGGGAACGAAAGAGAA |
| Human-IL-6 (Reverse) | TCTCCTGGGGGTATTGTGGA |
| Human-CD31 (Forward) | GACGATGTCGGAAACCATGC |
| Human-CD31 (Reverse) | TGAGGACACTTGAACTTCCGT |
| Human-VCAM-1 (Forward) | TTGCTGACAGCTGACCTTTG |
| Human-VCAM-1 (Reverse) | TTTAGGCCACATTGGGAAAG |
| Human-α-SMA(Forward) | AGCCAAGCACTGTCAGGAATC |
| Human-α-SMA (Reverse) | ATGGGGACATTGTGGGTGAC |
| Human-ND1(Forward) | CGGAGTAATCCAGGTCGGTTT |
| Human-ND1 (Reverse) | ATCATTTACGGGGGAAGGCG |
| Human-TFAM (Forward) | CCAAAAAGACCTCGTTCAGCTT |
| Human- TFAM (Reverse) | CTTCAGCTTTTCCTGCGGTG |
| Human-COX1 (Forward) | TCATCTGTAGGCTCATTC |
| Human-COX1 (Reverse) | GGCATCCATATAGTCACT |
| Mouse-GAPDH (Forward) | TCAACGGCACAGTCAAGG |
| Mouse-GAPDH (Reverse) | ACCAGTGGATGCAGGGAT |
| Mouse-TNF-α (Forward) | GTCCCCAAAGGGATGAGAAG |
| Mouse-TNF-α (Reverse) | CACTTGGTGGTTTGCTACGA |
| Mouse-IL-1β (Forward) | CCCAACTGGTACATCAGCACCTC |
| Mouse-IL-1β (Reverse) | GACACGGATTCCATGGTGAAGTC |
| Mouse-MCP-1 (Forward) | CCCCACTCACCTGCTGCTACT |
| Mouse-MCP-1 (Reverse) | TTTACGGGTCAACTTCACATTCAA |
| Mouse-VCAM-1 (Forward) | GCCCTCACTTGCAGCACTAC |
| Mouse-VCAM-1 (Reverse) | TCCTCACCTTCGCGTTTAGT |
| Mouse-IL-6 (Forward) | GGAAATCGTGGAAATGAG |
| Mouse-IL-6 (Reverse) | GCTTAGGCATAACGCACT |
