# Supplementary material for: A dual Keap1 and p47phox inhibitor Ginsenoside Rb1 ameliorates high glucose/ox-LDL-induced endothelial cell injury and atherosclerosis
Source: Cell Death Dis. 2022 Sep 26;13(9):824. doi: 10.1038/s41419-022-05274-x (PMC9512801; doi:10.1038/s41419-022-05274-x)
Supplement: Supplementary file 20 — Supplementary Table 4 [file 41419_2022_5274_MOESM20_ESM.docx]

**Supplementary Table 4**. **Metabolic parameters of nondiabetic and diabetic ApoE^−/−^ mice**.

|  | Control | Model | Model+Rb1+Con shRNA | Model+Rb1+Nrf2 shRNA | Model+Rb1+PGC1-α shRNA |
| --- | --- | --- | --- | --- | --- |
| Body weight (g) | 26.3±0.7 | 22.9±0.6* | 23.7±0.9 | 22.6±0.8* | 22.4±0.9** |
| FBS (mM/L) | 4.7±0.3 | 20.3±1.3***. | 19.1±1.3*** | 20.1±1.6*** | 18.9±1.4*** |
| TG (mM/L) | 0.8±0.1 | 2.5±0.4* | 2.7±0.4** | 2.7±0.5** | 2.7±0.3** |
| TC (mM/L) | 1.8±0.2 | 22.6±3.1*** | 23.9±2.0*** | 26.0±4.0*** | 24.4±2.3*** |
| LDL-C (mM/L) | 1.6±0.5 | 14.6±1.9*** | 14.9±1.3*** | 16.9±2.4*** | 14.0±1.4*** |

Value are expressed as mean ± SE, n=9-11. *P<0.05, **P<0.01 and ***P<0.001 vs. Control. FBS, fasting blood glucose; TG, triglycerides; TC, total cholesterol; LDL-C, low density lipoprotein cholesterol.
