# Supplementary material for: A dual Keap1 and p47phox inhibitor Ginsenoside Rb1 ameliorates high glucose/ox-LDL-induced endothelial cell injury and atherosclerosis
Source: Cell Death Dis. 2022 Sep 26;13(9):824. doi: 10.1038/s41419-022-05274-x (PMC9512801; doi:10.1038/s41419-022-05274-x)
Supplement: Supplementary file 21 — Supplementary file of western blot. [file 41419_2022_5274_MOESM21_ESM.pdf]

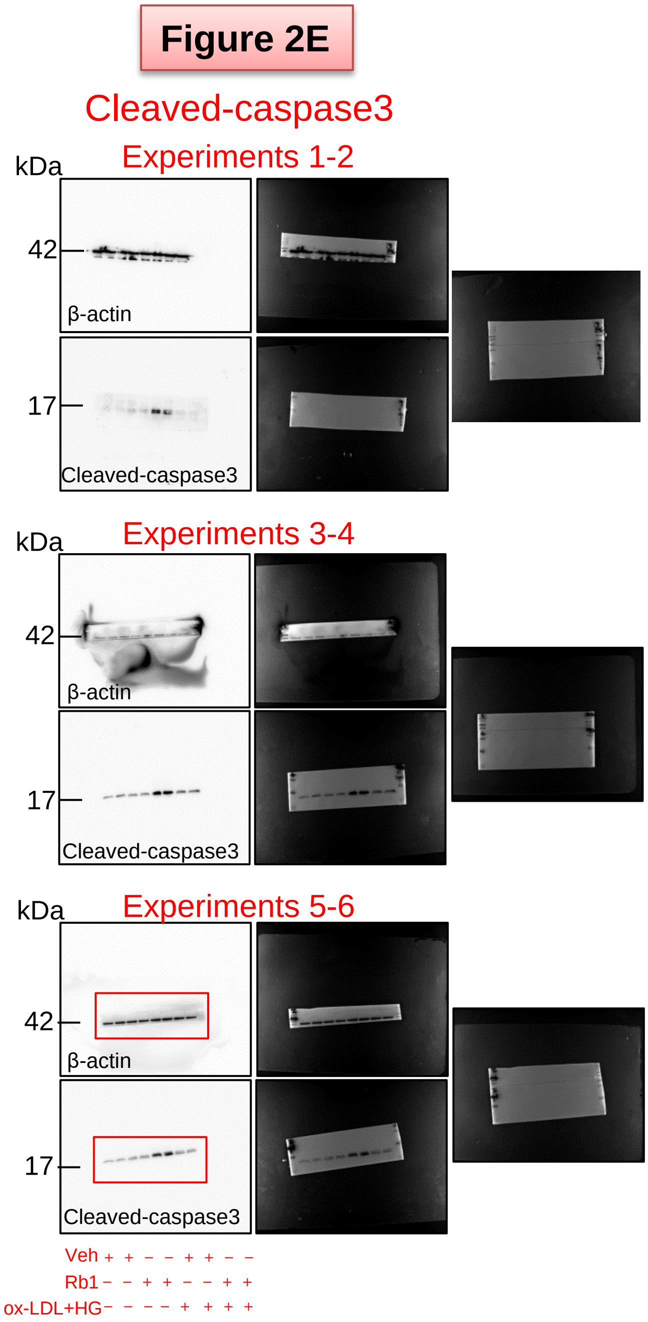
Figure 2E
Cleaved-caspase3
Experiments 1-2
kDa
42
β-actin
17
Cleaved-caspase3
Experiments 3-4
kDa
42
β-actin
17
Cleaved-caspase3
Experiments 5-6
kDa
42
β-actin
17
Cleaved-caspase3
Veh + + − − + + − −
Rb1 − − + + − − + +
ox-LDL+HG − − − − + + + +

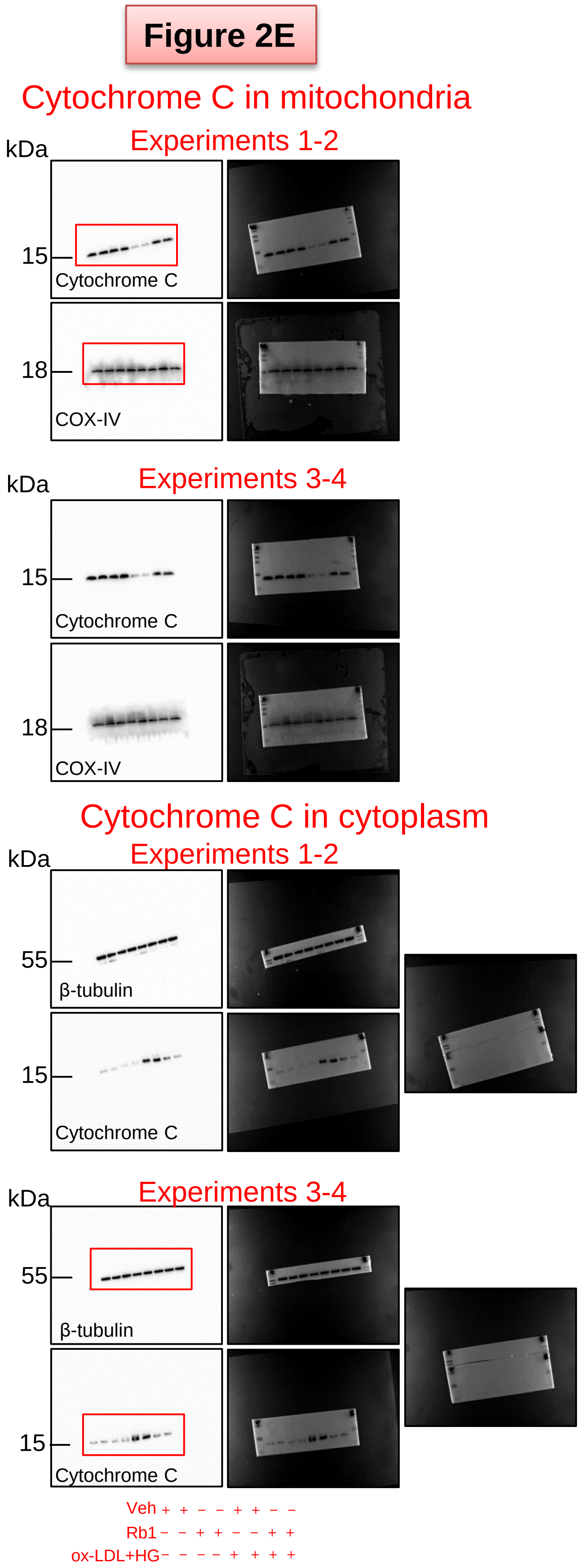
Figure 2E
Cytochrome C in mitochondria
Experiments 1-2
kDa
15
Cytochrome C
18
COX-IV
Experiments 3-4
kDa
15
Cytochrome C
18
COX-IV
Cytochrome C in cytoplasm
Experiments 1-2
kDa
55
β-tubulin
15
Cytochrome C
Experiments 3-4
kDa
55
β-tubulin
15
Cytochrome C
Veh + + − − + + − −
Rb1 − − + + − − + +
ox-LDL+HG − − − − + + + +

Figure 2I

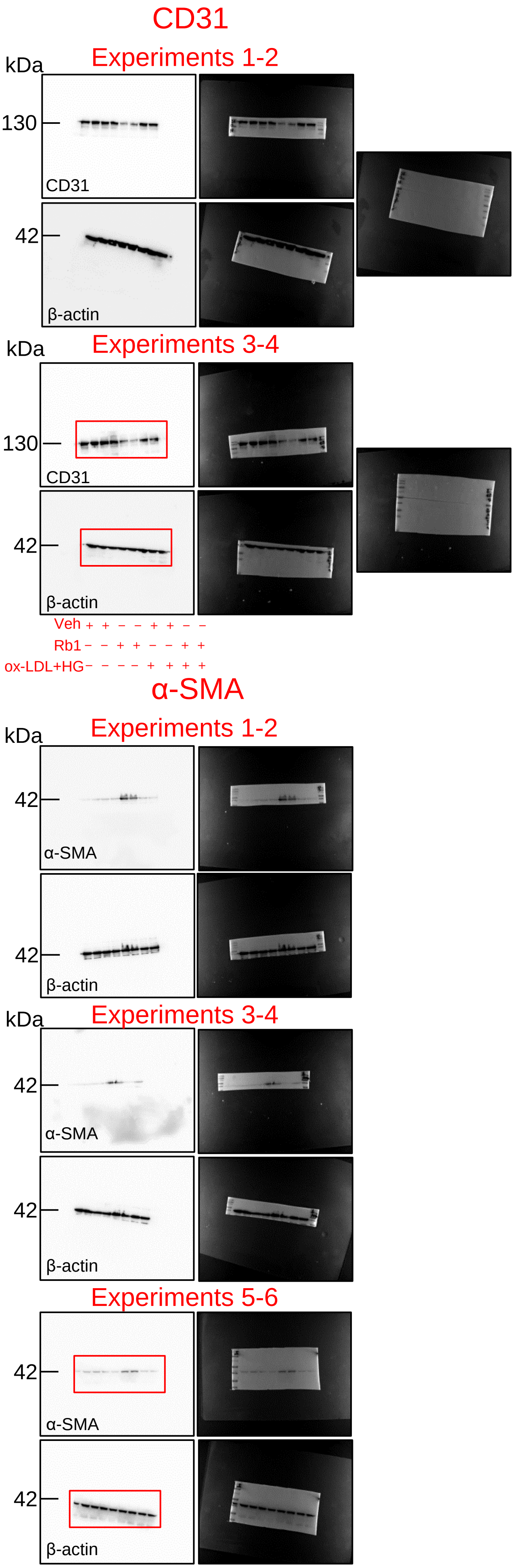

Figure 4A

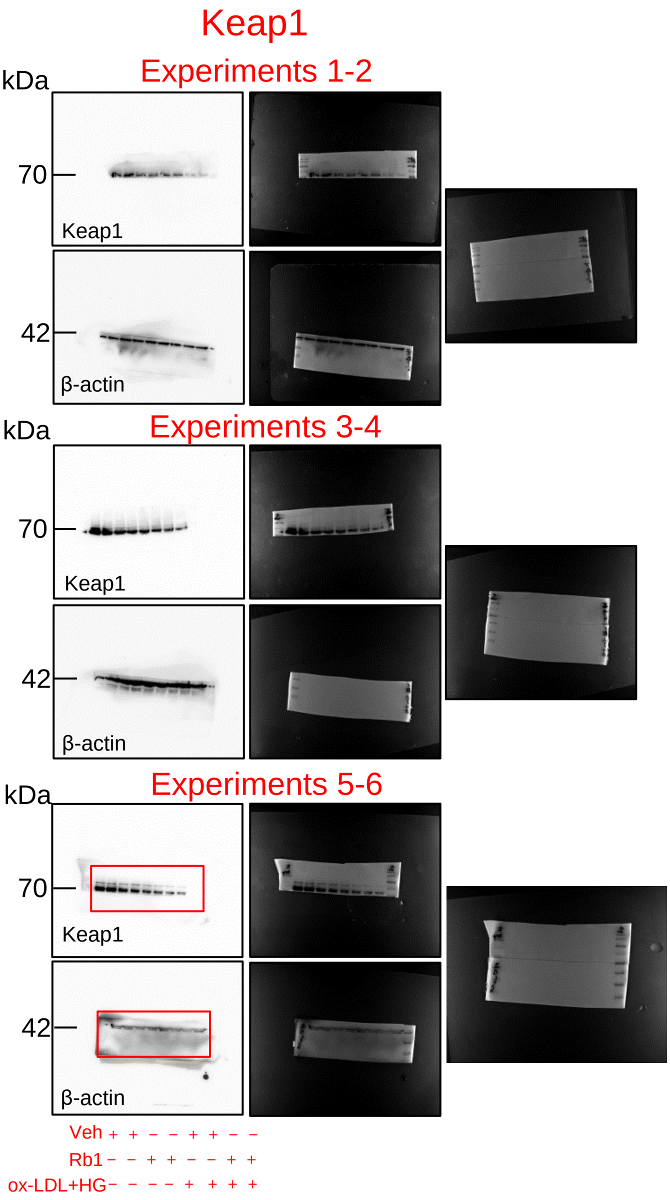

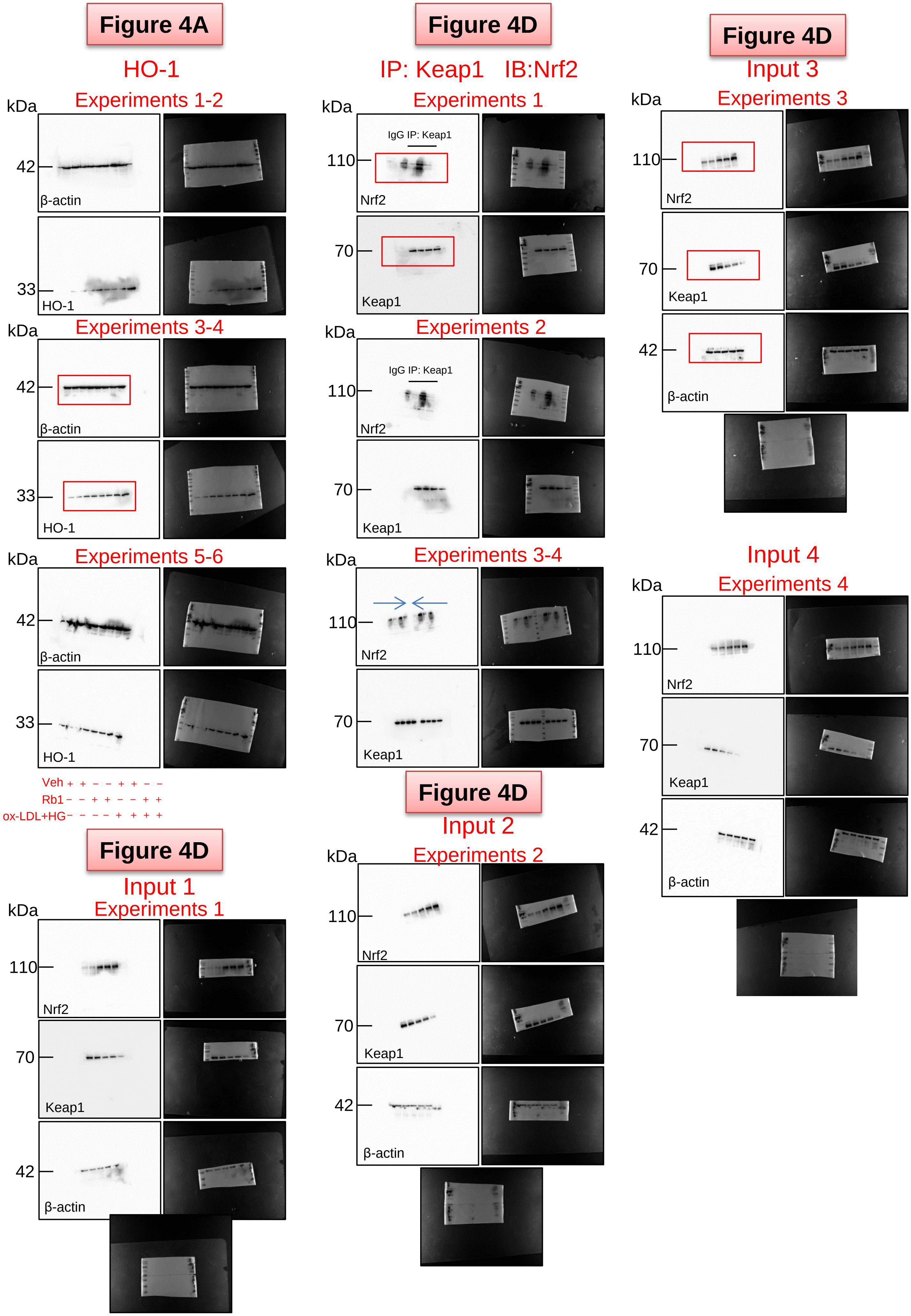
Figure 4A
HO-1
Experiments 1-2
kDa
42
β-actin
33
HO-1
Experiments 3-4
kDa
42
β-actin
33
HO-1
Experiments 5-6
kDa
42
β-actin
33
HO-1
Veh + + − − + + − −
Rb1 − − + + − − + +
ox-LDL+HG − − − − + + + +
Figure 4D
Input 1
Experiments 1
kDa
110
Nrf2
70
Keap1
42
β-actin
Figure 4D
IP: Keap1 IB:Nrf2
Experiments 1
kDa
IgG IP: Keap1
110
Nrf2
70
Keap1
Experiments 2
kDa
IgG IP: Keap1
110
Nrf2
70
Keap1
Experiments 3-4
kDa
110
Nrf2
70
Keap1
Figure 4D
Input 2
Experiments 2
kDa
110
Nrf2
70
Keap1
42
β-actin
Figure 4D
Input 3
Experiments 3
kDa
110
Nrf2
70
Keap1
42
β-actin
Input 4
Experiments 4
kDa
110
Nrf2
70
Keap1
42
β-actin

Figure 4H

Nuclear Nrf2

Experiments 1-2

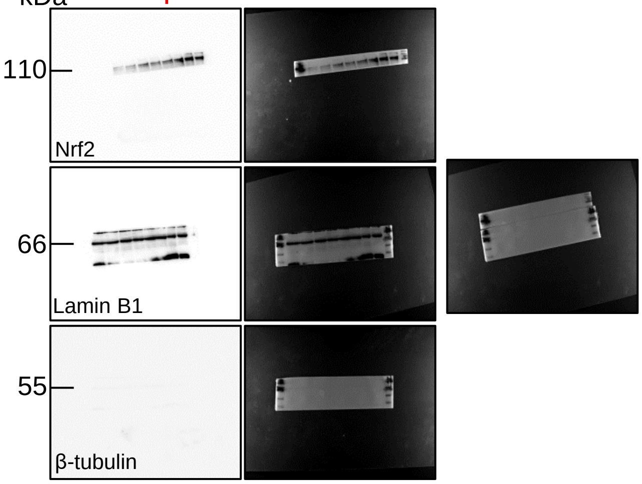

Experiments 3-4

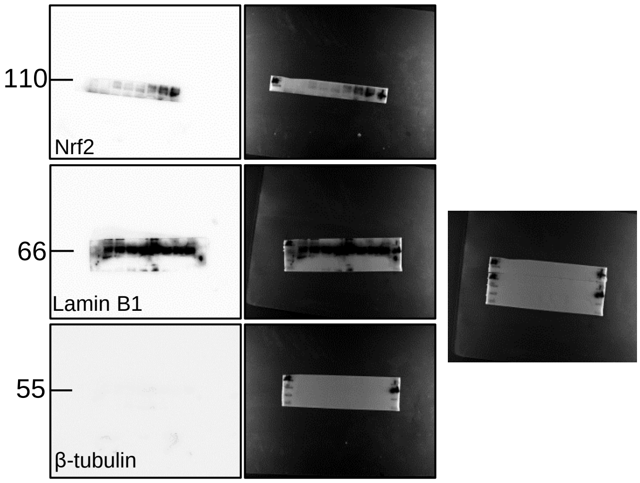

Experiments 5-6

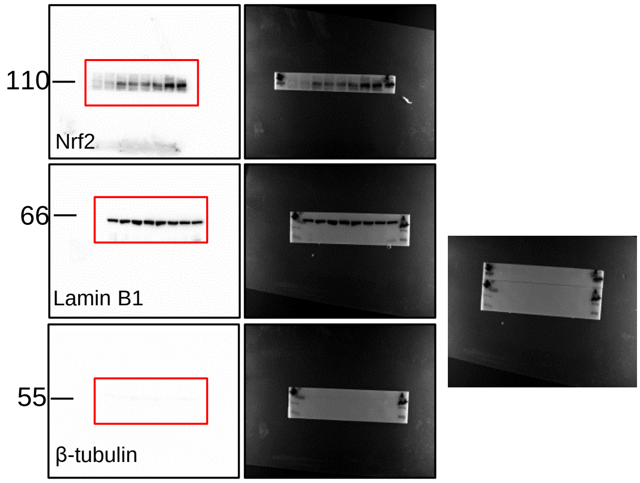

Figure 4H

Cytoplasmic Nrf2

Experiments 1-2

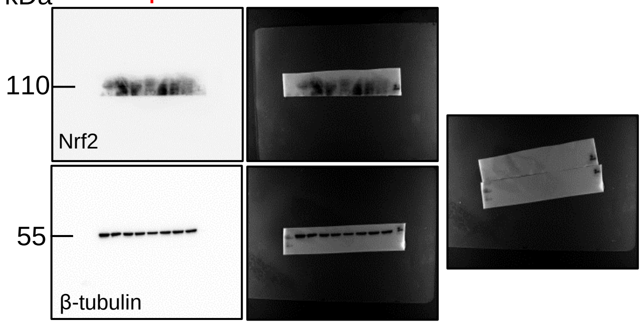

Experiments 3-4

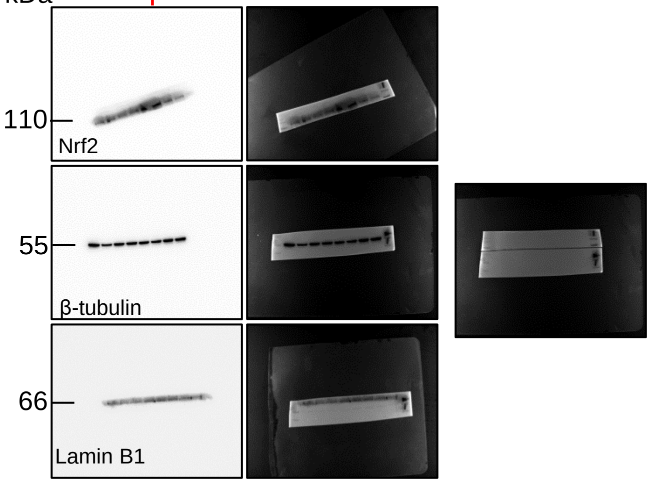

Experiments 5-6

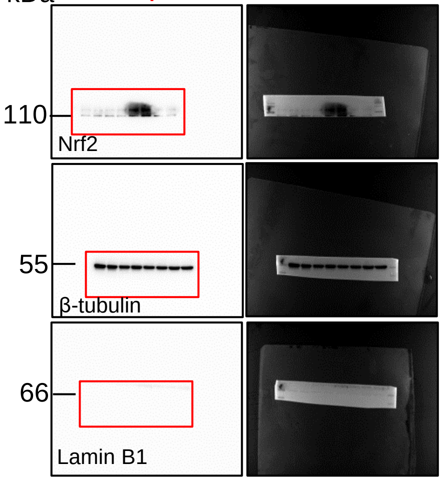

Figure 4J

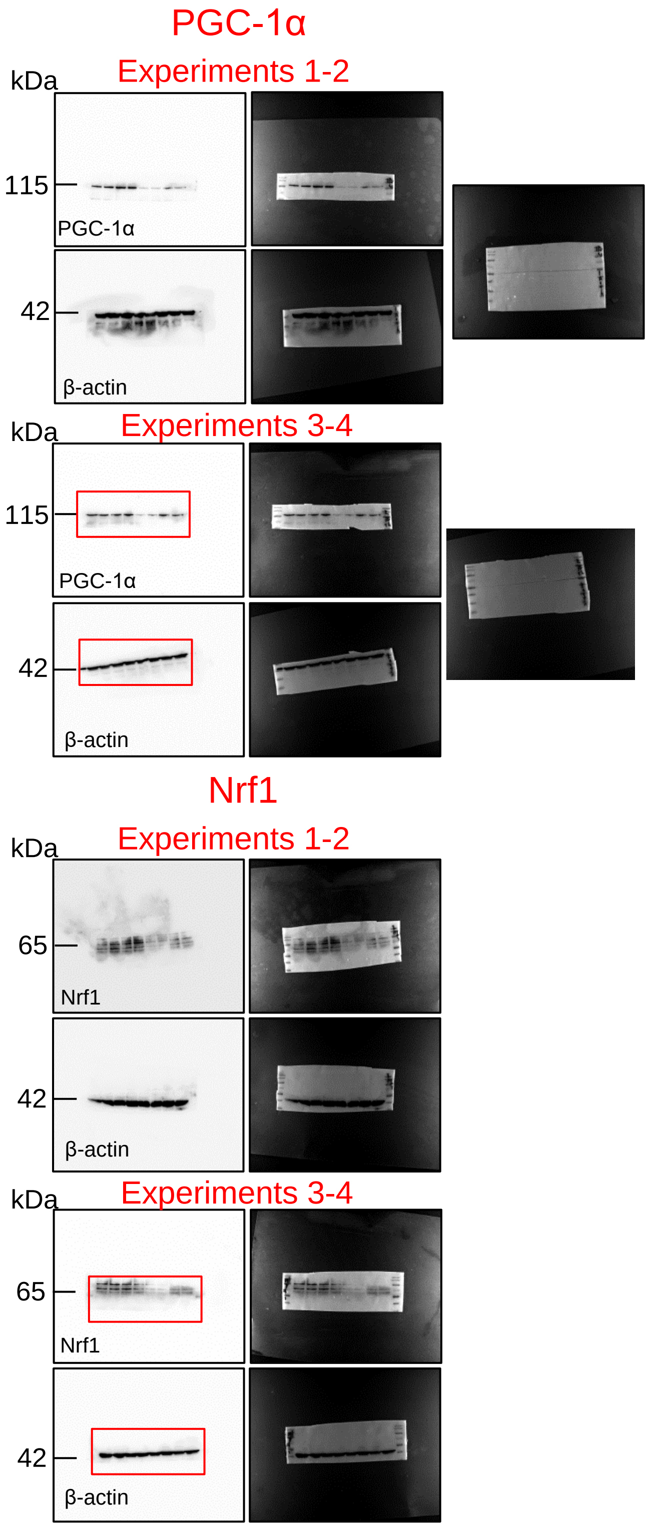

Figure 4K

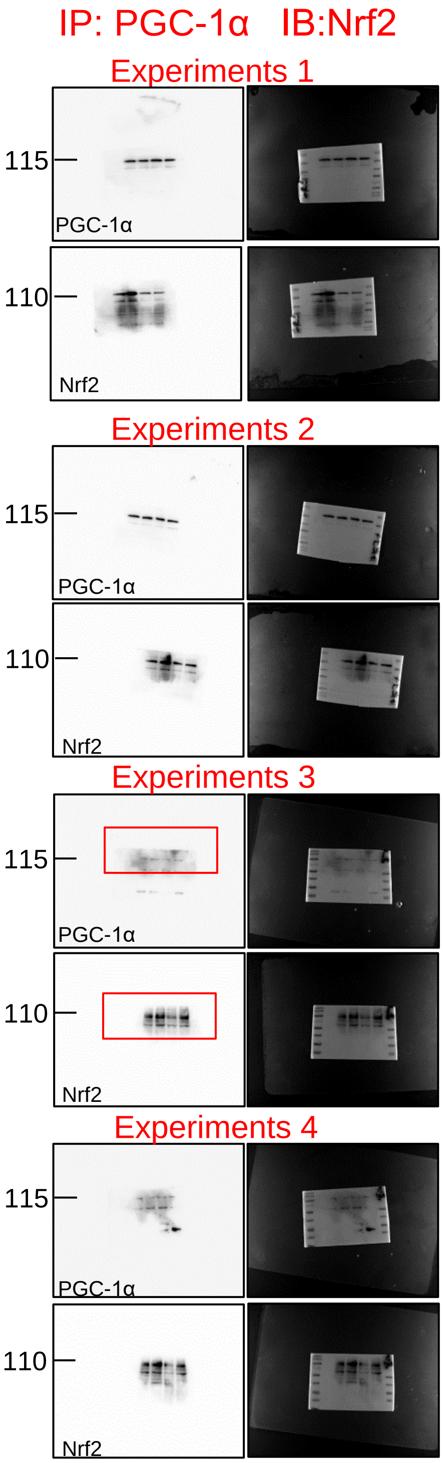

Figure 4K

Input

Experiments 1-2

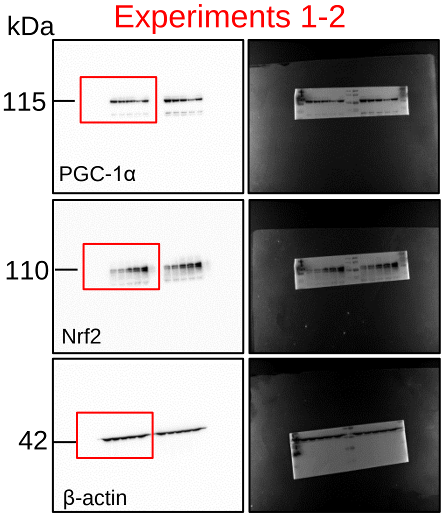

Experiments 3-4

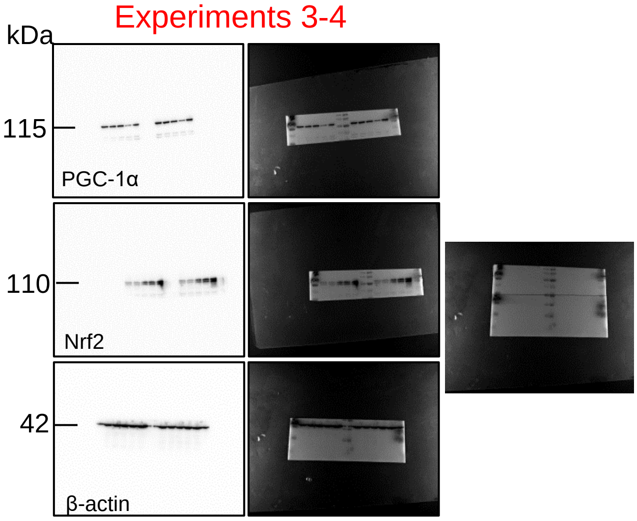

Figure 5A

Keap1-Upper

Experiments 1-2

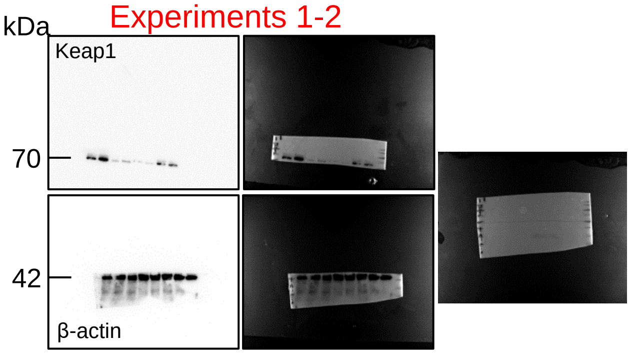

Experiments 3-4

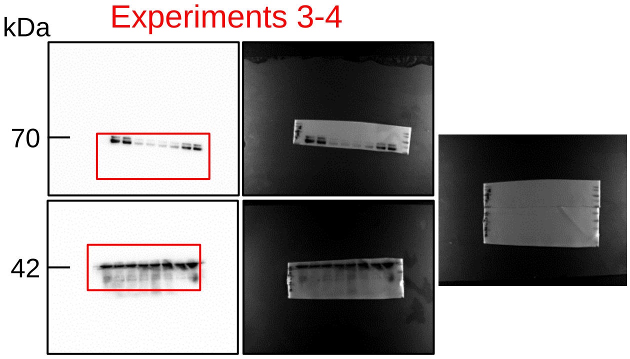

Figure 5A

Keap1-Lower

Experiments 1-2

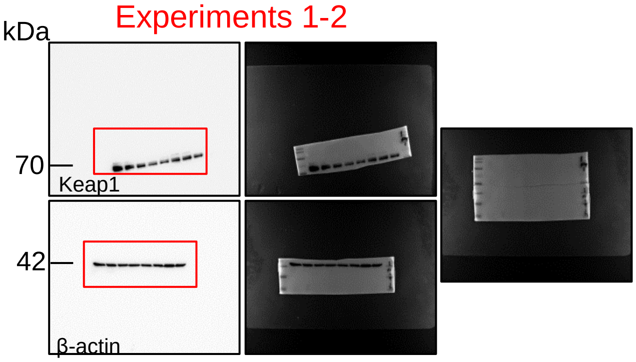

Experiments 3-4

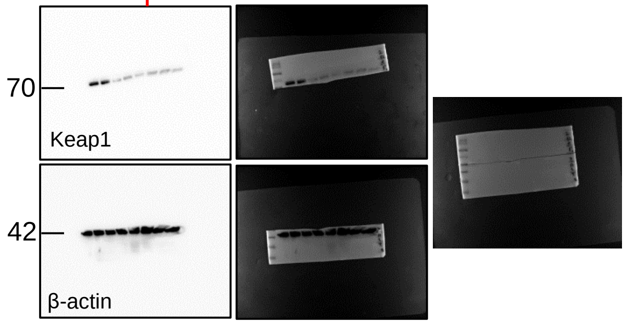

Figure 5B

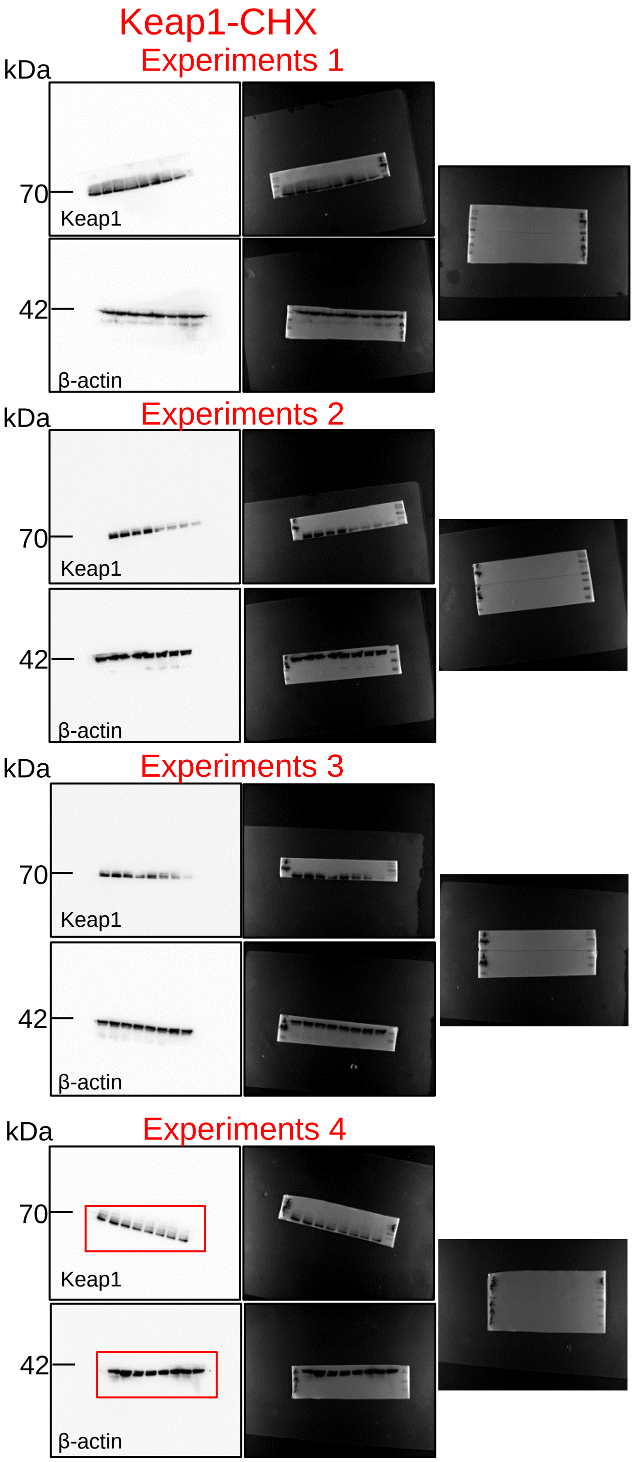

Figure 5B

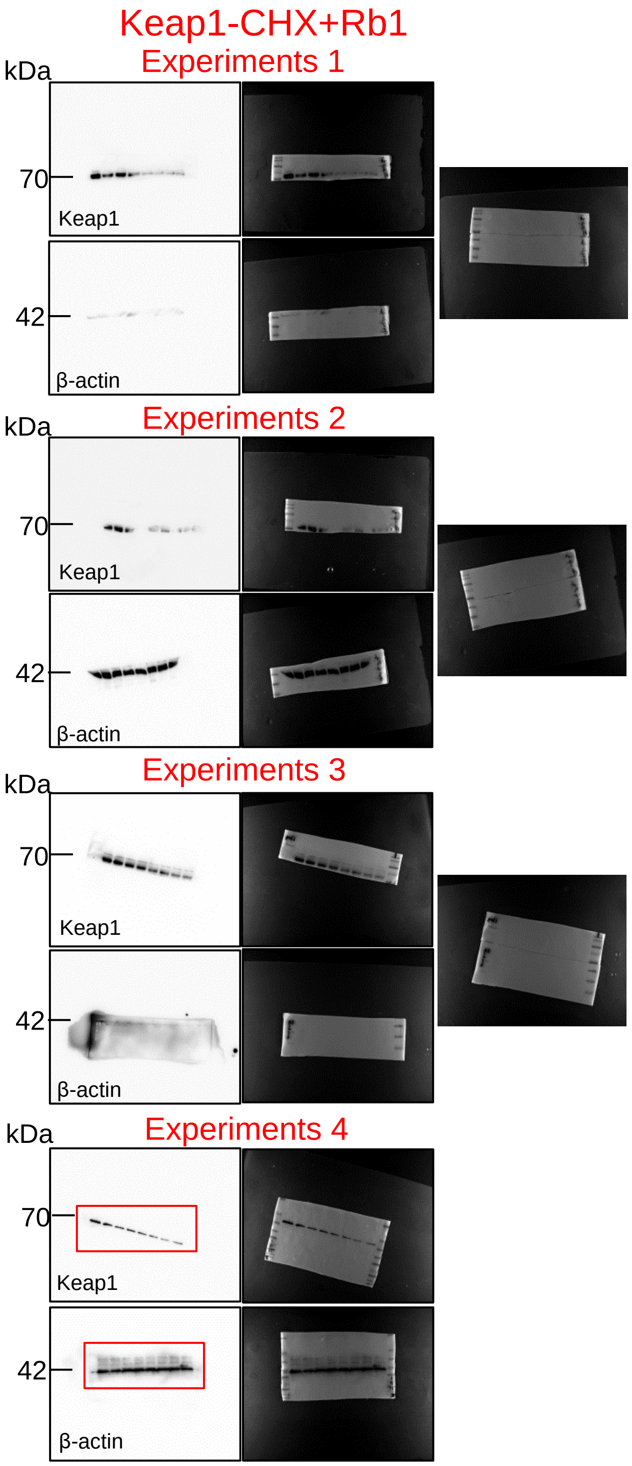

Figure 5C

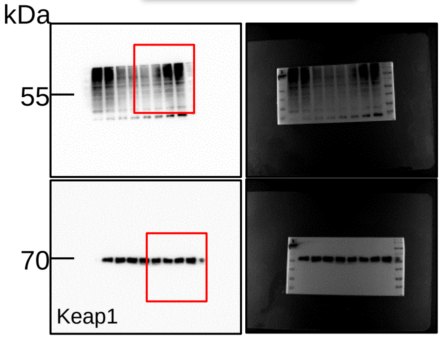

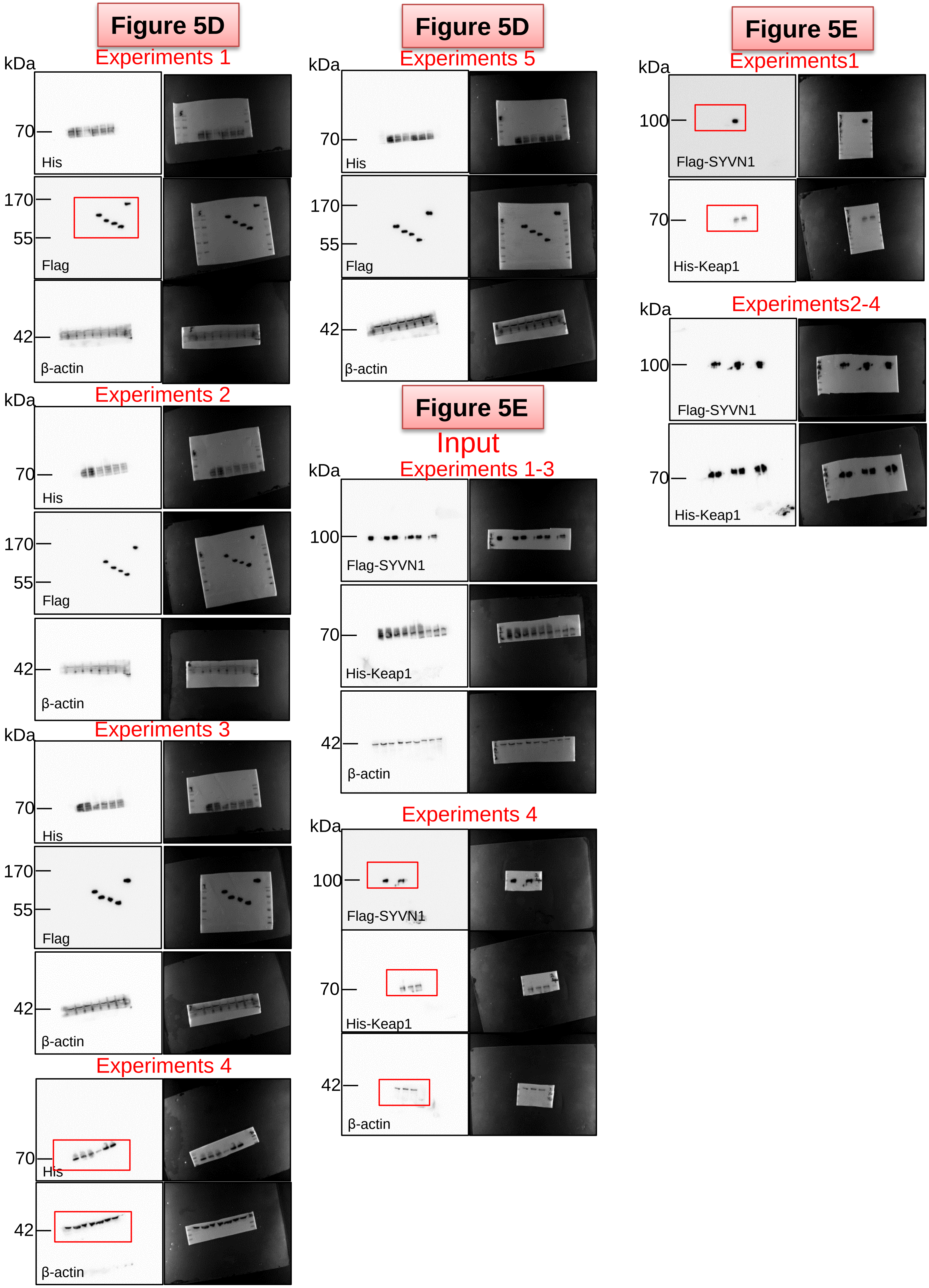
Figure 5D
Experiments 1
kDa
70
His
170
55
Flag
42
β-actin
Experiments 2
kDa
70
His
170
55
Flag
42
β-actin
Experiments 3
kDa
70
His
170
55
Flag
42
β-actin
Experiments 4
70
His
42
β-actin
Figure 5D
Experiments 5
kDa
70
His
170
55
Flag
42
β-actin
Figure 5E
Input
Experiments 1-3
kDa
100
Flag-SYVN1
70
His-Keap1
42
β-actin
Experiments 4
kDa
100
Flag-SYVN1
70
His-Keap1
42
β-actin
Figure 5E
Experiments1
kDa
100
Flag-SYVN1
70
His-Keap1
Experiments2-4
kDa
100
Flag-SYVN1
70
His-Keap1

Figure 5F

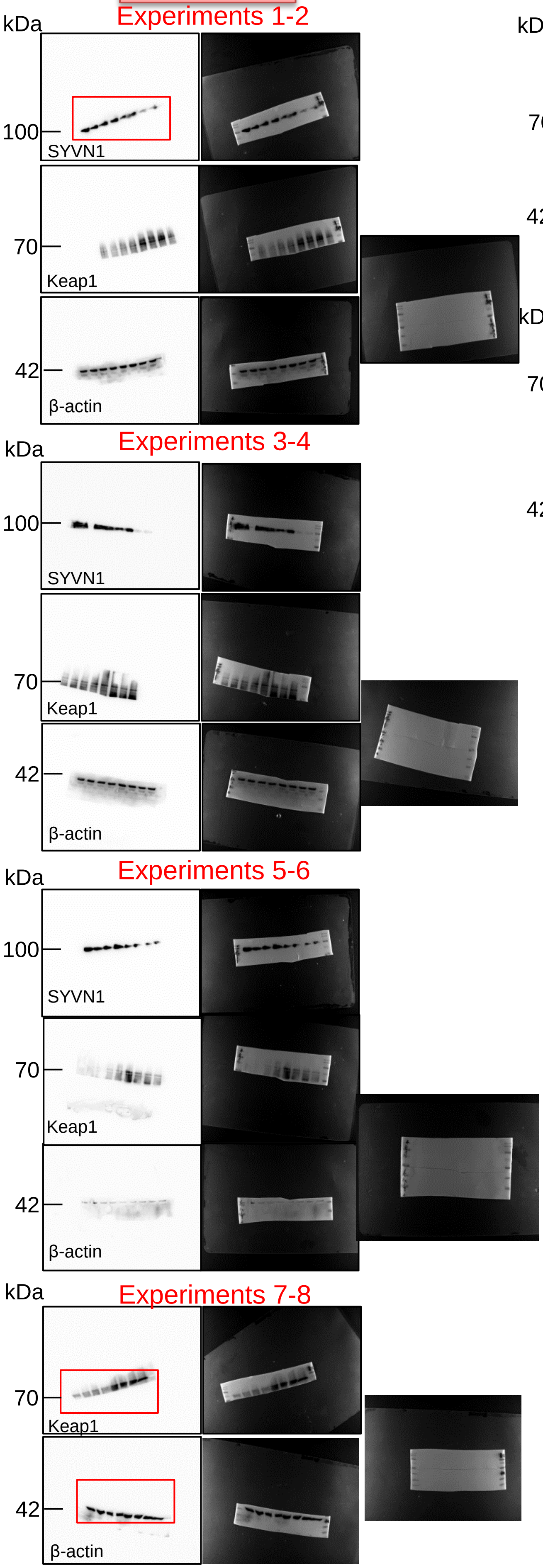

Figure 5I

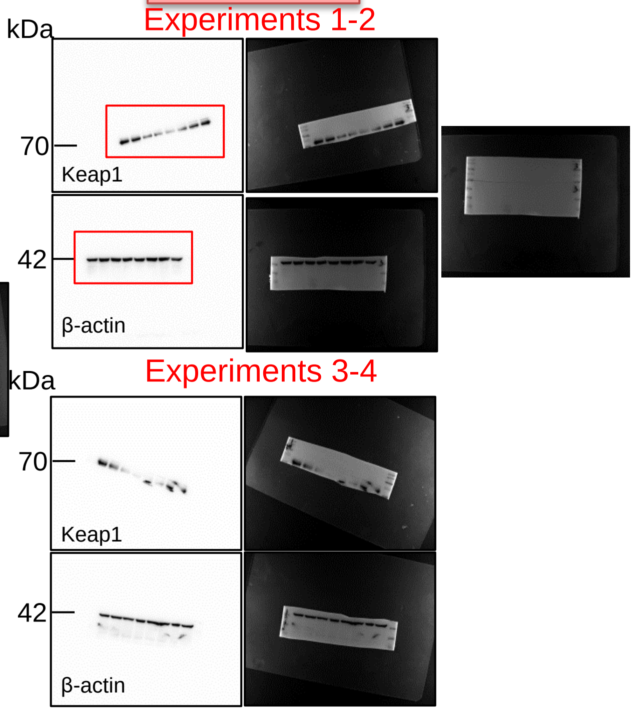

Figure 6B

Experiments 1

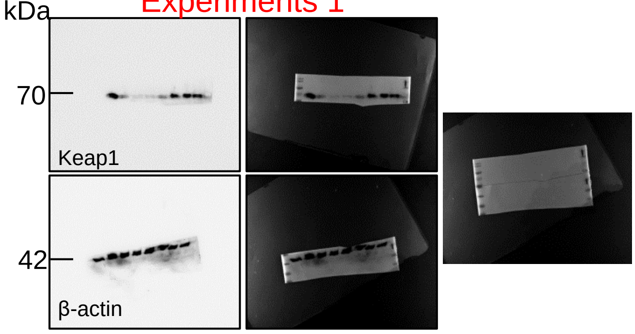

Experiments 2

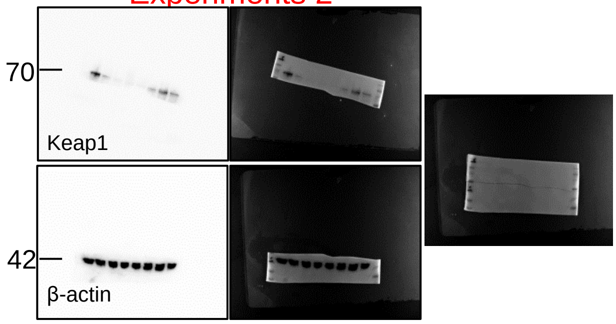

Experiments 3

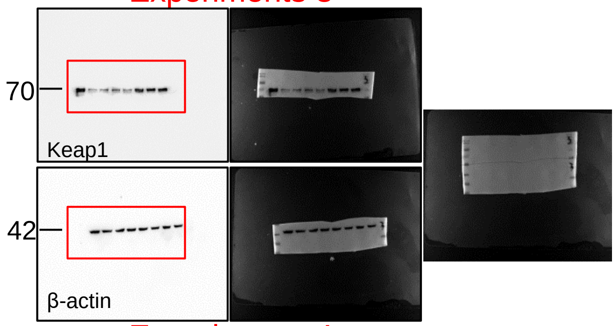

Experiments 4

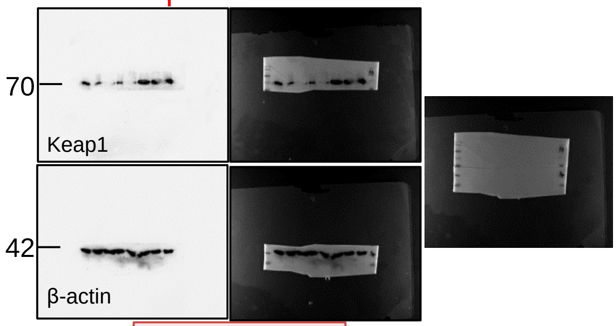

Figure 6C

Experiments 1

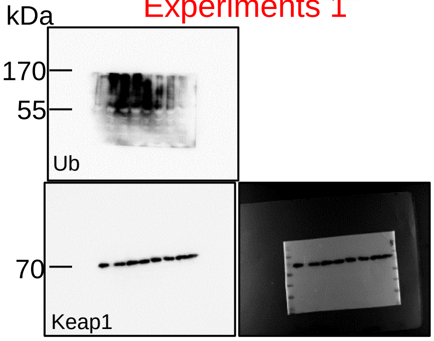

Experiments 2

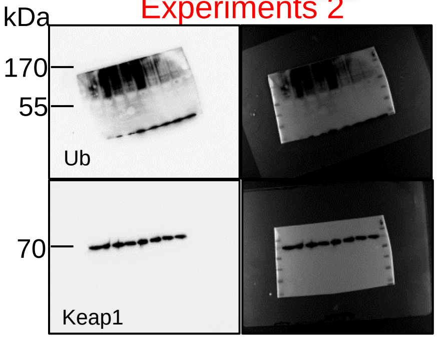

Experiments 3

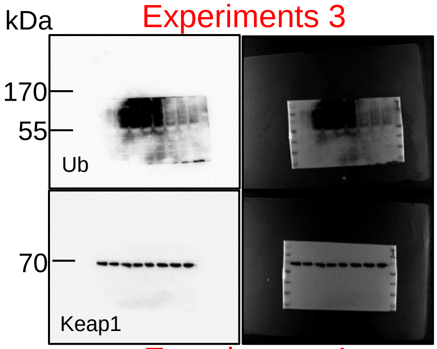

Experiments 4

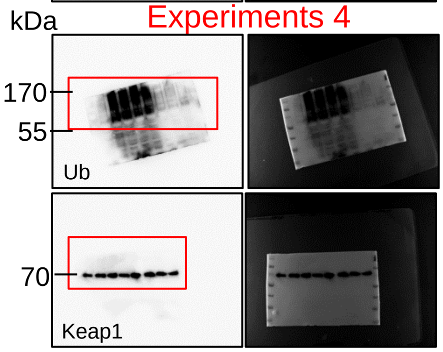

Figure 6D

Experiments 1-2

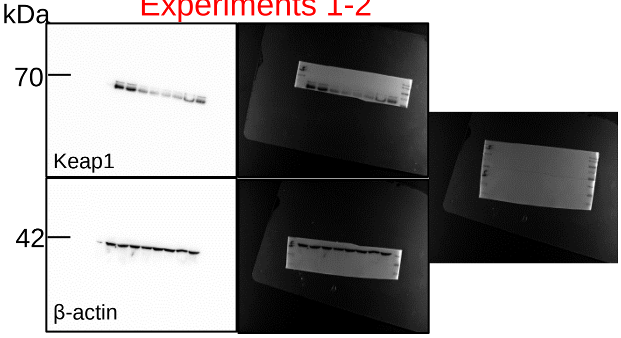

Experiments 3-4

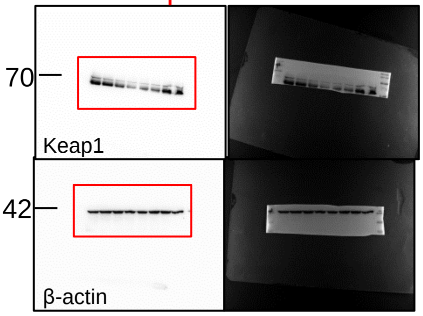

Figure 6E

Experiments 1-2

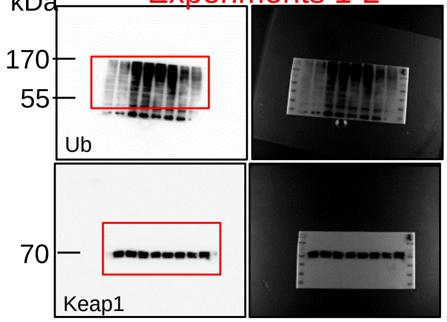

Experiments 3-4

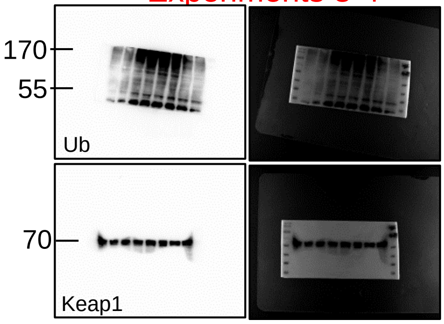

Figure 7A

NOX2

Experiments 1-2

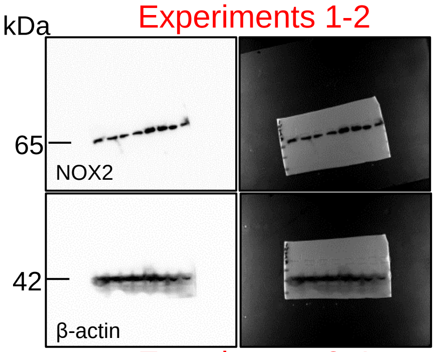

Experiments 3-4

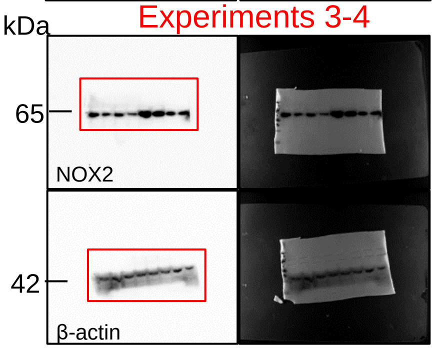

Experiments 5-6

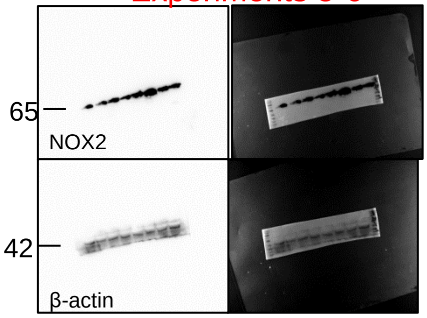

Figure 7A

NOX2

Experiments 7-8

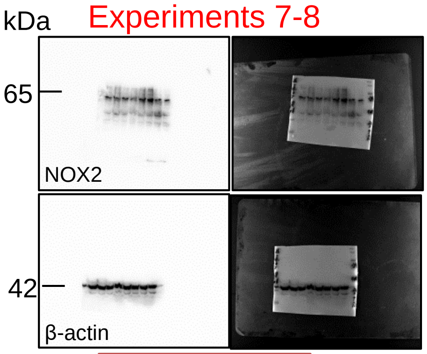

Figure 7A

p22phox

Experiments 1-2

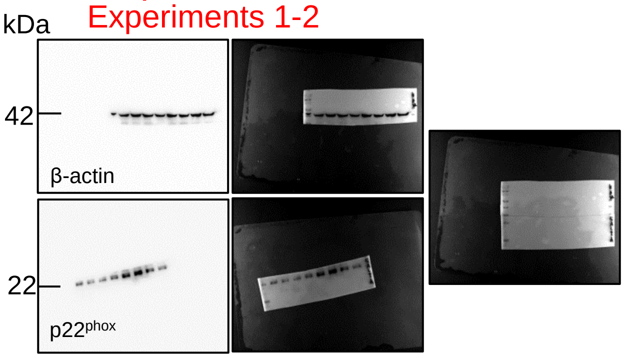

Experiments 3-4

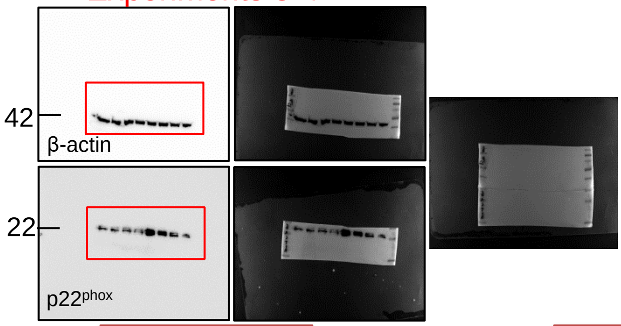

Figure 7B

P-p47phox

Experiments 1-2

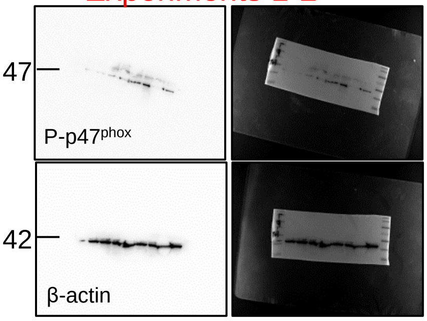

Figure 7B

P-p47phox

Experiments 3-4

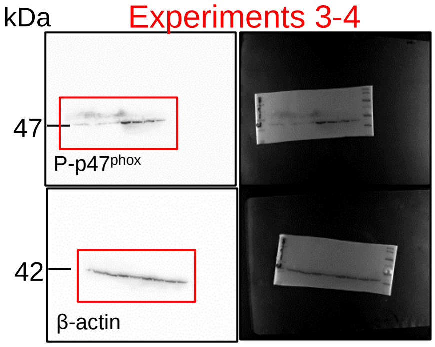

Figure 7C

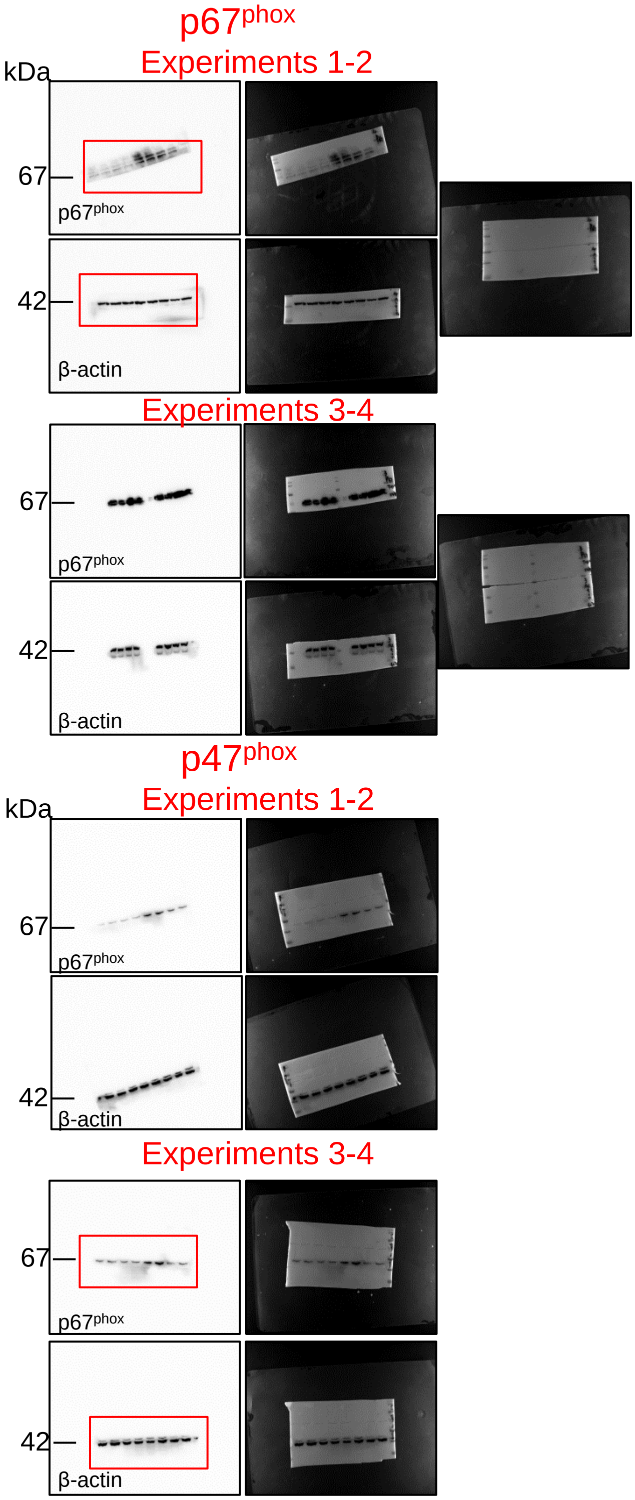

Figure 7D

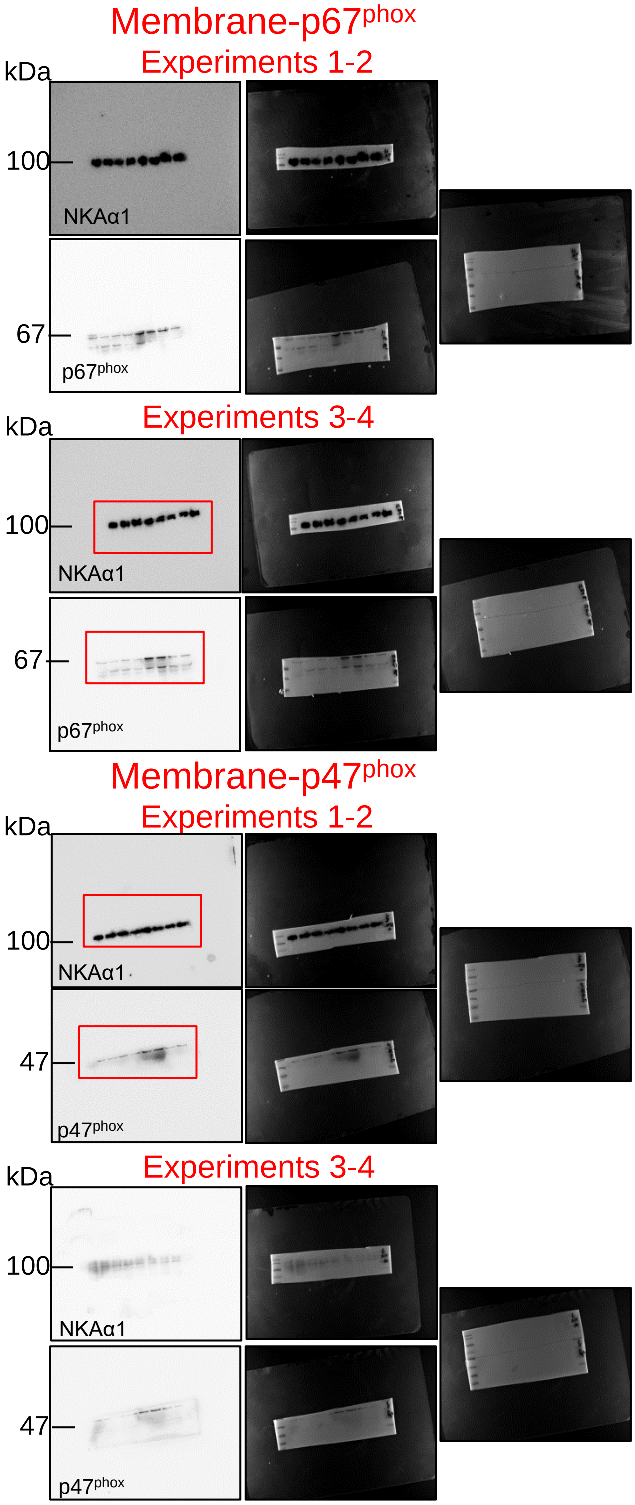

Figure 7E

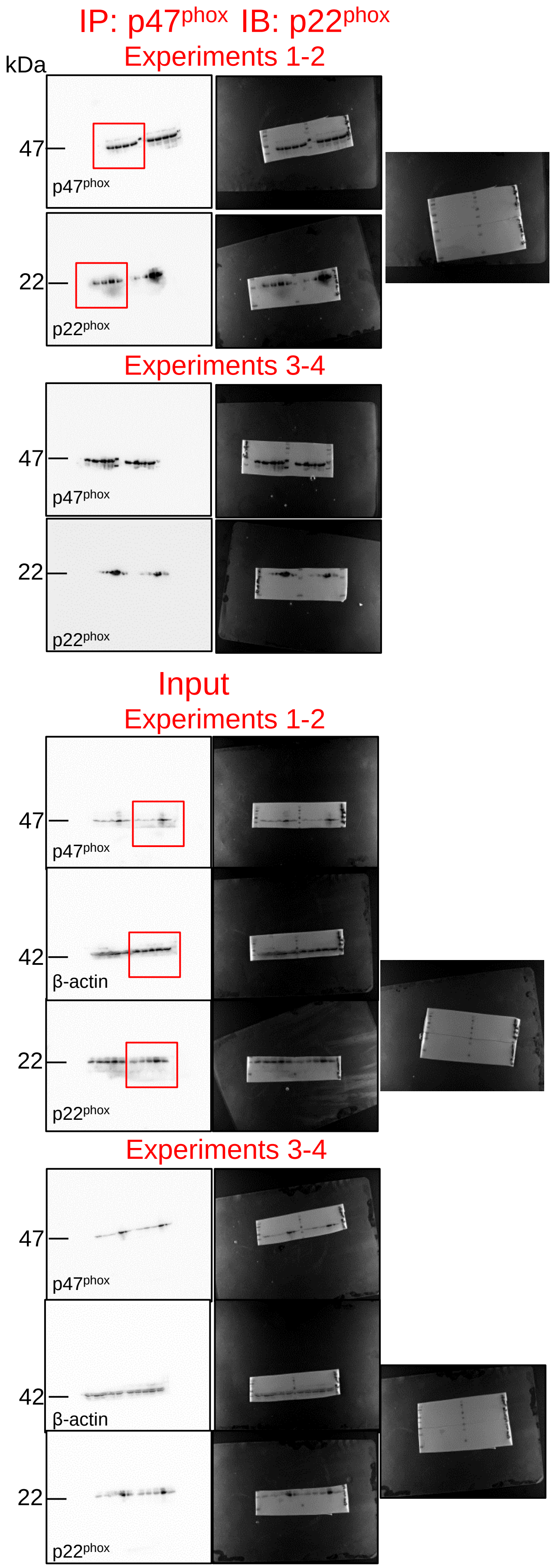

Figure 7F

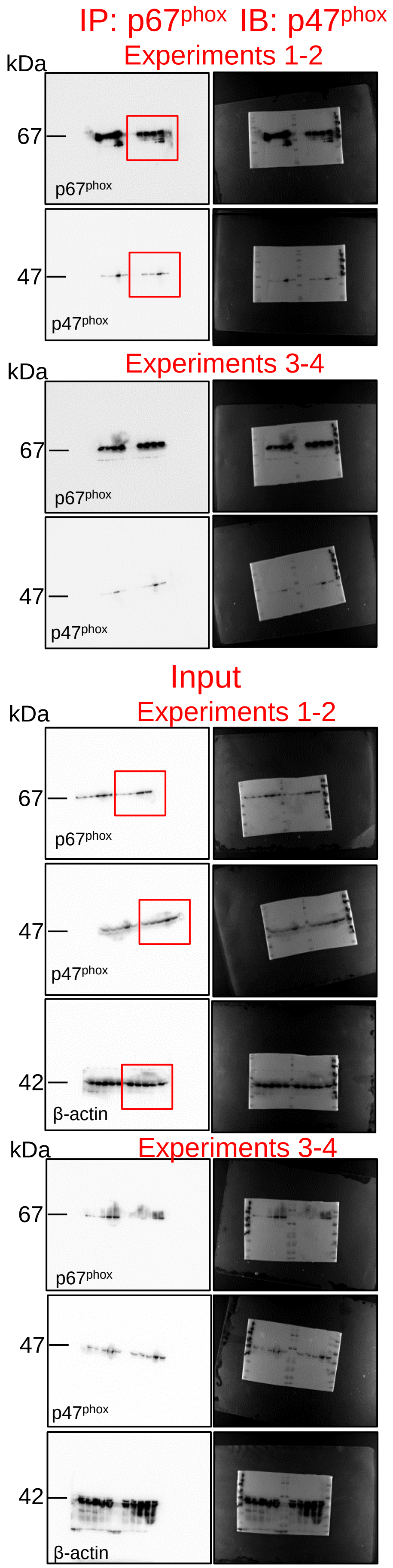

Figure 7G

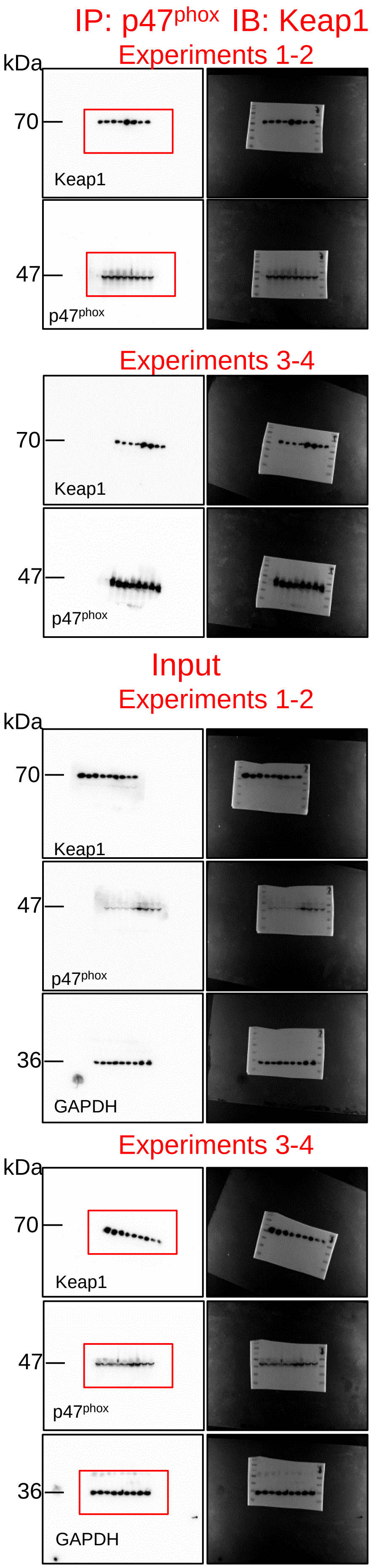

Figure 7H

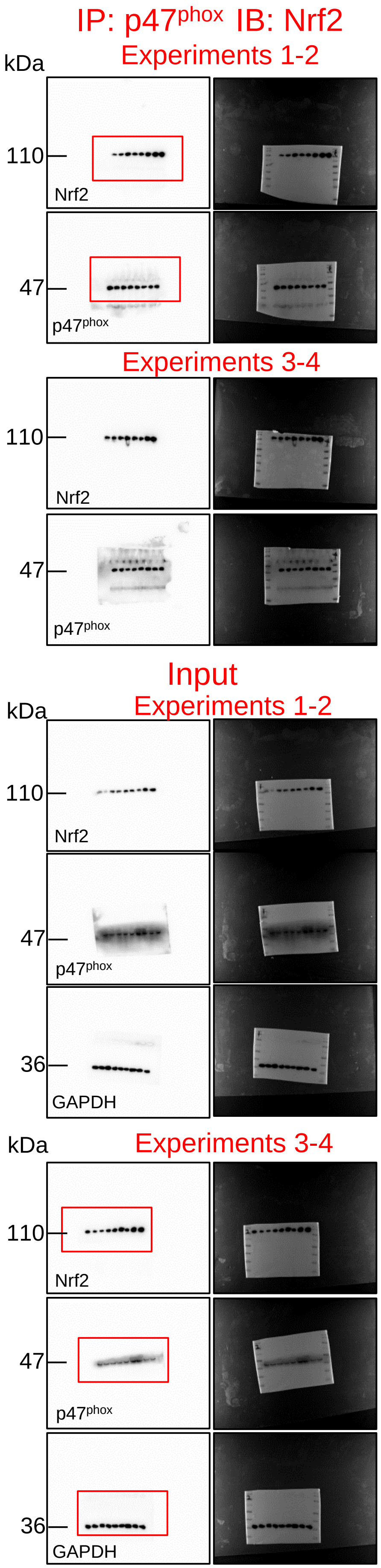

Figure 8C

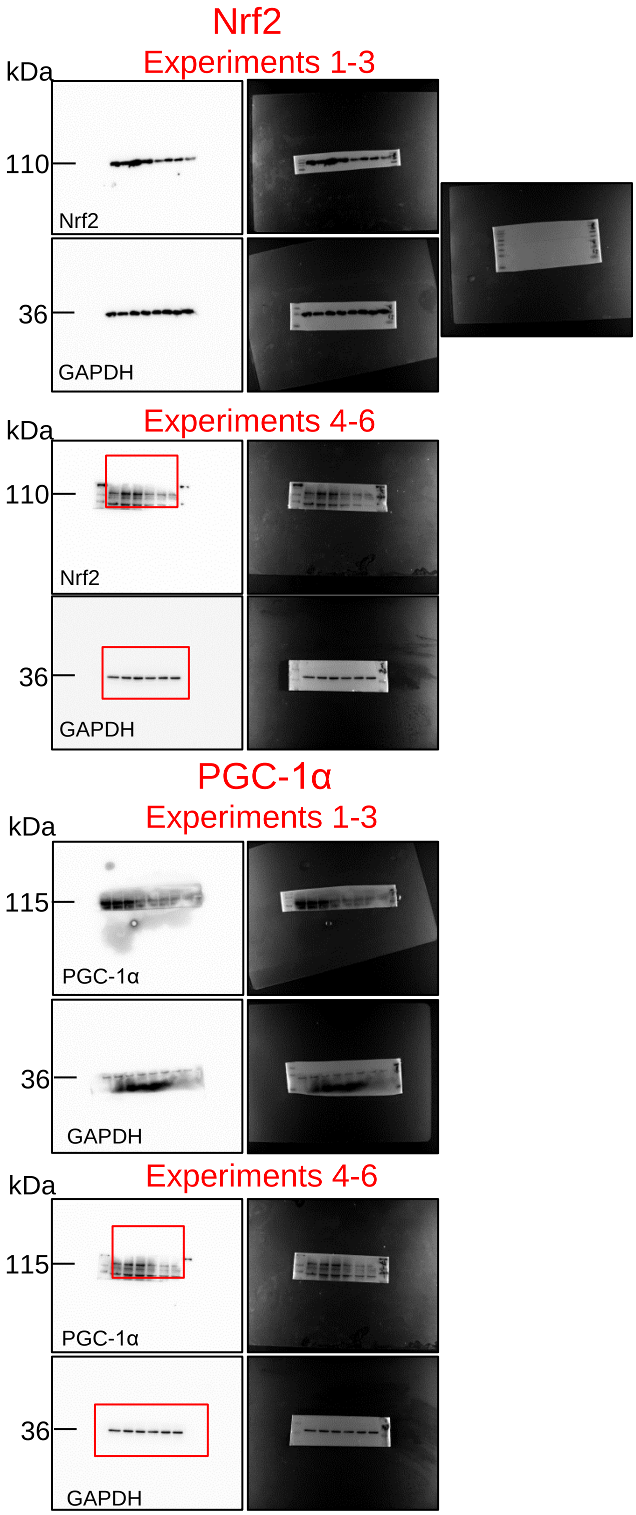

Figure S5A

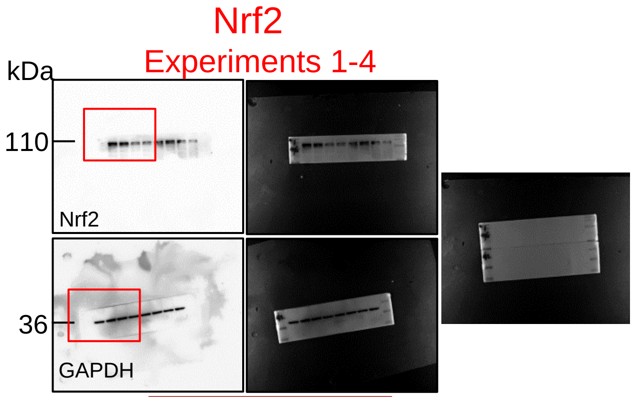

Figure S6A

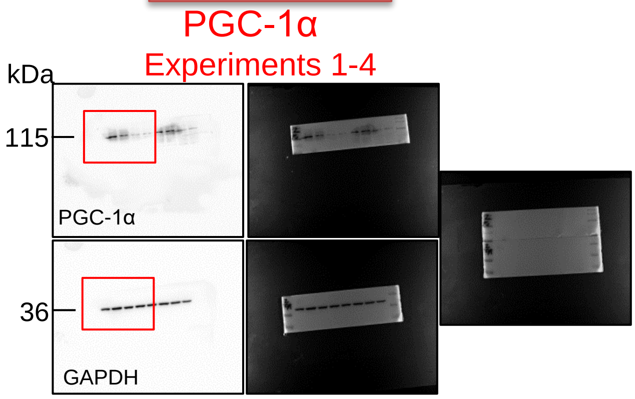

Figure S8

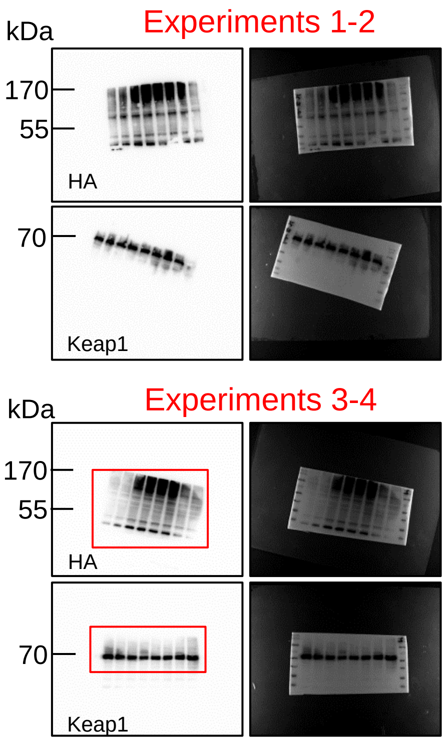
Experiments 1-2
kDa
170
55
HA
70
Keap1
Experiments 3-4
kDa
170
55
HA
70
Keap1
