# Supplementary material for: A dual Keap1 and p47phox inhibitor Ginsenoside Rb1 ameliorates high glucose/ox-LDL-induced endothelial cell injury and atherosclerosis
Source: Cell Death Dis. 2022 Sep 26;13(9):824. doi: 10.1038/s41419-022-05274-x (PMC9512801; doi:10.1038/s41419-022-05274-x)
Supplement: Supplementary file 2 — Agreement from all authors including additions [file 41419_2022_5274_MOESM2_ESM.pdf]

Confirmation letter from E-mail

Back | Reply | More

回复： Confirmation to addition of Professor Qi Lian-Wen as co-author of our article-CDDIS-22-2697RR

一晌贪欢 Sent to 孙海建

2022-09-14 18:35 Details

Dear Dr. Sun
I agree with this change.
Zi-Chao Wang

------------------ 原始邮件 ------------------

发件人: "sunhaijian927" <sunhaijian927@163.com>;
发送时间: 2022年9月14日(星期三) 晚上7:12
收件人: "一晌贪欢"<1714193881@qq.com>;"992692511"<992692511@qq.com>;"wuyj7112"<wuyj7112@163.com>;"869165515"<869165515@qq.com>;"zhouyebo666"<zhouyebo666@njmu.edu.cn>;
主题: Confirmation to addition of Professor Qi Lian-Wen as co-author of our article-CDDIS-22-2697RR

Dear all,
Since Professor Lian-Wen Qi provided great help in our revision process, we would like to add him as a co-author of our article "A dual Keap1 and p47phox inhibitor Ginsenoside Rb1 ameliorates high glucose/ox-LDL-induced endothelial cell injury and atherosclerosis (CDDIS-22-2697RR)". The authors are listed as follows: Zi-Chao Wang[1,2#], Kai-Ming Niu[1,2#], Yu-Jie Wu[1,2#], Kai-Rui Du[1], Lian-Wen Qi[1,2,3*], Ye-Bo Zhou[4*], Hai-Jian Sun[1,5*]
Do you agree to these changes?
Looking forward to your reply.
Best regards,
Hai-Jian Sun

Dr. Zi-Chao Wang

Back | Reply | More

Re: Confirmation to addition of Professor Qi Lian-Wen as co-author of our article-CDDIS-22-2697RR

武玉洁 Sent to 孙海建

2022-09-14 18:41 Details

Dear Dr. Sun
I agree with this change.
Yu-jie Wu

wuyj7112
邮箱： wuyj7112@163.com

---- Replied Message ----

From	sunhaijian927<sunhaijian927@163.com>
Date	09/14/2022 19:12
To	1714193881@qq.com<1714193881@qq.com>、992692511@qq.com<992692511@qq.com>、wuyj7112@163.com<wuyj7112@163.com>、869165515@qq.com<869165515@qq.com>、zhouyebo666@njmu.edu.cn<zhouyebo666@njmu.edu.cn>
Subject	Confirmation to addition of Professor Qi Lian-Wen as co-author of our article-CDDIS-22-2697RR

Dear all,
Since Professor Lian-Wen Qi provided great help in our revision process, we would like to add him as a co-author of our article "A dual Keap1 and p47phox inhibitor Ginsenoside Rb1 ameliorates high glucose/ox-LDL-induced endothelial cell injury and atherosclerosis (CDDIS-22-2697RR)". The authors are listed as follows: Zi-Chao Wang[1,2#], Kai-Ming Niu[1,2#], Yu-Jie Wu[1,2#], Kai-Rui Du[1], Lian-Wen Qi[1,2,3*], Ye-Bo Zhou[4*], Hai-Jian Sun[1,5*]
Do you agree to these changes?
Looking forward to your reply.
Best regards,
Hai-Jian Sun

Dr. Yu-Jie Wu

Back Reply More ▾

回复： Confirmation to addition of Professor Qi Lian-Wen as co-author of our article-CDDIS-22-2697RR

牛恺铭 Sent to 孙海建
2022-09-14 18:31 Details

Dear Dr. Sun
We agree with this change.
Kai-Ming Niu

------------------ 原始邮件 ------------------

发件人: "sunhaijian927" <sunhaijian927@163.com>;
发送时间: 2022年9月14日(星期三) 晚上7:12
收件人: "1714193881"<1714193881@qq.com>;"牛恺铭"<992692511@qq.com>;"wuyj7112"<wuyj7112@163.com>;"869165515"<869165515@qq.com>;"zhouyebo666"<zhouyebo666@njmu.edu.cn>;
主题: Confirmation to addition of Professor Qi Lian-Wen as co-author of our article-CDDIS-22-2697RR

Dear all,
Since Professor Lian-Wen Qi provided great help in our revision process, we would like to add him as a co-author of our article "A dual Keap1 and p47phox inhibitor Ginsenoside Rb1 ameliorates high glucose/ox-LDL-induced endothelial cell injury and atherosclerosis (CDDIS-22-2697RR)". The authors are listed as follows: Zi-Chao Wang[1,2#], Kai-Ming Niu[1,2#], Yu-Jie Wu[1,2#], Kai-Rui Du[1], Lian-Wen Qi[1,2,3*], Ye-Bo Zhou[4*], Hai-Jian Sun[1,5*]
Do you agree to these changes?
Looking forward to your reply.
Best regards,
Hai-Jian Sun

Back Reply More ▾

Re: Confirmation to addition of Professor Qi Lian-Wen as co-author of our article-CDDIS-22-2697RR

Du Kairui Sent to 孙海建
2022-09-14 18:25 Details

Dear Dr. Sun
We agree with this change.

---- Replied Message ----

From	sunhaijian927<sunhaijian927@163.com>
Date	09/14/2022 19:12
To	1714193881@qq.com<1714193881@qq.com>, 992692511@qq.com<992692511@qq.com>, wuyj7112@163.com<wuyj7112@163.com>, 869165515@qq.com<869165515@qq.com>, zhouyebo666@njmu.edu.cn<zhouyebo666@njmu.edu.cn>
Subject	Confirmation to addition of Professor Qi Lian-Wen as co-author of our article-CDDIS-22-2697RR

Dear all,
Since Professor Lian-Wen Qi provided great help in our revision process, we would like to add him as a co-author of our article "A dual Keap1 and p47phox inhibitor Ginsenoside Rb1 ameliorates high glucose/ox-LDL-induced endothelial cell injury and atherosclerosis (CDDIS-22-2697RR)". The authors are listed as follows: Zi-Chao Wang[1,2#], Kai-Ming Niu[1,2#], Yu-Jie Wu[1,2#], Kai-Rui Du[1], Lian-Wen Qi[1,2,3*], Ye-Bo Zhou[4*], Hai-Jian Sun[1,5*]
Do you agree to these changes?
Looking forward to your reply.
Best regards,
Hai-Jian Sun

Dr. Kai-Ming Niu

Dr. Kai-Rui Du

Back Reply

回复： Fw:Confirmation to addit
of Professor Qi Lian-Wen as co-
author of our article-CDDIS-22-
2697RR

Sender 841097196

Recipient Me

2022-09-14 19:17

Dear Dr. Sun

I agree with it.

Qi

------------------ 原始邮件 ------------------

发件人: "sunhaijian927" <sunhaijian927@163.com>
发送时间: 2022年9月14日(星期三) 晚上8:16
收件人: ' Qilw@cpu.edu.cn<Qilw@cpu.edu.cn>
主题: Fw:Confirmation to addition of Professor Qi L
s co-author of our article-CDDIS-22-2697RR

-------- Forwarding messages --------
From: "sunhaijian927" <sunhaijian927@163.c
Date: 2022-09-14 18:12:52
To: 1714193881@qq.com,992692511@qq.co
12@163.com,869165515@qq.com,zhouyebo
u.edu.cn
Subject: Confirmation to addition of Professo
Wen as co-author of our article-CDDIS-22-269
Dear all,
Since Professor Lian-Wen Qi provided g
in our revision process, we would like to
as a co-author of our article "A dual Kea
[illegible]

Professor. Lian-Wen Qi

Back Reply More ▾

Re:Confirmation to addition of
Professor Qi Lian-Wen as co-author
of our article-CDDIS-22-2697RR

Sender 税务

Recipient Me

2022-09-14 19:01 Details

Dear Professor Sun,
I agree this change that Professor Lian-Wen Qi
was added in this article as a co-author （CDDIS
-22-2697RR）.

Best regards,
Ye Bo, Zhou

发件人： sunhaijian927 <sunhaijian927@163.com>
发送日期： 2022-09-14 19:12:52
收件人： 1714193881@qq.com,992692511@qq.com,w
uyj7112@163.com,869165515@qq.com,zhouyebo666
@njmu.edu.cn
主题： Confirmation to addition of Professor Qi Lian-We
n as co-author of our article-CDDIS-22-2697RR
- Show cited text -

Dr. Ye-Bo Zhou
